# Early and late genome-wide gastric epithelial transcriptome response during infection with the human carcinogen *Helicobacterpylori*

**DOI:** 10.1016/j.cellin.2022.100032

**Published:** 2022-05-25

**Authors:** Irshad Sharafutdinov, Arif Ekici, Michael Vieth, Steffen Backert, Bodo Linz

**Affiliations:** a Department of Biology, Division of Microbiology, Friedrich Alexander Universität Erlangen-Nürnberg, Staudtstr. 5, D-91058, Erlangen, Germany; b Institute of Human Genetics, University Hospital, Friedrich Alexander Universität Erlangen-Nürnberg, Schwabachanlage 10, D-91054, Erlangen, Germany; c Institute of Pathology, Friedrich-Alexander-Universität Erlangen-Nürnberg, Klinikum Bayreuth, Preuschwitzer Str 101, D-95445, Bayreuth, Germany

**Keywords:** *Helicobacter*, Cancer, RNA-seq, HtrA, Apoptosis, Inflammation

## Abstract

Infection of the stomach by *Helicobacter pylori* is a major risk factor for the development of gastric cancer. Colonization of the gastric epithelium leads to the activation of multiple disease-related signaling pathways. Serine protease HtrA represents an important secreted virulence factor that mediates cleavage of cellular junctions. However, its potential role in nuclear responses is unknown. Here, we performed a genome-wide RNA-seq analysis of polarized gastric epithelial cells infected by wild-type (wt) and Δ*htrA* mutant bacteria. Fluorescence microscopy showed that *H. pylori* wt, but not Δ*htrA* bacteria, preferably localized at cellular junctions. Our results pinpointed early (2 h) and late (6 h) transcriptional responses, with most differentially expressed genes at 6 h post infection. The transcriptomes revealed HtrA-dependent targeting of genes associated with inflammation and apoptosis (e.g. *IL8*, *ZFP36*, *TNF*). Accordingly, infection with the Δ*htrA* mutant induced increased apoptosis rates in host cells, which was associated with reduced *H. pylori* CagA expression. In contrast, transcription of various carcinogenesis-associated genes (e.g. *DKK1*, *DOCK8*) was affected by *H. pylori* independent of HtrA. These findings suggest that *H. pylori* disturbs previously unknown molecular pathways in an HtrA-dependent and HtrA-independent manner, and provide valuable new insights of this significant pathogen in humans and thus potential targets for better controlling the risk of malignant transformation.

## Highlights

• *H. pylori* virulence factor HtrA enables the bacteria to localize at cellular junctions.
• Transcriptional changes are specific between early and late infection time points.
• HtrA impacts the transcription of host genes associated with inflammation and apoptosis.
• *H. pylori* HtrA promotes CagA expression upon infection.
• HtrA deficiency in *H. pylori* leads to higher apoptosis rates of host cells.

## Graphical abstract

**Figure:**
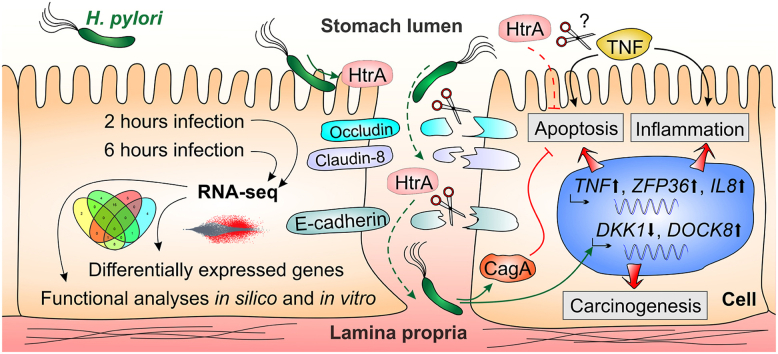

## 1 Introduction

About half of the world's population is carrying the gastric bacterium *Helicobacter pylori* that represents a high risk factor for developing gastric diseases including malignancies. This Gram-negative spiral-shaped bacterium naturally infects the human stomach, mostly asymptomatically; though in a subset of patients gastric disorders can develop. *H. pylori*-driven pathologies range from chronic active gastritis, peptic ulcer disease to gastric cancer and mucosa-associated lymphoid tissue (MALT) lymphoma (Kusters et al., 2006). Gastric disease outcome depends on the complex interaction of the host with the bacterium. Specific host genetic polymorphisms and gastric acid production control the colonization of the stomach, and antibiotics against *H. pylori* in patients suffering from gastritis and peptic ulcer markedly reduced the risk of recurring disease (Kuo et al., 2019; Treiber and Lambert, 1998). Individual *H. pylori* strains significantly vary in terms of their virulence potential and are divided into more virulent and less virulent groups (Covacci et al., 1997). Indeed, the severity of *H. pylori*-raised disorders widely depends on a range of bacterial factors. The most comprehensively studied *H. pylori* virulence factors associated with severe disorders are cytotoxin-associated gene A (CagA) and vacuolating cytotoxin A (VacA) (Atherton et al., 1995; Blaser et al., 1995; Tegtmeyer et al., 2017a). CagA is an effector protein injected into the host cell by a type IV secretion system (T4SS). This T4SS is encoded by the *cag* pathogenicity island (*cag*PAI) (Backert et al., 2017; Olbermann et al., 2010). Upon delivery into host cells, CagA disturbs host molecular signaling, which leads to cytoskeletal rearrangements, loss of cell polarity, and epithelial barrier disruption (Knorr et al., 2019; Naumann et al., 2017; Sharafutdinov et al., 2020). Another *H. pylori* virulence factor, the cytotoxin VacA, triggers cell vacuolization, pore formation, and apoptosis in infected cells (Chauhan et al., 2019; McClain et al., 2017). Other *H. pylori* virulence factors that have been extensively studied in the last decade are the high temperature requirement A (HtrA) serine protease, blood group antigen binding protein A (BabA), sialic acid binding protein A (SabA), *Helicobacter* outer membrane protein Q (HopQ), and outer-inflammatory protein A (OipA) (Ansari and Yamaoka, 2019; Backert et al., 2016; Javaheri et al., 2016). Among those, special interest was focused on HtrA as this protease is secreted into the supernatant by *H. pylori* in order to disrupt cellular junctions (Tegtmeyer et al., 2017b).

HtrA serine proteases are widely distributed in both prokaryotic and eukaryotic organisms. Human HtrAs are involved in the maintenance of cellular homeostasis, in stress response and in cell death, and disturbance of their function can result in neurodegenerative diseases, musculoskeletal disorders or tumorigenesis (Clausen et al., 2011). In bacteria, HtrA family members combine chaperone and proteolytic activities by proper folding or degradation of misfolded proteins, respectively (Clausen et al., 2011). HtrA activity is commonly found in the periplasm and is necessary for bacterial survival under stress conditions, as was shown, for instance, in *Escherichia coli* (Skorko-Glonek et al., 1999), *Listeria monocytogenes* (Wonderling et al., 2004) and *Streptococcus mutans* (Biswas and Biswas, 2005). Furthermore, bacterial HtrAs can play a major role in pathogenesis by either directly conflicting damage to host tissue or by maintaining other bacterial virulence factors. For example, a mutant of the human pathogen *Streptococcus pyogenes* with impaired HtrA expression showed reduced amounts of mature streptococcal pyrogenic exotoxin B (SpeB) (Cole et al., 2007). *Campylobacter jejuni*, *H. pylori* and *Bacillus anthracis* and probably more bacteria secrete HtrA into the extracellular space and therefore exert proteolytic activity on host proteins (Backert et al., 2018).

In *H. pylori* and other bacteria, protease HtrA exhibits both chaperone and proteolytic activities, providing bacteria with cell viability and contributing to bacterial virulence. Recently, the chaperone activity of HtrA was shown to play an essential role in *H. pylori* survival under thermal, pH and osmotic stress conditions (Zarzecka et al., 2019a, 2019b). Furthermore, the proteolytic activity of HtrA was required for its efficient secretion by *H. pylori*. Interestingly, genetic inactivation of *htrA* was associated with mutations in SecA, which is involved in protein translocation from the cytoplasm into the periplasm, suggesting a functional relationship between HtrA and the Sec translocation system in *H. pylori* (Zawilak-Pawlik et al., 2019). Biochemical analyses showed that *H. pylori*'s HtrA has a very high thermal stability *in vitro* and can restore its active structure after exposure to denaturing conditions (Zarzecka et al., 2019a, 2019b). These observations imply that HtrA is well adapted to both protein quality control in the bacterial periplasm and to pathogenesis when secreted. Secreted *H. pylori* HtrA was initially shown to target the host adherens junction protein E-cadherin via cleavage of its ectodomain (Hoy et al., 2010). Further analysis showed that the preferential HtrA cleavage sites in E-cadherin contain a [VITA]-[VITA]-x-x-D-[DN] sequence pattern (Schmidt et al., 2016). Finally, the tight junction proteins occludin and claudin-8 were identified as two additional substrates cleaved by HtrA during *H. pylori* paracellular transmigration (Tegtmeyer et al., 2017b). Remarkably, the *htrA* gene locus is highly conserved among *H. pylori* strains worldwide, implying a pivotal role of this protease for the bacterium (Tegtmeyer et al., 2016). However, how HtrA affects the host cellular signaling upon *H. pylori* infection of gastric cells remains widely unclear. To elucidate the affected by HtrA upstream regulators and pathways, we performed an RNA-seq analysis of MKN-28 gastric epithelial cells after 2- or 6-h infection with either *H. pylori* wt bacteria or with an isogenic Δ*htrA* mutant. The RNA-seq analysis revealed new host cellular targets affected by *H. pylori* in an HtrA-dependent manner, comprising inflammatory, carcinogenic and apoptotic processes, which is emphasizing the significance of the HtrA in the pathogenesis of *H. pylori*.

## 2 Results

### 2.1 RNA-seq of gastric epithelial cells infected with *H. pylori*

**Fig. 1:**
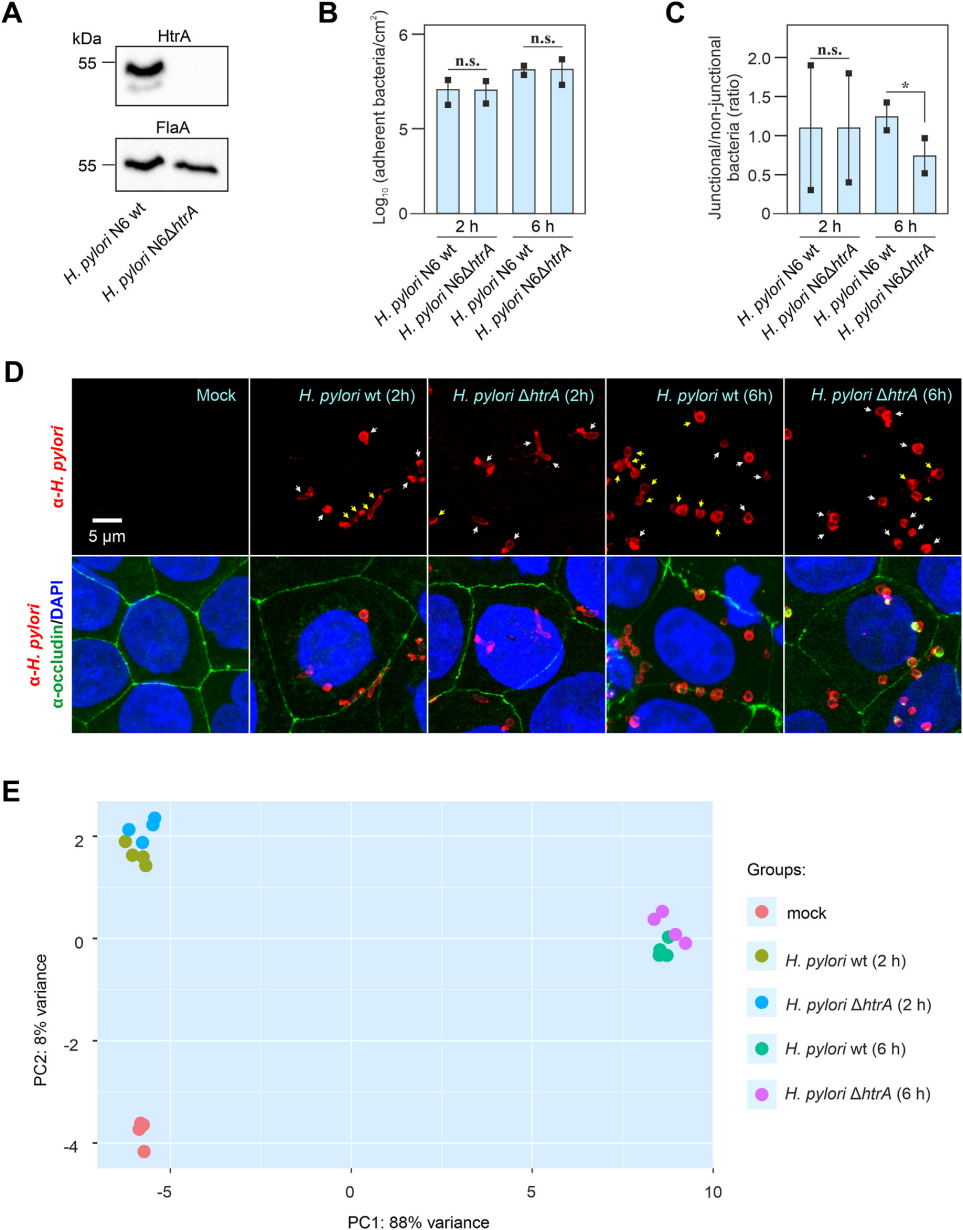
**Infection of the gastric epithelial MNK-28 cells by*****H. pylori*****andRNA-seqanalysis.** (**A**) Knockout of the HtrA protease gene in *H. pylori* strain N6 confirmed by Western blotting using α-HtrA and α-FlaA antibodies. (**B**) Quantification of bacterial cells adhered to MKN-28 monolayers based on microscopy analysis. Differences in number of adherent *H. pylori* wt and *H. pylori* Δ*htrA* cells were non-significant (n.s.) after either 2 or 6 h of infection. (**C**) Ratio of *H. pylori* wt and *H. pylori* Δ*htrA* cells showing non-junctional and junctional localization. Significant differences in ratios were defined as ∗ (p ​≤ ​0.05). n.s. – non-significant. (**D**) Confocal microscopy of MKN-28 cell monolayers infected with *H. pylori* N6 wt or Δ*htrA* mutant for 2 or 6 h. The samples were stained with α-*H. pylori*, α-occludin and DAPI to visualize bacterial cells (red), cellular tight junctions (green) and nuclei (blue), respectively. Arrows indicate bacterial cells at junctional (yellow arrows) or non-junctional (white) localization. (**E**) Principal component analysis (PCA) of rlog transformed read counts across all replicates in uninfected mock MKN-28 cells, MKN-28 infected with *H. pylori* wt or Δ*htrA* mutant for 2 or 6 h. The most variation (88%) is explained by the principal component 1 (PC1), showing dependency on the infection time, followed by 8% variation in PC2.

The present study was designed to identify the genome-wide affected genes as well as associated biological processes and molecular interaction networks in gastric MKN-28 cells upon infection with either the *H. pylori* N6 wild-type (wt) strain or an isogenic *H. pylori* N6Δ*htrA* mutant in which the protease gene was deleted (Fig. 1A). Upon infection, *H. pylori* wt and Δ*htrA* mutant showed similar bacterial loads on MKN-28 cells after 2 and 6 h of infection (Fig. 1B). In agreement with previous studies (Tegtmeyer et al., 2017b), wt bacteria tended to localize in the cell-to-cell junctions area, particularly after 6 h infection, in contrast to the Δ*htrA* mutant that clustered significantly less near the cell junctions (Fig. 1C and D). To determine the “early” and “late” transcriptomic response of polarized MKN-28 gastric epithelial cells during infection with *H. pylori*, samples for RNA-seq were collected after 2 and 6 h, respectively. Non-infected “mock” MKN-28 cells served as control. All experiments were performed in quadruple for statistical significance of the data. After quality filtering of the raw reads, on average 47,619,289 high-quality reads per sample were obtained, ranging from 40,628,500 to 56,376,713 reads (Table S1). On average, 43,084,974 reads mapped uniquely against the human reference genome (Ensembl GRCh37), of which 40,094,004 reads were counted over exons (84% of the raw reads). A sample-to-sample heatmap of DESeq2-normalized and log_2_ transformed counts across replicates revealed strong homogeneity among the replicates and absence of outlier samples (Fig. S1). Furthermore, a principal component analysis (PCA) of log_2_ transformed read counts showed a distinct difference between the uninfected control and cells infected for 6 h along PC 1, with 88% of the total variance explained along this axis (Fig. 1E). In contrast, the uninfected controls and the cells infected for 2 h separated along PC 2, which explained only 8% of the total variance, indicating that most transcriptomic changes occurred by 6 h post infection. Interestingly, the groups of MKN-28 cells infected with *H. pylori* N6 wt or with the N6Δ*htrA* deletion mutant clustered together at either time point, indicating only minor differences between the wt and Δ*htrA* groups in the host response to the bacterial infection.

**Fig. 2:**
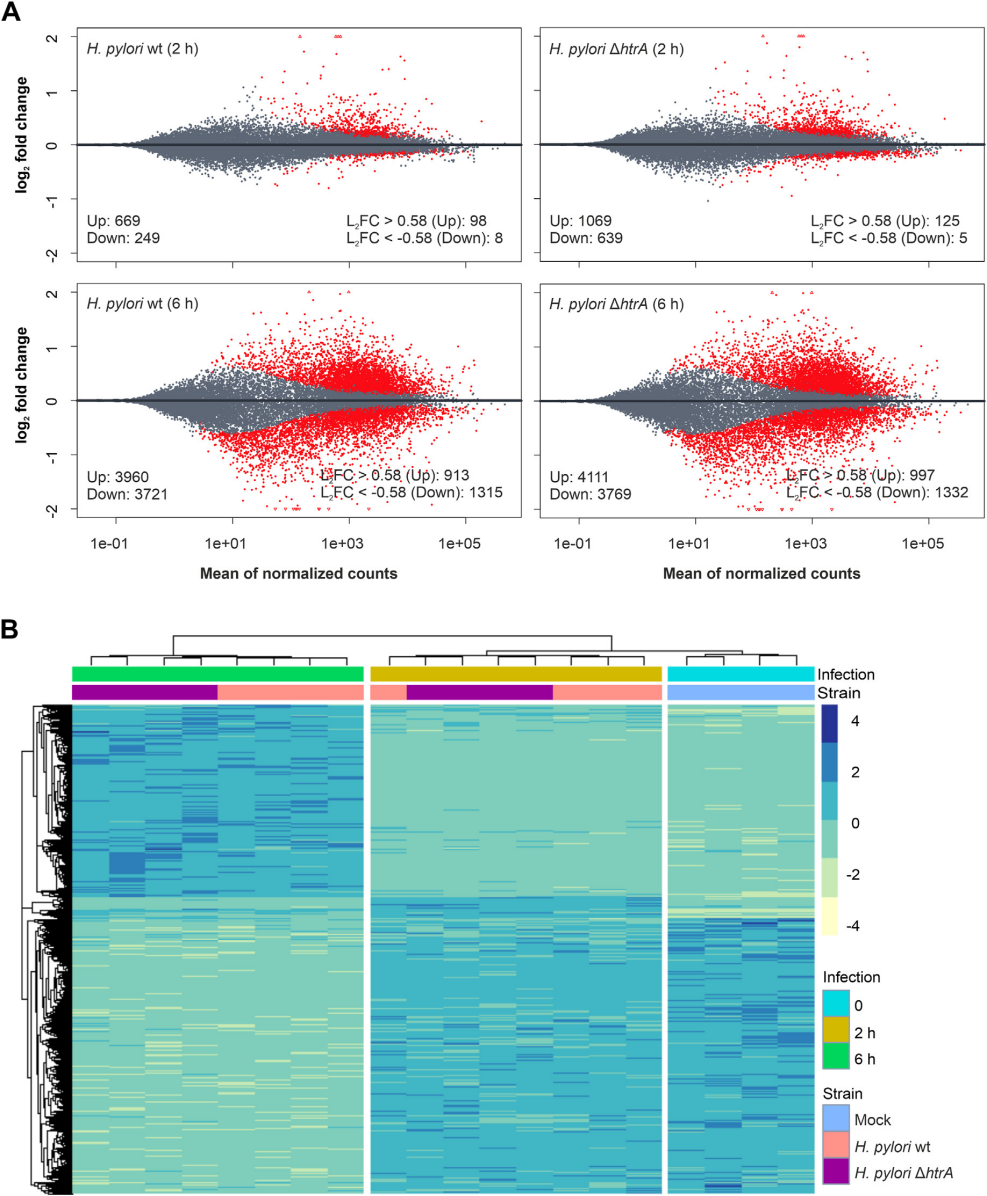
**Gene expression profiles of MKN-28 cells after infection with either*****H. pylori*****wt or Δ*****htrA*****mutant for 2 or 6 h.** (**A**) MA scatter plots of shrunk DEGs displaying log_2_ fold changes versus the mean of normalized expression counts from all samples. Left – total DEG numbers; Right – DEG numbers after adjusting to p value < 0.05 and log_2_ fold change of ≤ −0.58 or ≥0.58. (**B**) Clustered heatmap of 2722 DEGs with a log_2_ fold change of ≤ −0.58 or ≥ 0.58. L_2_FC – log_2_ fold change.

The increasing variance in gene expression over the mean (Fig. S2) suggested that the Negative Binomial distribution fits the count dataset best, while the Poisson distribution would result in an increased number of differentially expressed genes (DEGs) that are false-positive (Soneson and Delorenzi, 2013). Therefore, in order to assess the role of HtrA in the host response to *H. pylori* infection, a differential gene expression analysis was performed using the DESeq2 tool (Love et al., 2014). The final dispersion estimates clustered around the fitted trend line, which implied that data are well distributed and suitable for differential gene expression analysis (Fig. S3). To address the problem of false positive DEGs and reduce the false discovery rate (FDR), we used the DESeq2 default shrinkage estimator to identify and remove weakly expressed genes that showed relatively high variability between the replicates. The number of DEGs amounted to 918 genes (669 upregulated and 249 downregulated) and 1708 genes (1069 upregulated and 639 downregulated) in the groups infected for 2 h with the *H. pylori* wt strain or the Δ*htrA* mutant, respectively (Fig. 2A). In contrast, the number of DEGs after 6 h of infection was much higher, showing 7681 (3960 upregulated and 3721 downregulated) and 7880 (4111 upregulated and 3769 downregulated) DEGs, respectively.

### 2.2 RNA-seq reveals distinct gene expression profiles in gastric cells after 6 h of infection with *H. pylori* wt or Δ*htrA*

Differentially expressed genes that displayed a 1.5-fold change and thus a log_2_ fold change of ≥0.58 (upregulated genes) or ≤ −0.58 (downregulated genes) with a p value < 0.05 were selected for further analysis. This reduced the number of DEGs in the 2-h infection wt and Δ*htrA* groups to 106 genes (98 upregulated and 8 downregulated) and 130 genes (125 upregulated and 5 downregulated), respectively (Fig. 2A). The DEG numbers in the 6-h infection wt and Δ*htrA* groups were reduced to 2228 genes (913 upregulated and 1315 downregulated) and 2329 genes (997 upregulated and 1332 downregulated), respectively, suggesting progressive downstream signaling after infection. The total of 2722 different DEGs, which were present in at least one of the groups, were clustered and visualized in a heatmap (Fig. 2B). Similar to the PCA in Fig. 1E, the DEGs clustered into three groups; (I) uninfected mock control; (II) infected for 2 h; and (III) infected for 6 h (Fig. 2B). Interestingly, the groups infected with the *H. pylori* wt strain or the Δ*htrA* mutant sub-clustered into minimally distinct groups only after 6 h of infection, suggesting an increasing effect of the bacterial HtrA protease on the transcription of MKN-28 cells over time.

### 2.3 HtrA deficiency in *H. pylori* results in altered expression of host genes involved in immune responses and transcription activity

**Fig. 3:**
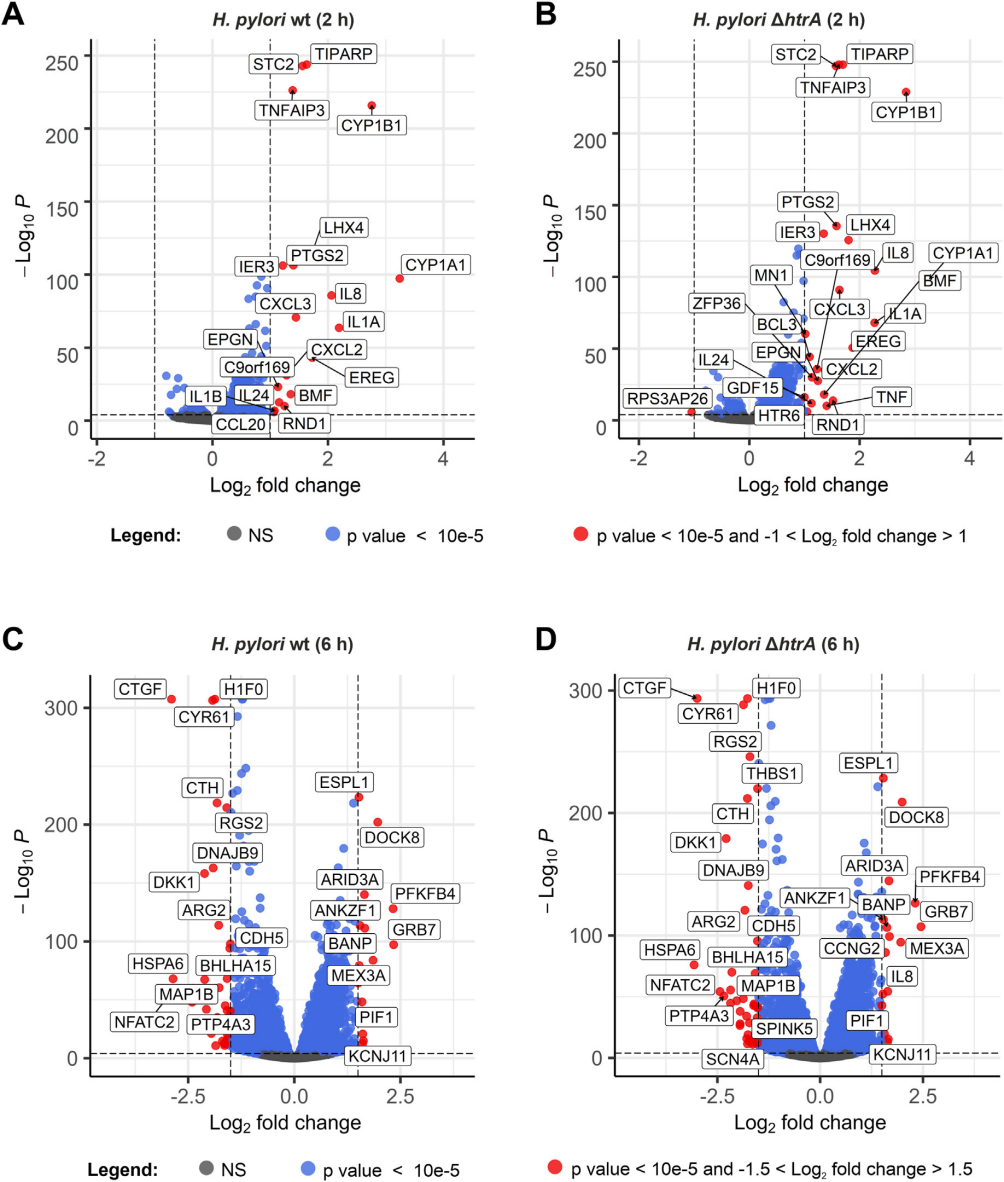
**The volcano plots of DEGs in MKN-28 cells after 2-h infection with *H. pylori* wt (A) or Δ*htrA* mutant (B) with cut-off values (dashed lines): p value = 10e-5, absolute log_2_ fold = 1**. Similarly, the DEGs were plotted after 6-h infection with *H. pylori* wt (**C**) or Δ*htrA* (**D**) with cut-off values: p value = 10^-5^, absolute log_2_ fold change = 1.5. The genes passing the threshold for p value and log_2_ fold change are shown with red dots and corresponding names.

To unravel the biologically most relevant genes in MKN-28 cells affected by *H. pylori*, we created a volcano plot, which plots the DEGs by their statistical significance versus the magnitude of change. For visualization, we selected a threshold of an absolute log_2_ fold change of ≥1.0 with a p value ≤ 10^−5^. After 2 h, infection with *H. pylori* wt or with the Δ*htrA* mutant upregulated a similar set of genes (Fig. 3A and B). As expected, the infection stimulated a strong immune response in MKN-28 cells, characterized by an over 2-fold increased transcription of genes *IL1A*, *IL8*, and *IL24* encoding inflammatory cytokines, and of chemokine receptor ligand genes *CXCL2*, *CXCL3* and *CCL20*. In addition, the bacterial infection activated transcription of *EREG* and *EPGN*, whose products are the ligands of the Epidermal Growth Factor Receptor (EGFR), as well as of *STC2*, *TIPARP*, *TNFAIP3*, *CYP1A1*, *CYP1B1*, *PTGS2*, *LHX4*, among others. Interestingly, the transcription profile of MKN-28 cells changed drastically after 6 h of infection by either *H. pylori* strain (Fig. 3C and D) with many more genes significantly upregulated or downregulated. As indicated above, the number of DEGs increased to 2228 and 2329 in the *H. pylori* wt and Δ*htrA* infection groups, respectively. Among the highly upregulated genes in both infection groups were *ESPL1*, *DOCK8*, *ARID3A*, *PFKFB4*, *ANKZF1*, *BANP* and *GRB7*, all of which exhibited a log_2_ fold change of ≥1.5. Significantly downregulated genes in both groups with a log_2_ fold change of ≤ −1.5 comprised *CTGF*, *H1F0*, *CYR61*, *CTH*, *RGS2*, *DKK1*, *DNAJB9*, *ARG2, CDH5*, and others.

**Fig. 4:**
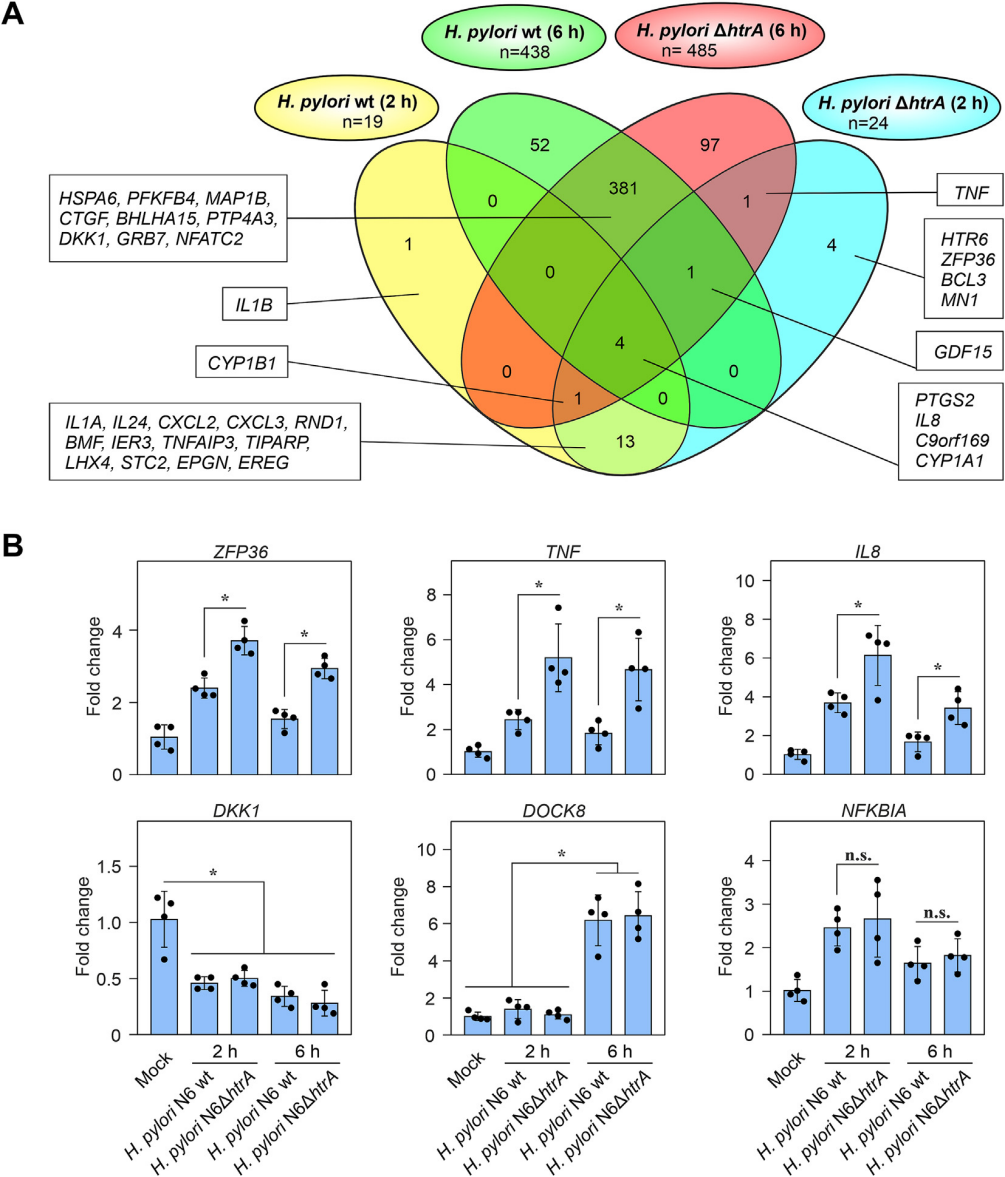
(**A) Venn diagram representing the shared DEGs in different groups of MKN-28 infected by *H. pylori* wt or Δ*htrA* mutant for 2 or 6 h (adjusted p value < 0.05, log_2_ fold change of ≤ −1 or ≥ 1)**. (**B**) Quantitative reverse transcription PCR (RT-qPCR) of representative MKN-28 genes associated with affected inflammatory (*IL8*, *ZFP36*, *NKFBIA*), apoptotic (*TNF*, *NKFBIA*), and carcinogenic (*DKK1*, *DOCK8*) pathways after infection with *H. pylori* wt and *H. pylori* Δ*htrA*. Significant differences are represented as ∗ (p ​≤ ​0.05); n.s. – non-significant.

Next, we aimed to define, which DEGs (adjusted p value < 0.05, log_2_ fold change of ≤ −1 or ≥ 1) were specific for the individual experimental groups (Fig. 4A, Table S2). Four genes were differentially expressed in all four infection groups, of which expression of *PTGS2*, *IL8* and *CYP1A1* were upregulated, while expression of *C9orf169* was upregulated at 2 h post infection, but downregulated at the 6 h time point. At the 2 h infection time point, expression of 13 genes (*IL1A*, *IL24*, *CXCL2*, *CXCL3*, *RND1*, *BMF*, *IER3*, *TNFAIP3*, *TIPARP*, *LHX4*, *STC2*, *EPGN*, *EREG*) was significantly upregulated in MKN-28 cells after infection with either wt or Δ*htrA* mutant *H. pylori* strains. Transcription of *IL1B* was elevated only in MKN-28 cells infected with *H. pylori* wt bacteria, while upregulation of *HTR6*, *ZFP36*, *BCL3* and *MN1* was specific to MKN-28 cells infected with the Δ*htrA* mutant. Considerably more, 381 DEGs, were shared between the wt and Δ*htrA H. pylori* infection groups at the 6 h time point, with the most significantly upregulated genes being *GRB7*, *PFKFB4*, *DOCK8*, and *MEX3A*, and the most significantly downregulated genes *PTP4A3*, *DKK1*, *BHLHA15*, *MAP1B*, *HSPA6, CTGF* and *NFATC2*. The number of strain-specific genes increased by the 6-h time point to 97 DEGs induced by the Δ*htrA* mutant bacteria and 52 DEGs induced by the wt bacteria. However, the change in expression of those genes was lower than for most of the 381 DEGs that were shared between the two groups. Interestingly, only one gene, *TNF*, was significantly upregulated in MKN-28 cells infected with the Δ*htrA* mutant at either time point, but not in cells infected with the *H. pylori* wt strain.

To validate the RNA-seq data, differential expression of a set of six genes that are involved in various biological processes as discussed below was checked by RT-qPCR of the same samples (Fig. 4B). These genes included *ZFP36*, *TNF*, and *IL8*, for all of which the RT-qPCR data confirmed elevated expression in Δ*htrA* mutant-infected compared to wt *H. pylori*-infected epithelial cells. In addition, the RT-qPCR analysis validated downregulation of *DKK1* expression in *H. pylori*-infected cells *versus* the uninfected control, upregulated *NFKBIA* expression compared to the uninfected control with slight differences between the 2 h and 6 h time points, and upregulation of *DOCK8* at the latter time point (Fig. 4B). Taken together, we identified the most significant DEGs in MKN-28 cells affected by either *H. pylori* wt or Δ*htrA*, as well as defined particular DEGs shared by the experimental groups.

### 2.4 Gene ontology (GO) enrichment analysis shows both unique and distinct biological pathways in MKN-28 cells affected by wt and Δ*htrA H. pylori*

**Fig. 5:**
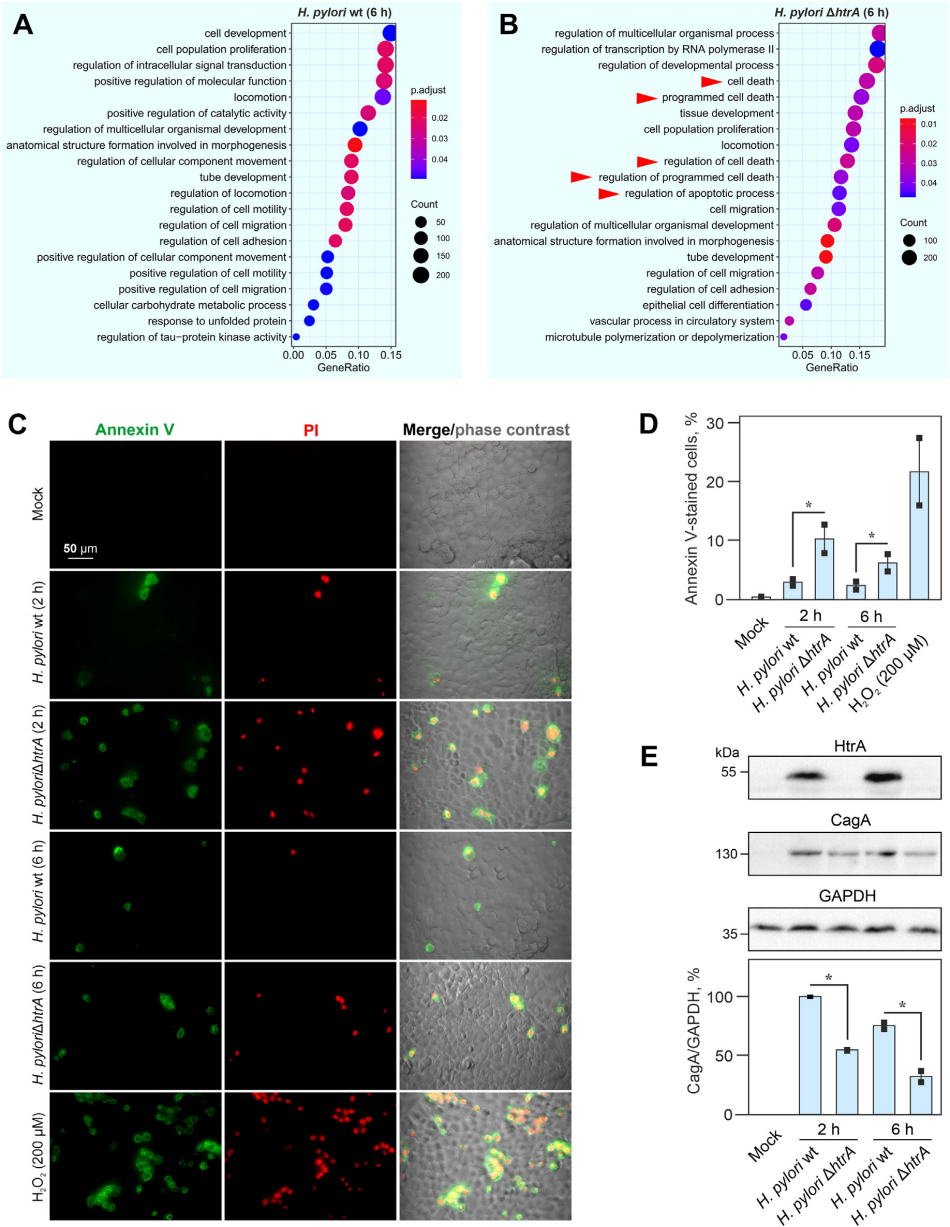
**Gene Ontology (GO) term enrichment of biological pathways in MKN-28 cells affected after 6-h infection with *H. pylori* wt (A) or Δ*htrA* (B)**. GO terms were ordered according to their gene ratio values which represents the number of DEGs connected to the corresponding GO term, divided by the total number of DEGs. The size of the dots represents the number of DEGs (Count) associated with the GO term and the color represents the adjusted p-value (p.adjust). (**C**) Epifluorescence microscopy of MKN-28 cell monolayers infected with *H. pylori* wt or Δ*htrA* mutant for 2 or 6 h and stained with annexin V and propidium iodide (PI) to visualize apoptotic (green) and necrotic (red) cells, respectively. The right panel shows the annexin V and PI staining merged with phase contrast imaging. Treatment with 200 μM H_2_O_2_ served as positive control. (**D**) Quantification of annexin V-stained apoptotic cells as the percentage of total cells assessed by epifluorescence/phase contrast microscopy. Δ*htrA* mutant-infected cells showed a significantly (∗; p ​≤ ​0.05) higher apoptosis rate. (**E**) Western blot of HtrA and CagA to show the potential role of HtrA in production of CagA by *H. pylori* and GAPDH (loading control) normalized expression of CagA by wt or Δ*htrA* mutant *H. pylori* during infection of MKN-28 monolayers. Asterisks (∗) denote significant differences (p ​≤ ​0.05).

To assess the functional importance of *H. pylori* infection on the MKN-28 whole-genome transcriptome, we performed a functional analysis of significant DEGs by using an over-representation analysis. First, we enriched known Gene Ontology (GO) terms in ClusterProfiler (Yu et al., 2012) using the corresponding lists of significant DEGs (adjusted p value < 0.05, log_2_ fold change < −0.58 or >0.58). The most enriched GO terms upon infection with *H. pylori* wt for 2 h included the biological processes “Response to bacterium” (GO:0009617) and “Positive regulation of protein kinase activity” (GO:0045860). “Response to bacterium” was characterised by enrichment for genes encoding cytokines (e.g. *IL24*, *IL1A*, *IL1B, IL8*) and other proinflammatory regulators (e.g. *IRAK2*, *WNT5A*, *CXCL2*, *CXCL3*, *TNFAIP3*, *ZFP36, SASH1*, *ANKRD1*, *NFKBIA, TNFRSF11A, CEBPB*, *BCL3* and *ICAM1*) as well as metabolic enzymes involved in stress response (e.g. *CYP1A1* and *SOD2*). The category “Positive regulation of protein kinase activity” was enriched with *TGFA*, *IL1B*, *IRAK2*, *WNT5A*, *EPGN*, *EREG*, *EGR1*, *CDKN1A*, *SASH1*, *LPAR1*, *ERCC6*, *DKK1*, *TNFRSF11A*, *SDC4*, *GDF15* and *CARD10* (Fig. S4; Table S3). When MKN-28 cells were infected with the Δ*htrA* mutant for 2 h, the enriched GO terms were similar to those in MKN-28 cells infected with the *H. pylori* wt strain (Fig. S4; Table S4). However, a biological process was defined that was distinctly affected by the Δ*htrA* mutant after 2 h, namely “cellular response to tumor necrosis factor” (GO:0071356), which was characterized by enrichment for DEGs *ZC3H12A*, *NFE2L2*, *IL8*, *TNF*, *TNFAIP3*, *TRAF1*, *ANKRD1*, *KCNJ11*, *BIRC3*, *THBS1*, *NFKBIA*, *TNFRSF11A*, *ICAM1* and *ZFP36*. A further analysis of the transcriptionally upregulated genes showed major changes at the functional levels after 6 h of infection, with the major biological processes “Cell population proliferation” (GO:0008283), “Regulation of cell migration” (GO:0030334) and “Regulation of intracellular signal transduction” (GO:1902531) (Fig. 5A, Table S5). Similar to infection with wt bacteria, the enriched GO terms after 6 h infection with the Δ*htrA* included “Regulation of cell migration” and “Cell population proliferation” (Fig. 5B, Table S6). However, the enriched GO terms representing “Cell death” or “Apoptotic process” (GO:0010941, GO:0008219, GO:0043067 and GO:0012501) were distinctly present after the extended infection with the Δ*htrA* mutant, suggesting that *H. pylori* HtrA may be implicated in the inhibition of host cell apoptotic pathways.

Since the data indicated a potential role of HtrA in apoptosis, we studied the induction of apoptosis in MKN-28 cells infected with either *H. pylori* wt or the Δ*htrA* mutant. In agreement with the GO enrichment analysis, *H. pylori* Δ*htrA* infection resulted in an higher apoptosis rate than infection with wt bacteria after both 2 h or 6 h infection (Fig. 5C and D). Among the bacterial factors that could explain such an effect on apoptosis is *H. pylori* oncoprotein CagA which is known to exhibit anti-apoptotic properties upon infection (Mimuro et al., 2007; Backert and Tegtmeyer, 2017). In our experiments, we found that infection of MKN-28 with *H. pylori* wt bacteria resulted in ∼2-fold higher CagA expression at both 2 and 6 h post infection compared to infection with the *H. pylori* Δ*htrA* mutant (Fig. 5E), suggesting a crucial role of HtrA in CagA delivery. Therefore, it seems that HtrA mediates the downstream signaling in MKN-28 cells upon *H. pylori* infection at least partially through CagA.

### 2.5 Ingenuity Pathway analysis (IPA) reveals HtrA-dependent activation and inhibition of various upstream regulators

**Fig. 6:**
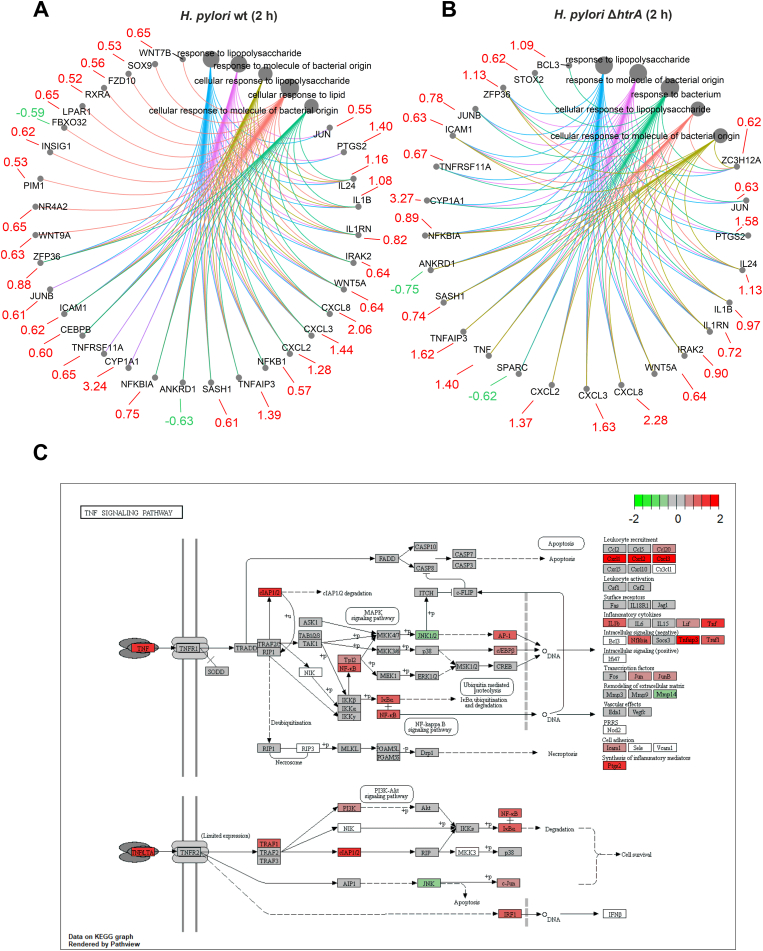
**The core enriched genes linked to molecular functions (GO terms) in MKN-28 cells infected by *H. pylori* wt (A) or Δ*htrA* mutant (B) for 2 h**. The node size reflects the gene number involved in the process. The red and green numbers associated with genes indicate the up- and down-regulation levels in log_2_ fold change. (**C**) TNF signaling pathway (hsa04668) affected by *H. pylori* Δ*htrA* infection for 2-h was enriched using the KEGG enrichment assay. Red and green colors reflect up- and down-regulation levels in log_2_ fold change, respectively.

The results of differential gene expression analysis and RT-qPCR along with the GO enrichment assay indicated a possible role of HtrA in TNF signaling in MKN-28 cells after 2 h of *H. pylori* infection. Therefore, we aimed to investigate the molecular interactions in MKN-28 cells upon infection with the Δ*htrA* mutant in more detail. The most activated molecular functions in MKN-28 cells upon infection with *H. pylori* wt for 2 h after GO enrichment analysis appeared to be “Cytokine activity”, “Cytokine receptor binding”, “Receptor ligand activity”, “Receptor regulator activity” and “Signaling receptor activator activity” (Fig. 6A), all of which correspond to the affected biological process “Response to bacterium” (Fig. S4). Thus, infection with *H. pylori* wt bacteria triggers a quick response of the host that results in activation of transcription factor genes *JUN* and *NFKB1* (Fig. 6A). Activation of these transcription factors may lead to expression of various cytokines genes such as *IL1B*, *CXCL2* and *CXCL3*, which in turn recruits immune cells to the site of infection. The Δ*htrA* deletion mutant activated similar cellular functions in the “Response to bacterium” category (Fig. 6B). However, infection with the Δ*htrA* mutant, but not with the wt bacteria, resulted in enrichment of genes involved in tumor necrosis factor (TNF) signaling, including *TNF* (encoding tumor necrosis factor), *TNFAIP3* (tumor necrosis factor alpha-induced protein 3), *TNFRSF11A* (tumor necrosis factor receptor superfamily member 11 A) and *ZFP36* (anti-inflammatory modulator Zinc finger protein 36). We further assessed the TNF signaling pathway (hsa04668) in MKN-28 cells affected by Δ*htrA* mutant infection using the KEGG enrichment pathway analysis. The TNF signaling pathway overview indicated that TNF activates transcription of downstream genes *AP1*, *NFKB* and *PI3K* and a set of chemokine genes including *CXCL1*, *CXCL2*, *CXCL3*, and *IL1B*, while expression of *JNK* and *MMP14* was slightly inhibited (Fig. 6C). Thus, the analysis of DEGs using GO and KEGG enrichment assays indicated a likely role of the HtrA protease in the TNF-α response to infection with *H. pylori*.

**Fig. 7:**
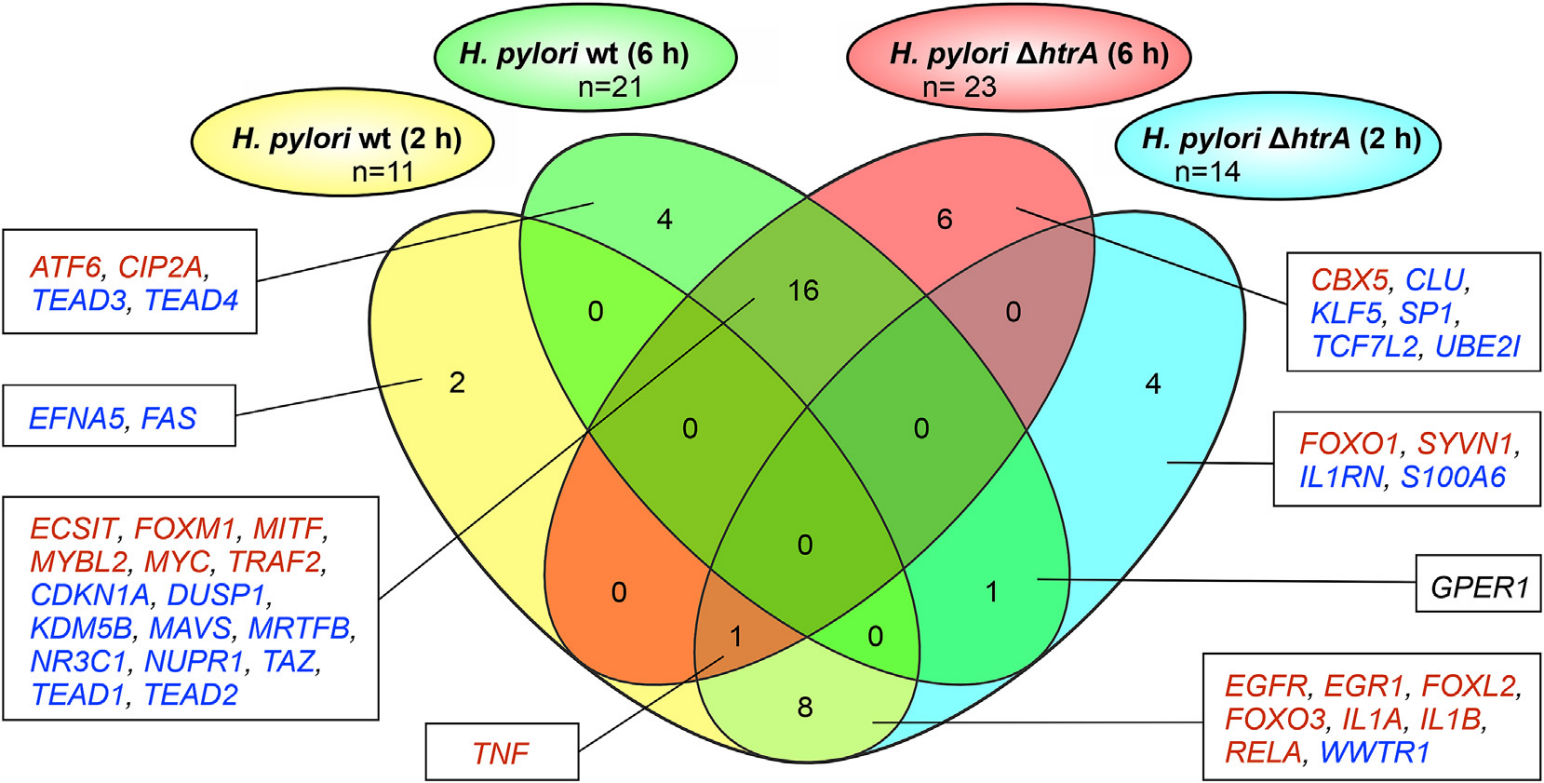
**The most significant upstream regulators in MKN-28 cells affected by *H. pylori* infection as determined by the Ingenuity Pathway Analysis (IPA)**. The threshold for including upstream regulators in the analysis was designed for the activation Z-score (activation state of an upstream regulator based on the regulation direction associated with the relationship between the regulator and the corresponding DEGs): <2 (inhibited) or >2 (activated). The P value of overlap (significance of overlap between the dataset genes and the genes that are regulated by an upstream regulator) < 0.05 was considered as significant. The red or blue colored genes correspond to the activated or inhibited transcription regulators, respectively. The *GREP1* gene (in black) was activated in MKN-28 infected by Δ*htrA* mutant for 2 h, but downregulated after 6 h infection with *H. pylori* wt.

In order to analyze the observed changes in MKN-28 gene expression upon *H. pylori* infection, we next attempted to pinpoint the upstream transcriptional regulators and their activation state using Ingenuity Pathway analysis (IPA) (Kramer et al., 2014). To identify the affected upstream regulators, IPA utilizes prior knowledge from its own database to predict expected effects between transcriptional regulators and their target genes, while taking into account the directional change of the expression. Thus, up- or down-regulation of particular genes would indicate activation or inhibition of the corresponding regulators upstream of these genes. After 2 h of infection, the transcriptional upstream regulators *EGFR*, *EGR1*, *FOXL2*, *FOXO3*, *IL1A*, *IL1B* and *RELA* were activated in MKN-28 cells infected with either *H. pylori* wt or the Δ*htrA* mutant, while *WWTR1* was inhibited (Fig. 7, Tables S7 and S8). The *EFNA5* and *FAS* genes were downregulated in MKN-28 cells only after 2-h infection with the *H. pylori* wt strain. In contrast, infection with the Δ*htrA* mutant resulted in activation of *FOXO1* and *SYVN1*, but inhibited *IL1RN* and *S100A6*. After 6-h infection, both *H. pylori* wt and Δ*htrA* mutant led to activation of *ECSIT*, *FOXM1*, *MITF*, *MYBL2*, *MYC* and *TRAF2* genes (Fig. 7, Tables S9 and S10). On the other hand, both bacteria inhibited the upstream regulators *CDKN1A*, *DUSP1*, *KDM5B*, *MAVS*, *MRTFB*, *NR3C1*, *NUPR1*, *TAZ*, *TEAD1* and *TEAD2*. Six hours of infection with the *H. pylori* wt strain activated upstream regulators *ATF6* and *CIP2A* while *TEAD3* and *TEAD4* were inhibited. During infection with the Δ*htrA* mutant, *CBX5* was activated and *CLU*, *KLF5*, *SP1*, *TCF7L2* and *UBE2I* were inhibited. Finally, the *TNF* upstream regulator was activated in all experimental groups with the exception of 6-h infection with the *H. pylori* wt strain.

## 3 Discussion

Early work on the interference of *H. pylori* infection with the host at the transcriptional level was mainly based on microarrays (Guillemin et al., 2002; Israel et al., 2001; Mills et al., 2001; Pachathundikandi et al., 2013). For example, Mueller and co-workers analyzed gene expression profiles to predict histopathological stages in a mouse model of MALT lymphoma (Mueller et al., 2003); DNA microarrays of gastric biopsies obtained from *H. pylori*-infected rhesus macaques provided new insights in host factors involved in pathogenesis (Huff et al., 2004); and a set of host genes was identified to be transcribed independently of the phosphorylation state of the CagA protein, a major *H. pylori* virulence factor (El-Etr et al., 2004). Later, the genome-wide map of *H. pylori* transcriptional start sites and operons was described by using differential RNA-seq (Sharma et al., 2010), which subsequently led to the discovery of small RNAs controlling expression of the major *H. pylori* virulence factors CagA and VacA (Eisenbart et al., 2020). Previous work using transcriptomic and proteomic approaches mainly focused on studying the disruption of host molecular pathways by *H. pylori* CagA and/or its associated T4SS (Chichirau et al., 2020; El-Etr et al., 2004; Glowinski et al., 2014; Guillemin et al., 2002; Wen et al., 2007). Furthermore, RNA-seq has been used to study the global immune responses to *H. pylori* in B cells (Chichirau et al., 2020) and macrophages (Tubau-Juni et al., 2020). In the present study, we aimed to investigate changes in molecular signaling in human gastric epithelial cells in response to *H. pylori* infection, with special focus on serine protease HtrA. Since the most significant changes in transcriptional regulation upon *H. pylori* infection appear at first hours, we aimed to analyze gastric MKN-28 cells after either 2 or 6 h of infection.

HtrA proteases are known to play a vital role in bacterial survival, in particular under stress conditions (Hansen and Hilgenfeld, 2013), but in some microbes they are also known as important virulence factors. HtrAs mediate secretion of other virulence factors, for example in *Streptococcus pyogenes*, or contribute to biofilm formation as was shown for *S. mutans* (Backert et al., 2018; Biswas and Biswas, 2005; Cole et al., 2007). Another function of HtrA involves direct damage of cellular tight junctions of the host epithelium, for instance by *Salmonella enterica*, *Shigella flexneri*, *C. jejuni* or *H. pylori* (Harrer et al., 2017, 2019; Hoy et al., 2012).

The most prominent changes in the MKN-28 transcriptome in response to *H. pylori* infection occurred between the 2-h and 6-h infection time points, independent of the *htrA* gene (Fig. 1). However, significant changes were observed in the number of DEGs at the 6-h time point, which was higher in MKN-28 cells infected with *H. pylori* Δ*htrA* (n = 7880; adjusted n = 2329) compared to infection with the wt bacteria (n = 7681; adjusted n = 2228) (Fig. 2A). Interestingly, after adjusting for p ≤ 0.05 and log_2_ fold change of ≤ – 0.58 and ≥0.58, the significant DEGs (n = 2722) sub-clustered the infections with *H. pylori* wt and Δ*htrA* mutant into two distinct groups, even though the difference was minor (Fig. 2B). The separation of these two groups may possibly be explained by the effect of accumulating HtrA after secretion by the bacteria. Each individual *H. pylori* cell constantly secretes the HtrA protein, which results in a steady increase of the secreted HtrA amount over time (Neddermann and Backert, 2019). The bacteria replicate, and more bacterial cells secrete more HtrA molecules, which results in an increasing effect of HtrA on the MKN-28 expression profile, possibly both directly and indirectly. HtrA damages the cell junctions by cleavage of the adherens junction protein E-cadherin and tight junction proteins claudin-8 and occludin, which triggers a cellular response. In addition and possibly even more important, damage of the cell junctions allows paracellular transmigration of *H. pylori* through the opened junctions to the basolateral side of the epithelium where *H. pylori* employs its T4SS to deliver the oncogenic effector protein CagA into the host cell (Tegtmeyer et al., 2017b).

A more detailed analysis of DEGs indicated on a strong immune response in MKN-28 cells after 2-h infection based on increased transcription of *IL1A*, *IL8*, *IL24*, *CXCL2*, *CXCL3* and *CCL20* (Fig. 3A and B). These data are in line with previous reports that indicated a strong IL-8 expression in response to *cag*PAI-positive *H. pylori* (Crabtree et al., 1995; Eftang et al., 2012). In our analysis, *IL8* expression was also among the most affected at both 2- and 6-h infection time points, confirming its major role in *H. pylori*-induced inflammation. Interestingly, expression of the chemokine (C-X-C motif) receptor 2 (CXCR2) ligands *CXCL2* and *CXCL3* has been recently shown to contribute to the malignant progression of gastric cancer (Yamamoto et al., 2019). In addition, CC chemokine ligand 20 (CCL20) expression was shown to be upregulated by *H. pylori* in an *cag*PAI-dependent but CagA-independent manner (Yoshida et al., 2009). In our experiments, the pro-inflammatory response of MKN-28 gastric cells after 2-h infection was comparable between *H. pylori* wt and Δ*htrA* mutant infections. Both wt and Δ*htrA* strains activated transcription of the *EREG* and *EPGN* genes both of which contribute to stimulation of the epidermal growth factor receptor gene *EGFR*. Elevated epiregulin (*EREG*) expression predicts poor prognosis in gastric cancer (Xia et al., 2019), while epigen (*EPGN*) is suspected to play a role in the development of lung carcinomas (Fujimoto et al., 2005).

The 6-h infection time point was marked by downregulation of *CTGF*, *H1F0*, *CYR61*, *CTH*, *RGS2*, *DKK1*, *DNAJB9*, *ARG2* and *CDH5*. Of interest, Dickkopf-related protein 1 (DKK1), an important inhibitor of carcinogenic Wnt signaling, was recently shown to be inhibited in intestinal metaplasia via promoter methylation (Lu et al., 2020). This study reported DKK1 downregulation in response to bile acid stimulation, while we observed *DKK1* downregulation after exposure to *H. pylori*. In addition, *H1F0,* which encodes histone H1.0, was shown to be silenced in various cancers (Torres et al., 2016) and could be a potential target molecule during *H. pylori* pathogenesis. Numerous other genes such as *ESPL1*, *DOCK8*, *ARID3A*, *PFKFB4*, *ANKZF1*, *BANP* and *GRB7* showed elevated expression rates, some of which are known or suspected to be involved in cancer development and/or progression. Of those, dedicator of cytokinesis 8 (DOCK8) is a guanine nucleotide exchange factor (GEF) that activates a number of small G proteins, including Rac1 and Cdc42, which are known to be manipulated by *H. pylori* during rearrangement of the actin cytoskeleton of the host cell (Churin et al., 2001), suggesting that increased *DOCK8* transcription might exacerbate the effect. The Warburg pathway enzyme 6-phosphofructo-2-kinase/fructose-2,6-bisphosphatase 4 (PFKFB4) is a kinase of the sugar metabolism that affects gene regulation by activating transcription of the oncogenic steroid receptor coactivator-3 (SRC-3), which promotes the development of tumour metastases in breast cancer (Dasgupta et al., 2018). Elevated *PFKFB4* transcription levels strongly correlated with shorter disease-free survival and overall breast cancer survival (Yao et al., 2019). Finally, growth factor receptor bound 7 (*GRB7*), a multidomain adaptor protein that was previously shown to interact with the *H. pylori* CagA effector protein (Selbach et al., 2009), is a critical mediator of EGFR/ErbB signaling involved in cancer development (Chu et al., 2019).

Interestingly, MKN-28 cells infected with Δ*htrA* at both 2-h and 6-h time points exhibited increased *TNF* expression implying that HtrA may be involved in TNF signaling, a view that was supported by the observed deregulation of *ZFP36*, *BCL3* and *TNF* during infection with the Δ*htrA* mutant. In addition, a GO enrichment assay of *H. pylori*-induced biological pathways revealed a distinct, Δ*htrA*-specific group of enriched GO terms that represented “Cell death” or “Apoptotic process”, suggesting that *H. pylori*'s HtrA might interfere with host cell apoptosis. Considering that TNF plays a crucial role in the regulation of both NF-κB-induced inflammation and caspase-mediated apoptosis (Lee et al., 2016), disturbance of the TNF pathway by HtrA explains the observed enrichment of apoptosis GO terms. In agreement, analysis of the upstream transcriptional regulators using IPA indicated downregulation of the *EFNA5* and *FAS* upstream regulators by *H. pylori* wt, but not Δ*htrA* bacteria. Fas, a member of the tumor necrosis factor receptor superfamily, is a cell surface “death receptor” which upon activation induces caspase-mediated apoptosis (Meynier and Rieux-Laucat, 2019). *H. pylori* appears to inhibit Fas receptor expression in an HtrA-dependent manner, which leads to inhibition of Fas-mediated apoptosis, underpinning the above observations. In addition, infection with the *H. pylori* Δ*htrA* mutant led to activation of the FOXO1 upstream regulator, another transcription factor involved in apoptosis regulation. Interestingly, FOXO1 was previously shown to be deregulated upon *H. pylori* infection (Tabassam et al., 2012). *H. pylori* wt infection activated the Cancerous Inhibitor of PP2A (CIP2A) upstream regulator that is overexpressed in gastric cancer. Intriguingly, CIP2A expression was shown to depend on *H. pylori* CagA (Zhao et al., 2010), however, HtrA might represent another bacterial factor disturbing the CIP2A pathway. Other upstream regulators such as CLU, KLF5, SP1 and TCF7L2 that were inhibited by Δ*htrA* mutant infection have also been previously shown to be involved in apoptosis regulation (Deniaud et al., 2006; Li et al., 2014, 2018; Mustafi et al., 2017). *H. pylori* Δ*htrA*-inhibited SP1 likely decreased expression of the downstream cystathionine γ-lyase (CTH) as observed above. In this regard, *H. pylori* was recently shown to evade the host immune response by inducing CTH in macrophages (Gobert et al., 2019), and therefore, HtrA could play a role in SP-mediated CTH expression. Finally, infection with *H. pylori* Δ*htrA* activated *TNF* throughout the infection, which substantiates the above-discussed considerations on the potential role of HtrA in the TNF-mediated inflammatory response.

We suggest that HtrA may affect host cellular signaling by either direct interactions with host target molecules or, more likely, by facilitating enhanced delivery of CagA and/or other virulence factors into the host cytoplasm. Indeed, HtrA-dependent cleavage of cellular junctions was shown to promote *H. pylori* paracellular transmigration, which facilitates basolateral injection of CagA into the epithelial cells (Tegtmeyer et al., 2017b). Alternatively, HtrA might affect cellular signaling directly via cleavage of E-cadherin, a tumour suppressor protein, the disruption of which appears to play a significant role in carcinogenesis signaling. In addition, we propose that the proteolytic activity of HtrA might also be involved in interfering with the TNF pathway. For instance, trypsin-like proteases were previously shown to cleave the TNF molecule from the N-terminus that subsequently resulted in its inactivation (Nakamura and Komiya, 1996). Proteolysis of TNF was also shown by a cysteine protease from the pathogen *Porphyromonas gingivalis*, confirming the biological relevance of such a process (Park et al., 2016). Thus, functional inactivation of TNF through cleavage by HtrA might also contribute to the observed host cell apoptosis. The mechanism(s), by which HtrA affects host signaling, whether directly or indirectly, should be unraveled in further studies. Summarizing the above discussed observations, we propose that upon *H. pylori* infection, secreted HtrA in addition to the tight junctions cleavage, significantly contributes to disturbance of host cellular signaling, which overall impacts disease development.

## 4 Materials and methods

### 4.1 Cell line, bacteria and culture conditions

Human MKN-28 cells (JCRB, #0253), originally isolated from gastric adenocarcinoma, were used in this work for the infection experiments. Cells were cultured in RPMI-1640 medium, containing 4 mM glutamine (Invitrogen, Karlsruhe/Germany), and 10% FCS (Invitrogen, Karlsruhe/Germany) at 37 °C. MKN-28 cells were grown until the formation of proper monolayers as described (Tegtmeyer et al., 2017b). Briefly, the cells were cultured on cell culture inserts with 3 μm pore size (Millipore, Burlington, Massachusetts, USA) to confluent monolayers and were subsequently incubated for another 14 days to allow for cell polarization. A transepithelial electrical resistance (TER) of ≥150 Ω/cm^2^ indicated the formation of polarized cell layers (Boehm et al., 2012). *H. pylori* strain N6 and *H. pylori* N6Δ*htrA* (Zawilak-Pawlik et al., 2019) with protease knockout were grown on horse serum GC agar plates supplemented with nystatin (1 μg/mL), vancomycin (10 μg/mL) and trimethoprim (5 μg/mL), and if necessary with 4 μg/mL chloramphenicol.

### 4.2 Western blot

MKN-28 monolayers or *H. pylori* cells intended for protein analysis were subjected to SDS-PAGE followed by Western blotting (Burnette, 1981; Laemmli, 1970). Briefly, SDS-PAGE-separated proteins were transferred into polyvinylidene difluoride (PVDF) membranes and probed with antibodies after being blocked with 5% non-fat dry milk in TBS-T (140 mM NaCl, 25 mM Tris- HCl, pH 7.4, 0.1% Tween- 20). Primary and secondary antibodies were applied at dilutions of 1:1000 and 1:10,000, respectively. HtrA and CagA were detected using primary rabbit polyclonal α-HtrA (Zawilak-Pawlik et al., 2019) and α-CagA (#HPP-5003-9, Austral Biologicals, San Ramon, CA, USA), respectively. Primary rabbit polyclonal α-FlaA (Boehm et al., 2011) and mouse monoclonal α-GAPDH (#sc-47724, Santa Cruz, Heidelberg, Germany) were used for the loading controls. Secondary goat α-mouse (#31446, Invitrogen, Darmstadt, Germany) or α-rabbit (#31460, Invitrogen, Darmstadt, Germany) antibodies conjugated with horseradish peroxidase were used as secondary antibodies for the following detection by the ECL Plus chemiluminescence Western Blot kit (GE Healthcare Life Sciences, Munich, Germany) as described (Moonens et al., 2018).

### 4.3 MKN-28 infection with *H. pylori*

For infection *H. pylori* wt and *H. pylori* Δ*htrA* were grown for 2 days at 37 °C in anaerobic chambers containing a CampyGen gas mix (Oxoid, Wesel/Germany) (Wiedemann et al., 2012). *H. pylori* was harvested and resuspended in phosphate buffered saline (PBS, pH 7.4) using sterile cotton swabs (Carl Roth, Karlsruhe/Germany). The bacterial concentration was measured in a spectrophotometer as optical density (OD) at 600 nm (Eppendorf, Hamburg/Germany). Apical marker expression such as microvilli and tight junction formation were routinely checked as described (Tegtmeyer et al., 2017b). Infections were performed from the apical side at a multiplicity of infection (MOI) of 100 as described (Kim et al., 2013), unless indicated otherwise. All infection assays were repeated four times.

### 4.4 RNA-seq and differential gene expression

After 2 or 6 h of infection, MKN-28 cells were harvested. Total RNA was extracted using the RNeasy Mini kit (Qiagen) as described (Heimesaat et al., 2014). Illumina sequencing libraries were constructed according to the manufacturer's instructions, and were subjected to single-end sequencing (101 bp) on a HiSeq-2500 platform (Illumina, San Diego, CA). Quality filtering was performed using cutadapt v. 1.9.1 (Martin, 2011). The reads were mapped against the human reference genome (Ensembl GRCh37, release 87) using STAR aligner v. 2.5.2 b (Dobin et al., 2013), and a STAR genome directory created by supplying the Ensembl gtf annotation file (release 87) for GRCh37. Read counts per gene were obtained using featureCounts v. 1.5.1 (Liao et al., 2014) and the Ensembl gtf annotation file.

The subsequent analyses were performed using R version 4.1.1 (Team, 2020) in the RStudio platform (Team, 2021). In particular, differential expression analysis was performed with the DESeq2 package v.1.32.0 (Love et al., 2014). DESeq2 performs by default shrinkage of fold changes and “independent filtering”, i.e. it finds a threshold on the mean normalized counts that optimizes the number of differentially expressed genes (DEGs). The list of the most significant DEGs with a log_2_FoldChange threshold of <1 or >1 are presented in Table S2. A set of representative genes showing the expression levels of all 20 samples along with their computed log_2_ fold changes are presented in Fig. S5 to illustrate the data quality and signal differences between different groups. Volcano plots representing the most significant DEGs were constructed using EnhancedVolcano, an R package version 1.10.0 (Blighe et al., 2021). Venn diagrams were drawn using ggvenn, an R package version 0.1.9, available from https://github.com/yanlinlin82/ggvenn.

### 4.5 Pathway analysis

The functional enrichment analysis was performed in RStudio by the Gene Ontology (GO) overrepresentation analysis using ClusterProfiler, an R package version 4.0.5 (Yu et al., 2012). The grch37 table with human annotations based on genome assembly GRCH37 from Ensembl was loaded from the annotables library (an R package version 0.1.91) and further used for conversion of gene IDs. The p values in GO enrichment analysis were adjusted using the Benjamini-Hochberg (BH) false discovery rate. To analyze the pathways enriched with DEGs we used KEGG (Kyoto Encyclopedia of Genes and Genomes) annotation data supported in ClusterProfiler. To visualize selected pathways, the Pathview, an R package (version 1.32.0) was used (Luo and Brouwer, 2013). Alternatively, the DEGs were analyzed using Ingenuity Pathway Analysis (IPA) Tool (Kramer et al., 2014), in particular, to determine the upstream regulators which were significantly affected by *H. pylori* infection. Log_2_ fold change values and corresponding identifiers of DEGs were used as an input data for IPA. Upstream regulator analysis was performed to predict the top significantly activated (Z-score ≥ 2) and inhibited (Z-score ≤ −2) upstream regulators corresponding to the input data, considering the direction of change, i.e. up- or downregulation. The overlap p values reflect statistically significant overlap between the dataset genes and the genes that are regulated by an upstream regulator, and were calculated using Fisher's Exact Test. The upstream regulators with the false discovery rate (p value) < 0.05 and the overlap p value < 0.05 were considered significant.

### 4.6 RT-qPCR

Expression levels of *ZFP36*, *TNF*, *NFKBIA*, *IL8*, *DKK1,* and *DOCK8* were assessed by quantitative reverse transcription PCR (RT-qPCR) in quadruplicates using primers listed in Table S11. Expression of *GAPDH* served as an internal control. The expression levels were analyzed using SYBR Green PCR master mix in the iCycler/MyiQ Real Time PCR detection system (Bio-Rad, USA) as described (Pachathundikandi et al., 2018). The obtained cycle threshold (CT) values were used to quantify relative expression levels as ΔCT (CT_reference gene_ – CT_gene of interest_) and ΔΔCT (ΔCT_infection_ – mean ΔCT_mock_) values. The difference between infection groups was analyzed using two-tailed *t*-test of the corresponding ΔΔCT values. The expression levels were finally presented as fold changes (2^ΔΔCT).

### 4.7 Immunofluorescence microscopy

To assess the amount and distribution of *H. pylori* over gastric cells, MKN-28 cells were grown on glass coverslips in 12-well plates until the formation of proper monolayers. After infection with *H. pylori* wt or *H. pylori* Δ*htrA* at MOI of 25 for 2 or 6 h, cells were fixed with cold methanol for 10 min and immunostained using mouse FITC-conjugated α-occludin (#331511, Invitrogen, Waltham, MA, USA). Primary rabbit α-*H. pylori* (Dako, Glostrup, Denmark) and secondary α-rabbit-AlexaFluor 633 (#A-21070, Invitrogen, Darmstadt, Germany) were used to detect *H. pylori* cells. Nuclei were counterstained using 1 ​μg/mL DAPI (4′-6-diamidino-2-phenylindole dihydrochloride). The images were acquired using confocal laser scanning microscope Leica Stellaris 8 (Leica Microsystems, Wetzlar, Germany) at the Optical Imaging Centre Erlangen (OICE, Erlangen, Germany). LAS AF computer software (Leica Microsystems, Wetzlar, Germany) was used to visualize the obtained data. To analyze the distribution of bacterial cells adhered to MKN-28 monolayer, the number of bacteria in the cell junction area was divided by the number of bacteria outside of the cell junctions area. Welch's ANOVA combined with Holm-Sidak's post-test was used to define the differences between the groups with p ​≤ ​0.05 (∗) considered to be significant, and with p ​> ​0.05 to be non-significant (n.s.).

### 4.8 Apoptosis assay

MKN-28 cells were grown in 12-well plates until the formation of proper monolayers before being infected as described above. After infection, cells were washed with HEPES-buffer (10 mM HEPES, 140 mM NaCl, 2.5 mM CaCl_2_) and additionally incubated for 30 ​min in HEPES-buffer containing 1 ​μg/mL annexin V conjugated with fluorescein isothiocyanate (FITC) and 1 ​μg/mL propidium iodide (PI). Subsequently, the cells were washed with HEPES-buffer and analyzed under a DMI4000B epifluorescence microscope (Leica Microsystems, Wetzlar, Germany). Cells stained positive for either annexin V-FITC or PI were considered as undergoing apoptosis/necrosis and were presented as percentage out of all counted cells as described (Sharafutdinov et al., 2022). Welch's ANOVA combined with Holm-Sidak's post-test was used to define the differences between the groups with p ​≤ ​0.05 (∗) considered to be significant.

## 5 Statistics

The statistical analysis details are explained after every method description where appropriate. The Welch's ANOVA and two-tailed unpaired *t*-test statistical analyses were performed using GraphPad Prism statistical software version 8.0 (GraphPad Software, United States). Statistical significance was defined as p ​≤ ​0.05 (∗) or otherwise non-significant (n.s.). Graphs in Fig. 1B, **C**, **4B**, **5D**, and **5E** are presented as mean values ± standard deviation (SD). In addition, Fig. 4B shows the individual values as black dots.

## Data availability

The data discussed in this publication have been deposited in NCBI's Gene Expression Omnibus (Edgar et al., 2002) and are accessible through GEO Series accession number GSE202165 (https://www.ncbi.nlm.nih.gov/geo/query/acc.cgi?acc=GSE202165).

## Declaration of competing interest

The authors declare no conflict of interest.
