# Supplementary material for: Early and late genome-wide gastric epithelial transcriptome response during infection with the human carcinogen Helicobacterpylori
Source: Cell Insight. 2022 May 25;1(3):100032. doi: 10.1016/j.cellin.2022.100032 (PMC10120309; doi:10.1016/j.cellin.2022.100032)
Supplement: Multimedia component 1 [file mmc1.docx]

**
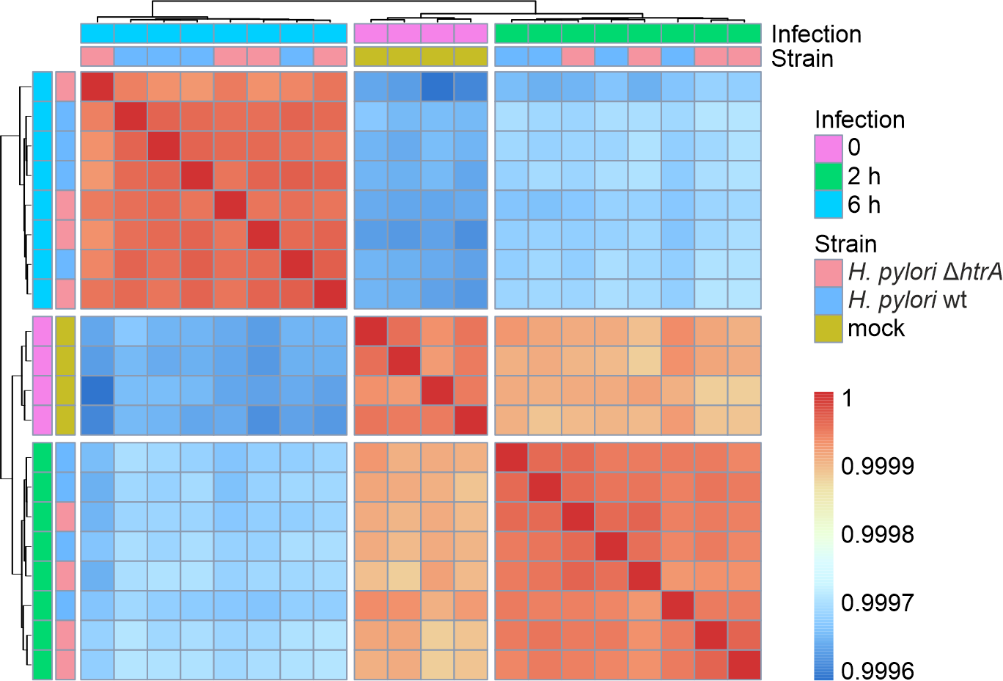
**

**Figure S1.** Sample-to-sample heatmap of rlog transformed read counts across all replicates in uninfected MKN-28 cells (mock), MKN-28 infected with *H. pylori* wt or Δ*htrA* mutant bacteria for 2 or 6 hours.

**
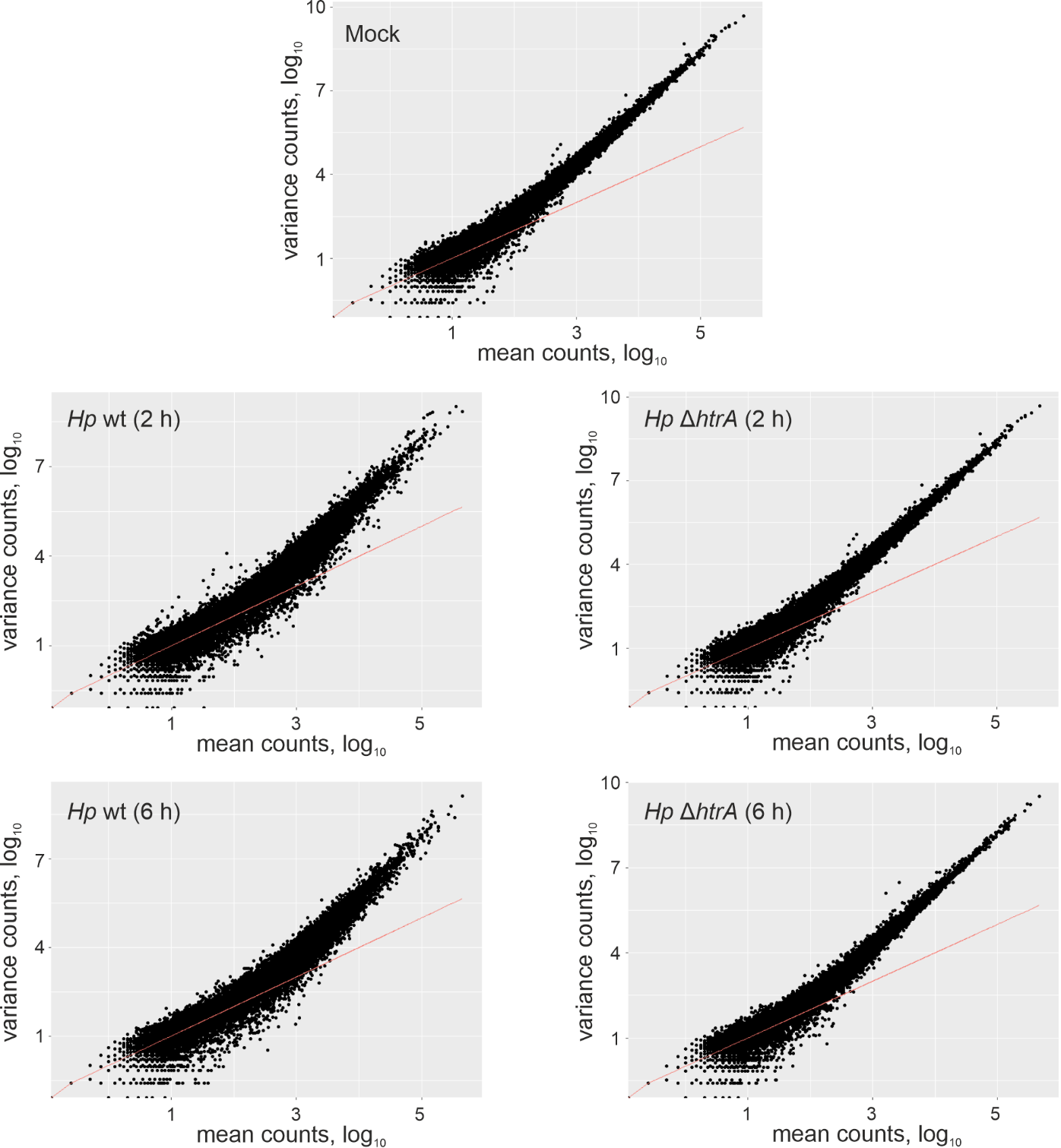
**

**Figure S2.** Scatter plots of mean versus the variance of raw counts across the replicates per condition.

**
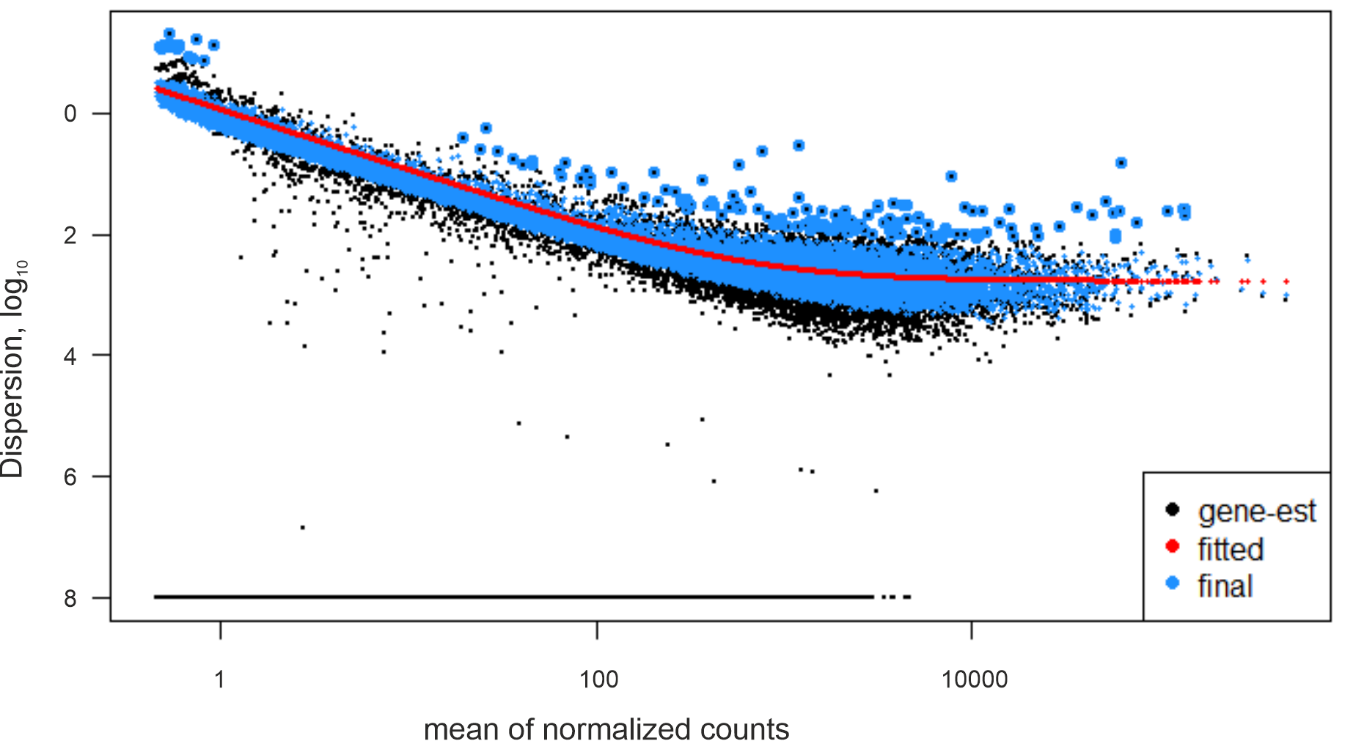
**

**Figure S3.** The per-gene dispersion estimates along with the fitted mean-dispersion relationship. Black dots show dispersion estimates for each gene as obtained by considering the information from each gene separately. Fitted estimates reflecting the dependence of dispersions on the mean are shown by the red line. Blue dots display the final dispersion estimates shrunk from the gene-wise estimates towards the fitted estimates. Blue circles indicate genes with high gene-wise dispersion estimates (outliers).

**
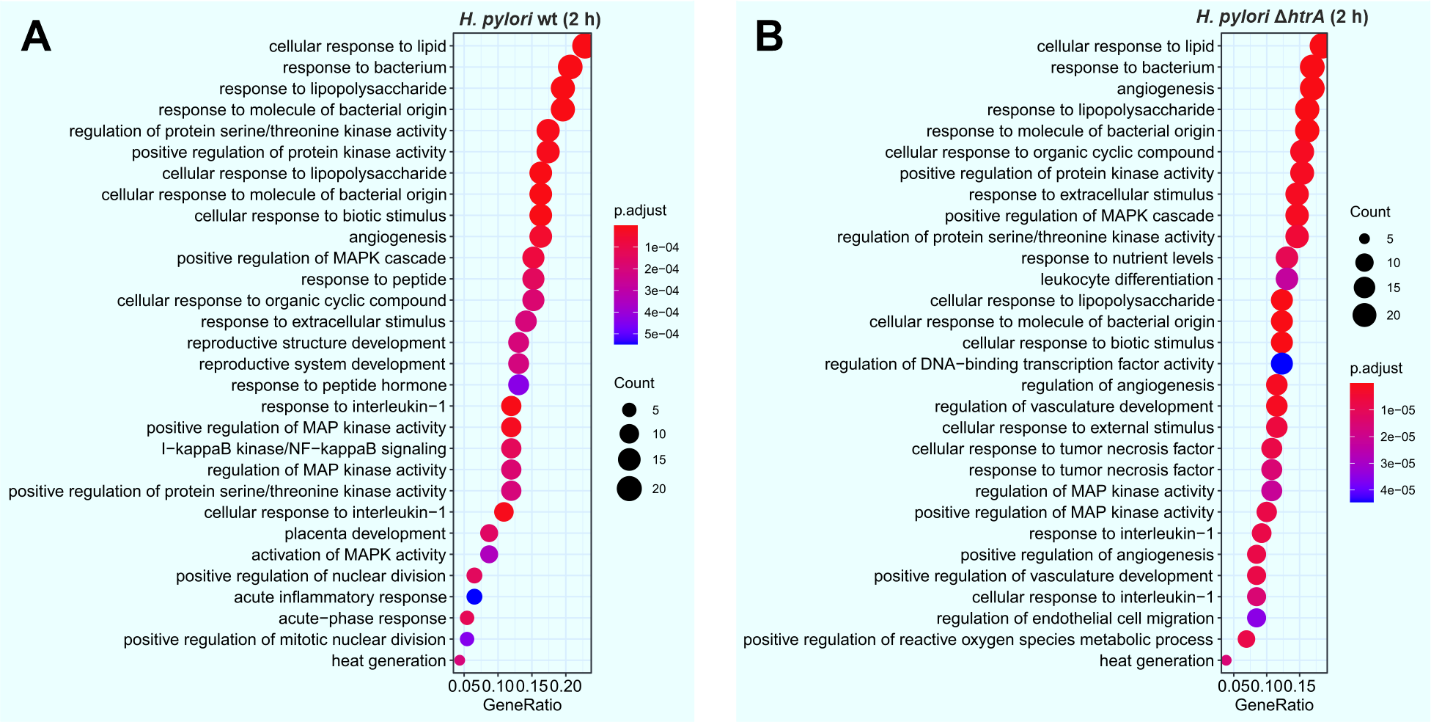
**

**Figure S4.** Gene Ontology (GO) term enrichment of biological pathways affected in MKN-28 cells after 2-hour infection with *H. pylori* wt (**A**) or Δ*htrA* mutant (**B**). GO terms were ordered according to their gene ratio values which represent the number of DEGs connected to the corresponding GO term, divided by the total number of DEGs. The size of the dots represents the number of DEGs (Count) associated with the GO term, the color represents the adjusted p-value (p.adjust).

**
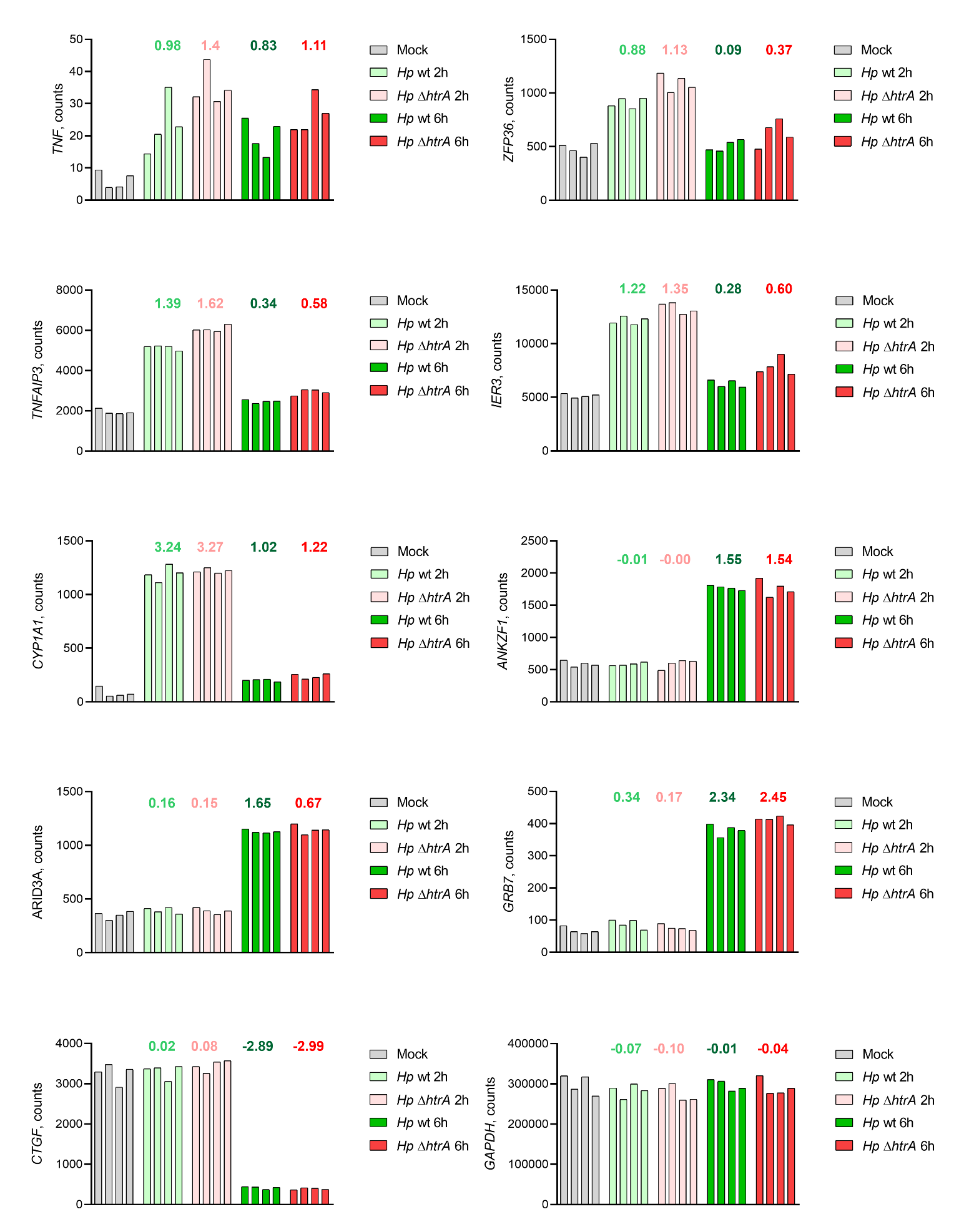
**

**Figure S5.** Read counts of representative MKN-28 genes after infection with *H. pylori* wt or Δ*htrA* mutant for 2 or 6 hours. The numbers above the bars represent the corresponding log_2_ fold changes calculated by the DESeq2 analysis.

**Table S1.** Basic quality control of raw reads after RNA-seq.

| **Sample** | **Raw reads**  **numreads** | **Filtered reads (after quality, adapter trimming)** | | **Mapping** | | **Read counts over exons** | |
| --- | --- | --- | --- | --- | --- | --- | --- |
|  |  | **numreads** | **% of raw reads** | **num uniquely mapped** | **% of filtered** | **num counted** | **% of raw reads** |
| mock1 | 40,692,631 | 40,628,500 | 99.84 | 36,426,111 | 89.66 | 33,979,420 | 83.50 |
| mock2 | 48,281,405 | 48,181,186 | 99.79 | 43,123,159 | 89.50 | 40,176,938 | 83.21 |
| mock3 | 46,698,915 | 46,593,303 | 99.77 | 41,619,017 | 89.32 | 38,695,556 | 82.86 |
| mock4 | 44,244,576 | 44,175,141 | 99.84 | 39,393,117 | 89.17 | 36,549,539 | 82.61 |
| H1_2h_1 | 46,288,950 | 46,202,574 | 99.81 | 41,551,025 | 89.93 | 38,816,315 | 83.86 |
| H1_2h_2 | 48,177,906 | 48,086,905 | 99.81 | 43,390,503 | 90.23 | 40,414,381 | 83.89 |
| H1_2h_3 | 52,745,181 | 52,660,965 | 99.84 | 47,975,810 | 91.10 | 44,577,410 | 84.51 |
| H1_2h_4 | 51,496,851 | 51,441,116 | 99.89 | 46,299,856 | 90.01 | 43,104,217 | 83.70 |
| H2_2h_1 | 56,553,274 | 56,376,713 | 99.69 | 51,211,545 | 90.84 | 47,774,122 | 84.48 |
| H2_2h_2 | 40,969,851 | 40,909,373 | 99.85 | 36,881,558 | 90.15 | 34,391,814 | 83.94 |
| H2_2h_3 | 50,558,028 | 50,493,869 | 99.87 | 45,520,842 | 90.15 | 42,187,333 | 83.44 |
| H2_2h_4 | 44,372,984 | 44,275,012 | 99.78 | 39,893,349 | 90.10 | 36,977,425 | 83.33 |
| H1_6h_1 | 50,728,005 | 50,651,327 | 99.85 | 46,014,706 | 90.85 | 42,921,377 | 84.61 |
| H1_6h_2 | 43,346,783 | 43,298,124 | 99.89 | 39,439,631 | 91.09 | 36,781,698 | 84.85 |
| H1_6h_3 | 46,142,802 | 46,086,937 | 99.88 | 41,943,072 | 91.01 | 38,964,052 | 84.44 |
| H1_6h_4 | 47,361,834 | 47,273,589 | 99.81 | 43,031,541 | 91.03 | 39,980,814 | 84.42 |
| H2_6h_1 | 49,690,146 | 49,629,781 | 99.88 | 45,281,721 | 91.24 | 42,006,868 | 84.54 |
| H2_6h_2 | 40,852,031 | 40,792,790 | 99.85 | 37,220,866 | 91.24 | 34,644,420 | 84.80 |
| H2_6h_3 | 53,682,807 | 53,582,268 | 99.81 | 48,923,464 | 91.31 | 45,585,542 | 84.92 |
| H2_6h_4 | 51,112,725 | 51,046,297 | 99.87 | 46,558,581 | 91.21 | 43,350,848 | 84.81 |

**Table S3.** Top 20 biological processes with the most affected genes in MKN-28 after 2-hour infection with *H. pylori* wt, as determined by overrepresentation analysis. Gene ratio is the number of genes involved in a biological process over all significant DEGs (adjusted p value < 0.05, log_2_FoldChange < -0.58 or log_2_FoldChange > 0.58). P_adj_ – adjusted p value.

| **ID** | **Biological process** | **Gene ratio** | **P_adj_** | **Affected genes** |
| --- | --- | --- | --- | --- |
| GO:0071222 | cellular response to lipopolysaccharide | 15/92 | 3.78171E-11 | IL24, IL1A, IL1B, IRAK2, WNT5A, IL8, CXCL3, CXCL2, TNFAIP3, SASH1, ANKRD1, NFKBIA, CEBPB, ICAM1, ZFP36 |
| GO:0032496 | response to lipopolysaccharide | 18/92 | 3.78171E-11 | IL24, IL1A, IL1B, IRAK2, WNT5A, IL8, CXCL3, CXCL2, TNFAIP3, SASH1, SOD2, ANKRD1, NFKBIA, CYP1A1, TNFRSF11A, CEBPB, ICAM1, ZFP36 |
| GO:0071219 | cellular response to molecule of bacterial origin | 15/92 | 4.06368E-11 | IL24, IL1A, IL1B, IRAK2, WNT5A, IL8, CXCL3, CXCL2, TNFAIP3, SASH1, ANKRD1, NFKBIA, CEBPB, ICAM1, ZFP36 |
| GO:0002237 | response to molecule of bacterial origin | 18/92 | 4.06368E-11 | IL24, IL1A, IL1B, IRAK2, WNT5A, IL8, CXCL3, CXCL2, TNFAIP3, SASH1, SOD2, ANKRD1, NFKBIA, CYP1A1, TNFRSF11A, CEBPB, ICAM1, ZFP36 |
| GO:0071216 | cellular response to biotic stimulus | 15/92 | 1.7308E-10 | IL24, IL1A, IL1B, IRAK2, WNT5A, IL8, CXCL3, CXCL2, TNFAIP3, SASH1, ANKRD1, NFKBIA, CEBPB, ICAM1, ZFP36 |
| GO:0071396 | cellular response to lipid | 21/92 | 3.7802E-10 | IL24, WNT9A, IL1A, IL1B, IRAK2, WNT5A, IL8, CXCL3, CXCL2, TNFAIP3, SASH1, INSIG1, FBXO32, LPAR1, ANKRD1, NFKBIA, CEBPB, ICAM1, ZFP36, MN1, WNT7B |
| GO:0009617 | response to bacterium | 19/92 | 8.75731E-09 | IL24, IL1A, IL1B, IRAK2, WNT5A, IL8, CXCL3, CXCL2, TNFAIP3, SASH1, SOD2, ANKRD1, NFKBIA, CYP1A1, TNFRSF11A, CEBPB, ICAM1, ZFP36, BCL3 |
| GO:0070555 | response to interleukin-1 | 11/92 | 3.92119E-06 | IL1A, IL1B, IL1RN, IRAK2, IL8, EGR1, ANKRD1, NFKBIA, TNFRSF11A, CEBPB, ICAM1 |
| GO:0071900 | regulation of protein serine/threonine kinase activity | 16/92 | 6.59472E-06 | SESN2, TGFA, IL1B, IRAK2, WNT5A, EPGN, CCNG2, CCNJL, CDKN1A, TNFAIP3, SASH1, LPAR1, ERCC6, DKK1, TNFRSF11A, GDF15 |
| GO:0045860 | positive regulation of protein kinase activity | 16/92 | 9.86517E-06 | TGFA, IL1B, IRAK2, WNT5A, EPGN, EREG, EGR1, CDKN1A, SASH1, LPAR1, ERCC6, DKK1, TNFRSF11A, SDC4, GDF15, CARD10 |
| GO:0071347 | cellular response to interleukin-1 | 10/92 | 9.90313E-06 | IL1A, IL1B, IL1RN, IRAK2, IL8, EGR1, ANKRD1, NFKBIA, CEBPB, ICAM1 |
| GO:0043406 | positive regulation of MAP kinase activity | 11/92 | 1.71941E-05 | TGFA, IL1B, IRAK2, WNT5A, EPGN, SASH1, LPAR1, ERCC6, DKK1, TNFRSF11A, GDF15 |
| GO:0001525 | angiogenesis | 15/92 | 3.99105E-05 | PTGS2, CYP1B1, IL1A, IL1B, WNT5A, IL8, EPGN, EREG, TNFAIP3, SASH1, CEMIP2, E2F7, TNFAIP2, FOXC2, CARD10 |
| GO:0043410 | positive regulation of MAPK cascade | 14/92 | 7.18683E-05 | TGFA, IL1B, IRAK2, WNT5A, EPGN, SASH1, LPAR1, TRAF1, ERCC6, DKK1, TNFRSF11A, ICAM1, GDF15, WNT7B |
| GO:0006953 | acute-phase response | 5/92 | 0.000119372 | PTGS2, IL1A, IL1B, TNFRSF11A, CEBPB |
| GO:0007249 | I-kappaB kinase/NF-kappaB signaling | 11/92 | 0.000128855 | REL, IL1B, IRAK2, WNT5A, TNFAIP3, LPAR1, TRAF1, BIRC3, NFKBIA, SECTM1, BCL3 |
| GO:0051785 | positive regulation of nuclear division | 6/92 | 0.00013669 | TGFA, IL1A, IL1B, WNT5A, EPGN, EREG |
| GO:1901652 | response to peptide | 14/92 | 0.00013669 | SESN2, BTG2, IL1B, NR4A2, EREG, EGR1, STC2, TNFAIP3, IGFBP1, INSIG1, NFKBIA, FOXC2, ICAM1, GDF15 |
| GO:0001890 | placenta development | 8/92 | 0.000147875 | LHX4, STOX2, STC2, FOSL1, E2F7, CEBPB, JUNB, WNT7B |
| GO:0043405 | regulation of MAP kinase activity | 11/92 | 0.000175675 | TGFA, IL1B, IRAK2, WNT5A, EPGN, SASH1, LPAR1, ERCC6, DKK1, TNFRSF11A, GDF15 |
| GO:0071407 | cellular response to organic cyclic compound | 14/92 | 0.000175675 | HTR1D, CYP1B1, IL1B, TIPARP, EGR1, INSIG1, FBXO32, ANKRD1, CHRM1, CYP1A1, CEBPB, ICAM1, ZFP36, MN1 |
| GO:0031649 | heat generation | 4/92 | 0.000197158 | PTGS2, IL1A, IL1B, TNFRSF11A |
| GO:0009991 | response to extracellular stimulus | 13/92 | 0.000197158 | SESN2, ATF3, NR4A2, STC2, CDKN1A, SOD2, SLC22A3, FOSL1, CYP1A1, ICAM1, GDF15, ZFP36, MN1 |
| GO:0071902 | positive regulation of protein serine/threonine kinase activity | 11/92 | 0.000197158 | TGFA, IL1B, IRAK2, WNT5A, EPGN, SASH1, LPAR1, ERCC6, DKK1, TNFRSF11A, GDF15 |
| GO:0048608 | reproductive structure development | 12/92 | 0.000197158 | LHX4, WNT5A, TIPARP, EREG, STOX2, STC2, FOSL1, E2F7, CEBPB, ICAM1, JUNB, WNT7B |
| GO:0061458 | reproductive system development | 12/92 | 0.000208282 | LHX4, WNT5A, TIPARP, EREG, STOX2, STC2, FOSL1, E2F7, CEBPB, ICAM1, JUNB, WNT7B |
| GO:0000187 | activation of MAPK activity | 8/92 | 0.000342026 | TGFA, IL1B, IRAK2, WNT5A, EPGN, LPAR1, ERCC6, GDF15 |
| GO:0043434 | response to peptide hormone | 12/92 | 0.000432002 | SESN2, BTG2, IL1B, NR4A2, EREG, EGR1, STC2, IGFBP1, INSIG1, FOXC2, ICAM1, GDF15 |
| GO:0045840 | positive regulation of mitotic nuclear division | 5/92 | 0.000442229 | TGFA, IL1A, IL1B, EPGN, EREG |
| GO:0002526 | acute inflammatory response | 6/92 | 0.000548716 | PTGS2, IL1A, IL1B, TNFRSF11A, CEBPB, ICAM1 |

**Table S4.** Top 20 biological processes with the most affected genes in MKN-28 after 2-hour infection with *H. pylori* Δ*htrA*, as determined by overrepresentation analysis.

| **ID** | **Biological process** | **Gene ratio** | **P_adj_** | **Affected genes** |
| --- | --- | --- | --- | --- |
| GO:0032496 | response to lipopolysaccharide | 21/130 | 3.47434E-11 | ZC3H12A, JUN, IL24, IL1A, IL1B, IRAK2, WNT5A, IL8, CXCL3, CXCL2, SPARC, TNF, TNFAIP3, SASH1, SOD2, ANKRD1, NFKBIA, CYP1A1, TNFRSF11A, ICAM1, ZFP36 |
| GO:0002237 | response to molecule of bacterial origin | 21/130 | 4.19047E-11 | ZC3H12A, JUN, IL24, IL1A, IL1B, IRAK2, WNT5A, IL8, CXCL3, CXCL2, SPARC, TNF, TNFAIP3, SASH1, SOD2, ANKRD1, NFKBIA, CYP1A1, TNFRSF11A, ICAM1, ZFP36 |
| GO:0071222 | cellular response to lipopolysaccharide | 16/130 | 3.60787E-10 | ZC3H12A, IL24, IL1A, IL1B, IRAK2, WNT5A, IL8, CXCL3, CXCL2, TNF, TNFAIP3, SASH1, ANKRD1, NFKBIA, ICAM1, ZFP36 |
| GO:0071219 | cellular response to molecule of bacterial origin | 16/130 | 5.16615E-10 | ZC3H12A, IL24, IL1A, IL1B, IRAK2, WNT5A, IL8, CXCL3, CXCL2, TNF, TNFAIP3, SASH1, ANKRD1, NFKBIA, ICAM1, ZFP36 |
| GO:0071396 | cellular response to lipid | 24/130 | 1.74123E-09 | ZC3H12A, IL24, WNT9A, IL1A, IL1B, IRAK2, WNT5A, IL8, CXCL3, CXCL2, TNF, PIM1, TNFAIP3, SASH1, INSIG1, FBXO32, LPAR1, ZMIZ1, ANKRD1, NFKBIA, ICAM1, ZFP36, MN1, WNT7B |
| GO:0071216 | cellular response to biotic stimulus | 16/130 | 2.44369E-09 | ZC3H12A, IL24, IL1A, IL1B, IRAK2, WNT5A, IL8, CXCL3, CXCL2, TNF, TNFAIP3, SASH1, ANKRD1, NFKBIA, ICAM1, ZFP36 |
| GO:0009617 | response to bacterium | 22/130 | 1.78239E-08 | ZC3H12A, JUN, IL24, IL1A, IL1B, IRAK2, WNT5A, IL8, CXCL3, CXCL2, SPARC, TNF, TNFAIP3, SASH1, SOD2, ANKRD1, NFKBIA, CYP1A1, TNFRSF11A, ICAM1, ZFP36, BCL3 |
| GO:0001525 | angiogenesis | 22/130 | 5.2836E-08 | ZC3H12A, JUN, PTGS2, CYP1B1, IL1A, IL1B, NFE2L2, WNT5A, IL8, EPGN, EREG, SPARC, TNF, TNFAIP3, SASH1, HIPK2, CEMIP2, E2F7, TNFAIP2, THBS1, FOXC2, CARD10 |
| GO:0009991 | response to extracellular stimulus | 19/130 | 1.50645E-06 | SESN2, ZC3H12A, JUN, NUAK2, ATF3, NR4A2, NFE2L2, SPARC, STC2, CDKN1A, PIM1, SOD2, SLC22A3, FOSL1, CYP1A1, ICAM1, GDF15, ZFP36, MN1 |
| GO:0045765 | regulation of angiogenesis | 15/130 | 1.50645E-06 | ZC3H12A, CYP1B1, IL1A, IL1B, NFE2L2, WNT5A, IL8, SPARC, TNF, TNFAIP3, SASH1, HIPK2, CEMIP2, THBS1, FOXC2 |
| GO:1901342 | regulation of vasculature development | 15/130 | 1.536E-06 | ZC3H12A, CYP1B1, IL1A, IL1B, NFE2L2, WNT5A, IL8, SPARC, TNF, TNFAIP3, SASH1, HIPK2, CEMIP2, THBS1, FOXC2 |
| GO:0043410 | positive regulation of MAPK cascade | 19/130 | 1.536E-06 | ZC3H12A, TGFA, IL1B, IRAK2, WNT5A, EPGN, TNF, SASH1, HIPK2, LPAR1, TRAF1, ERCC6, DKK1, THBS1, TNFRSF11A, ICAM1, GDF15, LIF, WNT7B |
| GO:0071407 | cellular response to organic cyclic compound | 20/130 | 1.536E-06 | HTR6, HTR1D, ZC3H12A, CYP1B1, IL1B, GLI2, TIPARP, EGR1, TNF, PIM1, INSIG1, FBXO32, ZMIZ1, ANKRD1, CHRM1, SSH1, CYP1A1, ICAM1, ZFP36, MN1 |
| GO:0045860 | positive regulation of protein kinase activity | 20/130 | 1.536E-06 | TGFA, IL1B, IRAK2, WNT5A, EPGN, EREG, EGR1, TNF, CDKN1A, SASH1, PILRB, LPAR1, MAP3K8, ERCC6, DKK1, THBS1, TNFRSF11A, SDC4, GDF15, CARD10 |
| GO:0071900 | regulation of protein serine/threonine kinase activity | 19/130 | 4.505E-06 | SESN2, TGFA, IL1B, IRAK2, WNT5A, EPGN, CCNG2, SPRY4, CCNJL, TNF, CDKN1A, TNFAIP3, SASH1, LPAR1, ERCC6, DKK1, THBS1, TNFRSF11A, GDF15 |
| GO:0071496 | cellular response to external stimulus | 15/130 | 5.93672E-06 | SESN2, ZC3H12A, JUN, NUAK2, ATF3, IL1B, NR4A2, NFE2L2, IRF1, CDKN1A, PIM1, ANKRD1, FOSL1, ICAM1, MN1 |
| GO:0043406 | positive regulation of MAP kinase activity | 13/130 | 7.35793E-06 | TGFA, IL1B, IRAK2, WNT5A, EPGN, TNF, SASH1, LPAR1, ERCC6, DKK1, THBS1, TNFRSF11A, GDF15 |
| GO:0071356 | cellular response to tumor necrosis factor | 14/130 | 7.35793E-06 | ZC3H12A, NFE2L2, IL8, TNF, TNFAIP3, TRAF1, ANKRD1, KCNJ11, BIRC3, NFKBIA, THBS1, TNFRSF11A, ICAM1, ZFP36 |
| GO:0045766 | positive regulation of angiogenesis | 11/130 | 7.35793E-06 | ZC3H12A, CYP1B1, IL1A, IL1B, NFE2L2, WNT5A, IL8, SASH1, HIPK2, THBS1, FOXC2 |
| GO:1904018 | positive regulation of vasculature development | 11/130 | 7.35793E-06 | ZC3H12A, CYP1B1, IL1A, IL1B, NFE2L2, WNT5A, IL8, SASH1, HIPK2, THBS1, FOXC2 |
| GO:0070555 | response to interleukin-1 | 12/130 | 7.35793E-06 | ZC3H12A, IL1A, IL1B, IL1RN, IRAK2, IL8, EGR1, MAP3K8, ANKRD1, NFKBIA, TNFRSF11A, ICAM1 |
| GO:2000379 | positive regulation of reactive oxygen species metabolic process | 9/130 | 7.45169E-06 | ZC3H12A, PTGS2, IL1B, NFE2L2, TNF, CDKN1A, SOD2, THBS1, ICAM1 |
| GO:0031667 | response to nutrient levels | 17/130 | 9.55488E-06 | SESN2, ZC3H12A, JUN, NUAK2, ATF3, NFE2L2, SPARC, STC2, CDKN1A, PIM1, SOD2, SLC22A3, CYP1A1, ICAM1, GDF15, ZFP36, MN1 |
| GO:0071347 | cellular response to interleukin-1 | 11/130 | 1.49531E-05 | ZC3H12A, IL1A, IL1B, IL1RN, IRAK2, IL8, EGR1, MAP3K8, ANKRD1, NFKBIA, ICAM1 |
| GO:0034612 | response to tumor necrosis factor | 14/130 | 1.49531E-05 | ZC3H12A, NFE2L2, IL8, TNF, TNFAIP3, TRAF1, ANKRD1, KCNJ11, BIRC3, NFKBIA, THBS1, TNFRSF11A, ICAM1, ZFP36 |
| GO:0031649 | heat generation | 5/130 | 1.53019E-05 | PTGS2, IL1A, IL1B, TNF, TNFRSF11A |
| GO:0043405 | regulation of MAP kinase activity | 14/130 | 2.12508E-05 | TGFA, IL1B, IRAK2, WNT5A, EPGN, SPRY4, TNF, SASH1, LPAR1, ERCC6, DKK1, THBS1, TNFRSF11A, GDF15 |
| GO:0002521 | leukocyte differentiation | 17/130 | 2.23154E-05 | RUNX3, ZC3H12A, JUN, IL1A, IL1B, GLI2, BCL6, IRF1, EGR1, TNF, PRDM1, ZMIZ1, LRRK1, TNFRSF11A, JUNB, BCL3, LIF |
| GO:0010594 | regulation of endothelial cell migration | 11/130 | 3.485E-05 | ZC3H12A, PTGS2, ATOH8, NFE2L2, WNT5A, SPARC, TNF, SASH1, THBS1, FOXC2, CARD10 |
| GO:0051090 | regulation of DNA-binding transcription factor activity | 16/130 | 4.45932E-05 | ZC3H12A, JUN, CYP1B1, IL1B, IRAK2, WNT5A, TNF, PIM1, NFKBIE, TNFAIP3, HIPK2, TRAF1, FOSL1, NFKBIA, TNFRSF11A, ICAM1 |

**Table S5.** Biological processes with the most affected genes in MKN-28 after 6-hour infection with *H. pylori* wt, as determined by overrepresentation analysis.

| **ID** | **Biological process** | **Gene ratio** | **P_adj_** | **Affected genes** |
| --- | --- | --- | --- | --- |
| GO:0048646 | anatomical structure formation involved in morphogenesis | 135  /1427 | 0.011430316 | CASP9/GRHL3/PHACTR4/ADGRB2/HEYL/MPL/GADD45A/CCN1/F3/SARS1/EFNA3/GLUL/PTGS2/CRB1/IGFN1/CYP1B1/EPAS1/HK2/EIF2AK3/DUSP2/SEMA4C/FHL2/EPB41L5/GLI2/CLASP1/COL5A2/FZD7/BMPR2/FN1/ACKR3/TCTA/GATA2/BCL6/TP63/FGFBP1/IL8/NDNF/SPRY1/GAB1/EDNRA/TPPP/TERT/PLK2/ERAP1/FBN2/SPINK5/PDGFRB/TCOF1/SPARC/DUSP1/POU5F1/TNF/FKBPL/SRF/RSPO3/CCN2/CITED2/THBS2/PDGFA/C1GALT1/HOXA5/COBL/HSPB1/EPHB4/MED12/PTK2B/SULF1/HEY1/STK3/EXT1/SLC1A1/AGTPBP1/PTCH1/KLF4/C5/ARHGAP22/DKK1/UNC5B/LDB3/ANKRD1/NFKB2/SUFU/SH3PXD2A/DUSP5/KNDC1/E2F8/FJX1/RELT/IL18/NECTIN1/ST14/WNT10B/KRT8/CSRP2/E2F7/ELK3/APAF1/STARD13/RGCC/PRKD1/FMN1/THBS1/RORA/SMAD3/CSPG4/MINAR1/BMERB1/ADGRG1/MMP15/CDH5/KDM6B/HES7/EPN2/KAT2A/MAP3K3/PRKCA/ITGB4/UNC13D/MAFB/NFATC2/KLF2/GDF15/HSPB6/BCL3/HIF3A/PRKD2/C5AR1/MBOAT7/ZNF304/PLA2G3/CCDC134/TTLL1/PPARA/ADM2/ADAMTS1 |
| GO:0030334 | regulation of cell migration | 114  /1427 | 0.020684763 | MIIP/NBL1/EDN2/GADD45A/CCN1/F3/S100A14/DDR2/GLUL/LAMC2/PTGS2/KIF14/DUSP10/CYP1B1/SLC8A1/SEMA4C/IL1R1/EPB41L5/CLASP1/MGAT5/BMPR2/ERBB4/FN1/DOCK10/ACKR3/FOXP1/PLXNA1/GATA2/PFN2/FGFBP1/IL8/GAB1/TERT/PLK2/F2RL1/ARSB/HBEGF/PDGFRB/SPARC/DUSP1/GCNT2/CARMIL1/TNF/AGER/SRF/LAMA2/SGK1/CITED2/AKAP12/AFDN/PDGFA/IGFBP3/HSPB1/DOCK4/TCAF2/GNRH1/PTK2B/FGFR1/SULF1/TRIB1/DOCK8/DDX58/ARHGEF39/SEMA4D/KLF4/C5/SCAI/HSPA5/NSMF/PBLD/VSIR/SORL1/ARHGDIB/ARID2/ZNF268/STARD13/RGCC/GPR183/PRKD1/FERMT2/THBS1/TTBK2/ZNF609/SMAD3/DNAJA4/SEMA4B/IGF1R/BMERB1/CORO1A/ADGRG1/CDH5/TRADD/RIPOR1/PLCG2/MEAK7/TMEM102/MAP2K3/GRB7/KRT16/MAP3K3/PRKCA/LAMA1/SERPINB3/INSR/CAMSAP3/CALR/SYDE1/PRKD2/C5AR1/MYADM/ZNF304/GTSE1/ADAMTS1/AIRE |
| GO:0051270 | regulation of cellular component movement | 127  /1427 | 0.021388043 | MIIP/NBL1/EDN2/GADD45A/CCN1/F3/S100A14/DDR2/GLUL/LAMC2/PTGS2/KIF14/DUSP10/CCSAP/CYP1B1/SLC8A1/SEMA4C/IL1R1/EPB41L5/CLASP1/MGAT5/BMPR2/ERBB4/FN1/DOCK10/ACKR3/FOXP1/PLXNA1/GATA2/PFN2/BCL6/DYNLT2B/FGFBP1/IL8/ANK2/GAB1/TERT/PLK2/F2RL1/ARSB/HBEGF/PDGFRB/SPARC/DUSP1/GCNT2/CARMIL1/TNF/AGER/SRF/LAMA2/SGK1/CITED2/AKAP12/AFDN/PDGFA/IGFBP3/HSPB1/SRI/DOCK4/TCAF2/GNRH1/PTK2B/FGFR1/SULF1/TRIB1/DOCK8/DDX58/ARHGEF39/SEMA4D/KLF4/C5/SCAI/HSPA5/NSMF/PBLD/VSIR/ZSWIM8/SORL1/ARHGDIB/ARID2/ERBB3/ZNF268/STARD13/RGCC/MYCBP2/GPR183/PRKD1/FERMT2/THBS1/TTBK2/ZNF609/SMAD3/DNAJA4/SEMA4B/IGF1R/BMERB1/CORO1A/ADGRG1/CDH5/TRADD/RIPOR1/PLCG2/DNAAF1/MEAK7/CLDN7/TMEM102/MAP2K3/GRB7/KRT16/TTLL6/MAP3K3/PRKCA/KCNJ2/LAMA1/SERPINB3/INSR/CAMSAP3/CALR/ZSWIM4/SYDE1/PRKD2/C5AR1/MYADM/ZNF304/GTSE1/ADAMTS1/AIRE |
| GO:0035295 | tube development | 127  /1427 | 0.021388043 | GRHL3/PHACTR4/ADGRB2/HEYL/EDN2/GADD45A/CCN1/F3/SARS1/EFNA3/GPR161/GLUL/PTGS2/CYP1B1/SOS1/PKDCC/EPAS1/LOXL3/HK2/EIF2AK3/SEMA4C/EPB41L5/GLI2/BMPR2/ABCA12/FN1/ACKR3/OXTR/FOXP1/GATA2/TP63/FGFBP1/IL8/NDNF/SPRY1/INTU/GAB1/EDNRA/TERT/ADAMTS16/PLK2/ERAP1/SPINK5/PDGFRB/SPARC/TNF/FKBPL/SPDEF/SRF/EPHA7/RSPO3/CCN2/CITED2/ESR1/THBS2/PDGFA/C1GALT1/AGR2/HOXA5/COBL/HSPB1/EPHB4/SLC4A2/MED12/PTK2B/EIF4EBP1/SULF1/HEY1/STK3/EXT1/SLC1A1/PTCH1/KLF4/C5/ARHGAP22/UNC5B/LZTS2/BTRC/SUFU/E2F8/KIF18A/LRP5/IL18/ST14/ARID2/E2F7/ELK3/APAF1/STARD13/RGCC/MIR17HG/PRKD1/ARG2/FMN1/THBS1/RORA/SMAD3/CYP1A1/CSPG4/MINAR1/ABCA3/ADGRG1/CDH5/DNAAF1/CRISPLD2/FOXL1/EPN2/FOXN1/KAT2A/BRIP1/MAP3K3/PRKCA/LAMA1/CBFA2T2/SRSF6/FSTL3/KLF2/RAB3A/HSPB6/HIF3A/PRKD2/C5AR1/ZNF304/LIF/CCDC134/ADM2/ADAMTS1 |
| GO:0008283 | cell population proliferation | 202  /1427 | 0.021388043 | PRKCZ/FBXO2/CAMK2N1/IFNLR1/RPS6KA1/AGO3/EDN2/MPL/CCN1/F3/VTCN1/ZBTB7B/NTRK1/DDR2/GLUL/DHX9/LAMC2/PLA2G4A/KIF14/BTG2/ATF3/DUSP10/ITPKB/WNT9A/CYP1B1/SOS1/PELI1/NCK2/BUB1/GLI2/HSPD1/FZD7/BMPR2/ERBB4/FN1/ACKR3/PER2/PLCL2/VIPR1/PRKCD/FOXP1/GATA2/IL20RB/RARRES1/BCHE/BCL6/TP63/TACC3/FGFBP1/IL8/NUDT6/SPRY1/INTU/EDNRA/IRF2/TERT/FBXO4/F2RL1/POLR3G/CDC25C/HBEGF/PDGFRB/SPARC/CNOT8/HAVCR2/SERPINB1/BMP6/GCNT2/H2AC6/BTN3A1/TNF/HSPA1B/AGER/TEAD3/SRF/FAM83B/PHIP/TTK/ASCC3/FRK/CCN2/SGK1/CITED2/ESR1/RPS6KA2/PDGFA/MAD1L1/DAGLB/HOXA5/IGFBP3/MLXIPL/DLG3/GNRH1/PTK2B/ASH2L/FGFR1/HOOK3/ST18/PLAG1/SULF1/STK3/EXT1/TRIB1/NDRG1/DOCK8/KLF9/WNK2/PTCH1/KLF4/TLR4/NACC2/SAPCD2/RTKN2/PBLD/VSIR/IFIT3/LZTS2/BTRC/E2F8/EHF/MEN1/NEAT1/LRP5/P2RY6/LRRC32/ACER3/IL18/USP28/RERG/ARID2/WNT10B/KMT2D/LMBR1L/RACGAP1/TNS2/ERBB3/E2F7/UTP20/TESC/ZNF268/RGCC/MIR17HG/GPR183/TGM1/PRKD1/EGLN3/FERMT2/ARG2/CRIP2/MEIS2/THBS1/RORA/ZNF609/SMAD3/CYP1A1/CSK/CSPG4/RASGRF1/MINAR1/IGF1R/BRICD5/NUPR1/CORO1A/ADGRG1/CDH5/PSMB10/MEAK7/CBFA2T3/BCL6B/CLDN7/LGALS9C/LGALS9B/KSR1/CDC6/KAT2A/DBF4B/HOXB4/STXBP4/BRIP1/MAP3K3/PRKCA/CEP131/MAFG/MALT1/SERPINB3/SRSF6/SULF2/NFATC2/DOT1L/FZR1/INSR/S1PR2/CALR/NACC1/CD22/PRKD2/C5AR1/ZNF304/BID/PRAME/LIF/ADAMTS1 |
| GO:1902531 | regulation of intracellular signal transduction | 202  /1427 | 0.021388043 | PRKCZ/HTR6/CAMK2N1/RAP1GAP/AGO3/PLK3/PRKAA2/GADD45A/CTH/CCN1/F3/MAGI3/HAX1/ASH1L/NTRK1/RGS2/KIF14/ATF3/DUSP10/ITPKB/RHOU/LBH/SOS1/REL/PELI1/EIF2AK3/DUSP2/SEMA4C/FHL2/NCK2/SLC20A1/FZD7/ERBB4/FN1/ACKR3/PRKCD/BCL6/TP63/NPFFR2/ANKRD17/GPAT3/FAM13A/ALPK1/SPRY1/GAB1/TRIO/C1QTNF3/PLK2/DEPDC1B/F2RL1/JMY/HBEGF/TAF7/PDGFRB/TNIP1/HAVCR2/DUSP1/SQSTM1/GCNT2/TNF/HSPA1B/AGER/STK38/HSP90AB1/EPHA7/GRIK2/TRAF3IP2/CCN2/ARFGEF3/EPM2A/AKAP12/ESR1/CNKSR3/TIAM2/PDGFA/PRKAR1B/IGFBP3/HSPB1/BMT2/GPR37/PRKAG2/ARHGAP6/FGD1/ARHGEF6/ARHGEF10/PTK2B/FGFR1/TCIM/STK3/PTP4A3/ARHGAP39/LURAP1L/MOB3B/MYORG/SEMA4D/WNK2/CAVIN4/ABCA1/KLF4/TLR4/C5/SCAI/NACC2/TRAF2/FBH1/RASGEF1A/ARHGAP22/DKK1/RTKN2/UNC5B/DDIT4/ANKRD1/ARHGAP19/BTRC/DUSP5/DUSP8/H19/MEN1/PPP2R5B/SIPA1/P2RY6/IL18/HYOU1/CBL/SORL1/ARHGAP32/ARHGDIB/FGD4/RACGAP1/MAP3K12/ERBB3/RAD9B/RHOF/UBC/STARD13/GPR183/MCF2L/PRKD1/RALGAPA1/GPR137C/FERMT2/PELI2/BDKRB2/HSP90AA1/THBS1/RORA/CSK/CSPG4/RASGRF1/MINAR1/IGF1R/LRRK1/CLEC16A/NUPR1/HERPUD1/ADGRG1/TRADD/NFAT5/BANP/PER1/MAP2K3/KSR1/NEK8/MAP3K14/BRIP1/MAP3K3/PRKCA/MAP2K6/C1QTNF1/RAC3/RIOK3/MALT1/SERPINB3/TRIB3/RBCK1/MAVS/RALGAPA2/BCL2L1/TPX2/PHF20/AURKA/LIME1/GADD45B/TNFAIP8L1/INSR/S1PR2/CALR/SYDE1/GDF15/CD22/SIPA1L3/MAP3K10/BCL3/GIPR/PRKD2/BBC3/C5AR1/MYADM/BID/LZTR1/LIF/PPARA |
| GO:2000145 | regulation of cell motility | 117  /1427 | 0.021388043 | MIIP/NBL1/EDN2/GADD45A/CCN1/F3/S100A14/DDR2/GLUL/LAMC2/PTGS2/KIF14/DUSP10/CCSAP/CYP1B1/SLC8A1/SEMA4C/IL1R1/EPB41L5/CLASP1/MGAT5/BMPR2/ERBB4/FN1/DOCK10/ACKR3/FOXP1/PLXNA1/GATA2/PFN2/FGFBP1/IL8/GAB1/TERT/PLK2/F2RL1/ARSB/HBEGF/PDGFRB/SPARC/DUSP1/GCNT2/CARMIL1/TNF/AGER/SRF/LAMA2/SGK1/CITED2/AKAP12/AFDN/PDGFA/IGFBP3/HSPB1/DOCK4/TCAF2/GNRH1/PTK2B/FGFR1/SULF1/TRIB1/DOCK8/DDX58/ARHGEF39/SEMA4D/KLF4/C5/SCAI/HSPA5/NSMF/PBLD/VSIR/SORL1/ARHGDIB/ARID2/ERBB3/ZNF268/STARD13/RGCC/GPR183/PRKD1/FERMT2/THBS1/TTBK2/ZNF609/SMAD3/DNAJA4/SEMA4B/IGF1R/BMERB1/CORO1A/ADGRG1/CDH5/TRADD/RIPOR1/PLCG2/MEAK7/CLDN7/TMEM102/MAP2K3/GRB7/KRT16/MAP3K3/PRKCA/LAMA1/SERPINB3/INSR/CAMSAP3/CALR/SYDE1/PRKD2/C5AR1/MYADM/ZNF304/GTSE1/ADAMTS1/AIRE |
| GO:0030155 | regulation of cell adhesion | 92  /1427 | 0.021388043 | PRKCZ/CCN1/VTCN1/ZBTB7B/KIF14/DUSP10/ITPKB/CYP1B1/PELI1/LOXL3/NCK2/EPB41L5/GLI2/CLASP1/PKP4/HSPD1/FZD7/FN1/FBLN2/PRKCD/CCDC80/PLXNA1/IL20RB/BCL6/IL8/NDNF/TENM3/EGFLAM/EDIL3/SPINK5/HAVCR2/DUSP1/BMP6/GCNT2/CARMIL1/TNF/AGER/SRF/EPHA7/LAMA2/CITED2/AFDN/MAD1L1/AGR2/ADAM22/ARHGAP6/TFE3/GNRH1/PTK2B/DOCK8/SEMA4D/ECM2/KLF4/GSN/VSIR/SIPA1/LRRC32/IL18/ARHGDIB/RND1/ERBB3/TESC/RGCC/FERMT2/ARG2/FMN1/THBS1/SMAD3/CSK/PEAK1/CORO1A/ADGRG1/NFAT5/CLDN7/TMEM102/LGALS9C/LGALS9B/PRKCA/UNC13D/C1QTNF1/RAC3/LAMA1/MALT1/FSTL3/CAMSAP3/CALR/NFKBID/LRFN3/PRKD2/MYADM/LIF/PPARA |
| GO:0044093 | positive regulation of molecular function | 199  /1427 | 0.023743913 | PRKCZ/CASP9/RAP1GAP/ASAP3/GRHL3/PHACTR4/EDN2/SLC6A9/GADD45A/DEPDC1/CTH/CCN1/F3/PSRC1/CRTC2/EFNA3/NTRK1/DDR2/RGS5/RASAL2/RGS16/DHX9/RGS2/KIF14/PFKFB2/MAPRE3/SOS1/EIF2AK3/EPB41L5/PKP4/HSPD1/HSPE1/ERBB4/FN1/DOCK10/PLCL2/EPM2AIP1/PRKCD/PFN2/DNAJB11/DOK7/FAM13A/CENPE/ANK2/TBC1D9/EDNRA/TERT/SNX18/PLK2/DEPDC1B/F2RL1/JMY/SERINC5/HBEGF/PDGFRB/PPP1R3G/TNF/HSPA1B/AGER/SRF/HSP90AB1/EPHA7/CCN2/SGK1/ESR1/TIAM2/AFDN/PDGFA/PRKAR1B/EPHB4/DOCK4/CFTR/PRKAG2/ARHGAP6/CDKL5/DLG3/ARHGEF6/ARHGEF10/PTK2B/FGFR1/TCIM/ST18/TCEA1/STK3/ARHGAP39/DOCK8/VLDLR/SLC1A1/DDX58/DAPK1/SEMA4D/WNK2/TBC1D2/KLF4/ZNF618/TLR4/C5/GSN/SLC27A4/GPSM1/TRAF2/ARHGAP22/DKK1/RTKN2/VSIR/ARHGAP19/BTRC/NFKB2/DUSP5/AFAP1L2/KCNJ11/PAX6/INCENP/MEN1/PPP2R5B/SIPA1/LRP5/P2RY6/IL18/KMT2A/SORL1/ARHGAP32/ARHGDIB/ADCY6/WNT10B/RACGAP1/MAP3K12/ERBB3/NAB2/APAF1/TESC/UBC/STARD13/RGCC/DOCK9/PRKD1/EGLN3/PPP2R3C/RALGAPA1/FERMT2/HSPA2/AHSA1/HSP90AA1/THBS1/PLCB2/TYRO3/TP53BP1/PARP16/SMAD3/CSK/CSPG4/RASGRF1/ARRDC4/IGF1R/PLK1/TBC1D10B/ADCY7/TRADD/MTSS2/PLCG2/CAMKK1/DLG4/MAP2K3/KSR1/GIT1/CDC6/DBF4B/MAP3K14/ARHGAP27/MAP3K3/MAP2K6/MTCL1/MALT1/SERPINB3/TRIB3/RBCK1/MAVS/RALGAPA2/TPX2/JPH2/AMH/GADD45B/FZR1/ZBTB7A/INSR/EVI5L/S1PR2/DNAJB1/SYDE1/RAB3A/GDF15/SIPA1L3/MAP3K10/PRKD2/BBC3/C5AR1/BID/H1-0/SHANK3 |
| GO:0040012 | regulation of locomotion | 120  /1427 | 0.024894843 | MIIP/NBL1/EDN2/GADD45A/CCN1/F3/S100A14/DDR2/GLUL/LAMC2/PTGS2/KIF14/DUSP10/CCSAP/CYP1B1/SLC8A1/SEMA4C/IL1R1/EPB41L5/CLASP1/MGAT5/BMPR2/ERBB4/FN1/DOCK10/ACKR3/FOXP1/PLXNA1/GATA2/PFN2/FGFBP1/IL8/GAB1/TERT/PLK2/F2RL1/ARSB/HBEGF/PDGFRB/SPARC/DUSP1/GCNT2/CARMIL1/TNF/AGER/SRF/LAMA2/SGK1/CITED2/AKAP12/AFDN/PDGFA/IGFBP3/HSPB1/DOCK4/TCAF2/GNRH1/PTK2B/FGFR1/SULF1/TRIB1/DOCK8/DDX58/ARHGEF39/SEMA4D/KLF4/C5/SCAI/HSPA5/NSMF/PBLD/VSIR/ZSWIM8/SORL1/ARHGDIB/ARID2/ERBB3/ZNF268/STARD13/RGCC/MYCBP2/GPR183/PRKD1/FERMT2/THBS1/TTBK2/ZNF609/SMAD3/DNAJA4/SEMA4B/IGF1R/BMERB1/CORO1A/ADGRG1/CDH5/TRADD/RIPOR1/PLCG2/MEAK7/CLDN7/TMEM102/MAP2K3/GRB7/KRT16/MAP3K3/PRKCA/LAMA1/SERPINB3/INSR/CAMSAP3/CALR/ZSWIM4/SYDE1/PRKD2/C5AR1/MYADM/ZNF304/GTSE1/ADAMTS1/AIRE |
| GO:0043085 | positive regulation of catalytic activity | 164  /1427 | 0.024894843 | PRKCZ/CASP9/RAP1GAP/ASAP3/GRHL3/PHACTR4/EDN2/GADD45A/DEPDC1/CCN1/F3/PSRC1/EFNA3/NTRK1/DDR2/RGS5/RASAL2/RGS16/DHX9/RGS2/KIF14/PFKFB2/MAPRE3/SOS1/EIF2AK3/PKP4/HSPD1/HSPE1/ERBB4/FN1/DOCK10/EPM2AIP1/PRKCD/PFN2/DNAJB11/DOK7/FAM13A/CENPE/TBC1D9/EDNRA/TERT/SNX18/DEPDC1B/F2RL1/SERINC5/HBEGF/PDGFRB/PPP1R3G/TNF/AGER/HSP90AB1/EPHA7/CCN2/ESR1/TIAM2/AFDN/PDGFA/PRKAR1B/EPHB4/DOCK4/PRKAG2/ARHGAP6/CDKL5/DLG3/ARHGEF6/ARHGEF10/PTK2B/FGFR1/TCIM/ST18/TCEA1/STK3/ARHGAP39/DOCK8/VLDLR/SLC1A1/DAPK1/SEMA4D/TBC1D2/KLF4/TLR4/C5/GSN/SLC27A4/TRAF2/ARHGAP22/DKK1/VSIR/ARHGAP19/BTRC/DUSP5/AFAP1L2/INCENP/SIPA1/P2RY6/IL18/SORL1/ARHGAP32/ARHGDIB/ADCY6/RACGAP1/MAP3K12/ERBB3/NAB2/APAF1/UBC/STARD13/RGCC/DOCK9/PRKD1/EGLN3/PPP2R3C/RALGAPA1/FERMT2/HSPA2/AHSA1/HSP90AA1/THBS1/PLCB2/TYRO3/PARP16/SMAD3/CSK/CSPG4/RASGRF1/ARRDC4/IGF1R/PLK1/TBC1D10B/ADCY7/TRADD/MTSS2/CAMKK1/DLG4/MAP2K3/KSR1/GIT1/CDC6/DBF4B/MAP3K14/ARHGAP27/MAP3K3/MAP2K6/MTCL1/MALT1/SERPINB3/TRIB3/RALGAPA2/TPX2/GADD45B/FZR1/INSR/EVI5L/S1PR2/DNAJB1/SYDE1/RAB3A/GDF15/SIPA1L3/MAP3K10/PRKD2/BBC3/C5AR1/BID |
| GO:0040011 | locomotion | 196  /1427 | 0.0444878 | PRKCZ/MIIP/NBL1/HTR6/RAP1GAP/ASAP3/MACO1/PHACTR4/EDN2/GADD45A/CCN1/F3/S100A14/EFNA4/EFNA3/ASH1L/NTRK1/DDR2/LHX4/GLUL/LAMC2/PTGS2/KIF14/PIK3C2B/DUSP10/RHOU/CCSAP/CYP1B1/SOS1/SLC8A1/SEMA4C/IL1R1/NCK2/EPB41L5/GLI2/CLASP1/MGAT5/RND3/FMNL2/BMPR2/ERBB4/FN1/DOCK10/ACKR3/CCRL2/PRKCD/FOXP1/CFAP44/PLXNA1/GATA2/PFN2/FGFBP1/IL8/SLC9B1/NDNF/GAB1/TERT/TRIO/PLK2/DEPDC1B/MAP1B/F2RL1/ARSB/JMY/HBEGF/PDGFRB/ANXA6/SPARC/TLX3/DUSP1/GCNT2/CARMIL1/TNF/AGER/ANKS1A/SRF/EPHA7/LAMA2/CCN2/SGK1/PEX7/CITED2/AKAP12/AFDN/PDGFA/HOXA5/ANLN/IGFBP3/HSPB1/EPHB4/DOCK4/GPR37/TCAF2/CDKL5/GNRH1/PTK2B/FGFR1/PLAT/SULF1/EXT1/TRIB1/PTP4A3/DOCK8/VLDLR/RFX3/DDX58/ARHGEF39/GCNT1/SEMA4D/PTCH1/KLF4/C5/LHX6/SCAI/HSPA5/NSMF/RASGEF1A/PBLD/UNC5B/VSIR/DDIT4/ZSWIM8/SPTBN2/LRP5/NECTIN1/SORL1/ARHGDIB/INTS13/ARID2/RND1/LIMA1/ERBB3/CFAP54/RHOF/ZNF268/STARD13/DCLK1/RGCC/MYCBP2/GPR183/PRKD1/FERMT2/THBS1/TYRO3/TTBK2/ZNF609/SMAD3/CSPG4/PEAK1/DNAJA4/SEMA4B/IGF1R/NTN3/BMERB1/ABCC1/CORO1A/ADGRG1/CDH5/TRADD/RIPOR1/PLCG2/MEAK7/SPNS2/CLDN7/TMEM102/MAP2K3/FOXN1/GRB7/KRT16/MAP3K3/PRKCA/ITGB4/CEP131/FSCN2/LAMA1/SERPINB3/THBD/PLTP/NFATC2/INSR/CAMSAP3/CALR/ZSWIM4/SYDE1/SPTBN4/PRKD2/C5AR1/MYADM/MBOAT7/ZNF304/PLA2G3/TTLL1/GTSE1/SHANK3/ADAMTS1/AIRE |
| GO:0030335 | positive regulation of cell migration | 72  /1427 | 0.048896528 | EDN2/CCN1/F3/S100A14/DDR2/LAMC2/PTGS2/SLC8A1/SEMA4C/IL1R1/EPB41L5/CLASP1/MGAT5/BMPR2/FN1/ACKR3/FOXP1/GATA2/FGFBP1/IL8/GAB1/TERT/PLK2/F2RL1/HBEGF/PDGFRB/SPARC/GCNT2/CARMIL1/TNF/AGER/AKAP12/PDGFA/HSPB1/DOCK4/TCAF2/PTK2B/FGFR1/DOCK8/ARHGEF39/SEMA4D/HSPA5/NSMF/VSIR/ZNF268/PRKD1/FERMT2/THBS1/ZNF609/SMAD3/SEMA4B/IGF1R/CORO1A/CDH5/TRADD/RIPOR1/PLCG2/TMEM102/MAP2K3/GRB7/MAP3K3/PRKCA/SERPINB3/INSR/CALR/SYDE1/PRKD2/C5AR1/MYADM/ZNF304/GTSE1/ADAMTS1 |
| GO:0006986 | response to unfolded protein | 35  /1427 | 0.049395389 | CTH/HSPA6/ATF3/EIF2AK3/NCK2/HSPD1/HSPE1/MANF/DNAJB11/IL8/HSPA4L/CREBRF/HSPA1B/HSP90AB1/AGR2/HSPB1/ASNS/BHLHA15/DNAJB9/HSPA5/BAG3/PPP2R5B/SERPINH1/HYOU1/HSPA8/HSP90B1/FICD/HSPA2/HSP90AA1/THBS1/PARP16/HERPUD1/WIPI1/CALR/DNAJB1 |
| GO:0051272 | positive regulation of cellular component movement | 75  /1427 | 0.049395389 | EDN2/CCN1/F3/S100A14/DDR2/LAMC2/PTGS2/SLC8A1/SEMA4C/IL1R1/EPB41L5/CLASP1/MGAT5/BMPR2/FN1/ACKR3/FOXP1/GATA2/BCL6/FGFBP1/IL8/GAB1/TERT/PLK2/F2RL1/HBEGF/PDGFRB/SPARC/GCNT2/CARMIL1/TNF/AGER/AKAP12/PDGFA/HSPB1/DOCK4/TCAF2/PTK2B/FGFR1/DOCK8/ARHGEF39/SEMA4D/HSPA5/NSMF/VSIR/ZNF268/PRKD1/FERMT2/THBS1/ZNF609/SMAD3/SEMA4B/IGF1R/CORO1A/CDH5/TRADD/RIPOR1/PLCG2/CLDN7/TMEM102/MAP2K3/GRB7/TTLL6/MAP3K3/PRKCA/SERPINB3/INSR/CALR/SYDE1/PRKD2/C5AR1/MYADM/ZNF304/GTSE1/ADAMTS1 |
| GO:2000026 | regulation of multicellular organismal development | 146  /1427 | 0.049395389 | PRKCZ/ADGRB2/AGO3/HEYL/MPL/PTCH2/GADD45A/ADGRL2/CCN1/F3/SARS1/HAX1/ZBTB7B/EFNA3/NTRK1/DDR2/GLUL/RGS2/KIF14/DUSP10/ITPKB/WNT9A/CCSAP/MBOAT2/CYP1B1/SOS1/SLC8A1/PKDCC/LOXL3/HK2/EIF2AK3/SEMA4C/GLI2/CLASP1/LNPK/BMPR2/ERBB4/FN1/PER2/OXTR/TCTA/FOXP1/ZBED2/PLXNA1/GATA2/BCL6/TP63/IL8/SPRY1/GAB1/TPPP/TERT/PLK2/MAP1B/FBN2/SPINK5/SPARC/BMP6/H4C8/TNF/HSPA1B/FKBPL/AGER/SRF/EPHA7/LAMA2/CCN2/TIAM2/THBS2/HOXA5/HSPB1/KMT2C/CDKL5/TFE3/GPRASP2/PTK2B/ASH2L/TCIM/HOOK3/PLAG1/SULF1/HEY1/TRPS1/TRIB1/RFX3/LINGO2/AQP3/SEMA4D/PTCH1/KLF4/TLR4/C5/VSIR/PAX6/IL18/KMT2A/SORL1/BHLHE41/WNT10B/KMT2D/RACGAP1/RBM19/TESC/RGCC/MIR17HG/PRKD1/PPP2R3C/MEIS2/THBS1/SMAD3/SIN3A/MINAR1/SEMA4B/NUPR1/CDH5/TRADD/NLGN2/HES7/TRIM16/EPN2/FOXN1/KAT2A/PRKCA/MAFG/LAMA1/MALT1/MAFB/SRSF6/NFATC2/ZBTB46/FSTL3/INSR/S1PR2/KLF2/HSPB6/NFKBID/PRKD2/C5AR1/ZNF304/LIF/PLA2G3/TNRC6B/PPARA/ADM2/SHANK3/ADAMTS1 |
| GO:0048468 | cell development | 214  /1427 | 0.049395389 | PRKCZ/CASP9/NBL1/RAP1GAP/PHACTR4/HEYL/MPL/HORMAD1/EFNA4/EFNA3/TMEM79/NTRK1/LHX4/LAMC2/RGS2/CRB1/KIF14/BTG2/RAB29/DUSP10/SOS1/SLC8A1/EPAS1/KDM3A/EIF2AK3/SEMA4C/FHL2/NCK2/EPB41L5/GLI2/HECW2/FZD7/BMPR2/ERBB4/FN1/DOCK10/RAB17/PER2/HDAC11/SLC4A7/MANF/FOXP1/CFAP44/PLXNA1/GATA2/PLS1/BCL6/TP63/FRYL/ANK2/NDNF/SPRY1/GAB1/EDNRA/TENM3/SORBS2/TPPP/TRIO/PLK2/MAP1B/F2RL1/ARSB/NREP/SH3TC2/PDGFRB/TCOF1/ANXA6/BMP6/CARMIL1/TNF/AGER/PBX2/SPDEF/ANKS1A/CPNE5/SRF/HSP90AB1/EPHA7/LAMA2/L3MBTL3/SGK1/ESR1/TIAM2/AFDN/HOXA5/COBL/ADAM22/BHLHA15/EPHB4/CFTR/MNX1/FAM9B/CDKL5/MED12/GPRASP2/ARHGEF10/PTK2B/KIF13B/HOOK3/PLAG1/SULF1/HEY1/CSMD3/EXT1/TRIB1/NDRG1/VLDLR/RFX3/MYORG/SEMA4D/PTCH1/KLF4/TLR4/GSN/LHX6/HSPA5/PBX3/NCS1/TPRN/NSMF/CDNF/UNC5B/ZSWIM8/LDB3/ACTA2/ANKRD1/PDZD7/KNDC1/BRSK2/DENND5A/USH1C/PAX6/MEN1/PPP2R5B/SPTBN2/LRP5/SERPINH1/EXPH5/NECTIN1/SORL1/ST14/FKBP4/BHLHE41/ADCY6/RND1/WNT10B/KMT2D/KRT8/CSRP2/DCLK1/MYCBP2/PRKD1/FERMT2/C14orf39/HSPA2/DIO3/HSP90AA1/TYRO3/SMAD3/SIN3A/PTPN9/PEAK1/RASGRF1/MINAR1/SEMA4B/LRRK1/DNASE1L2/NTN3/STX1B/CNGB1/CDH5/DLG4/HES7/PLD6/SARM1/GIT1/GRB7/BRIP1/PRKCA/ITGB4/UNC13D/NPTX1/CEP131/FSCN2/RAC3/LAMA1/IMPACT/B4GALT6/ALPK2/CBFA2T2/SULF2/NFATC2/AURKA/AMH/TJP3/ZBTB7A/CAMSAP3/S1PR2/CALR/ZSWIM4/KLF2/UNC13A/RAB3A/SIPA1L3/SPTBN4/C5AR1/MYADM/LIF/PLA2G3/TTLL1/PPARA/MOV10L1/SHANK3/DIP2A |
| GO:1902947 | regulation of tau-protein kinase activity | 6  /1427 | 0.049395389 | HSP90AB1/DKK1/SORL1/NAB2/HSP90AA1/C5AR1 |
| GO:0044262 | cellular carbohydrate metabolic process | 44  /1427 | 0.049395389 | SLC2A1/RORC/PLA2G4A/SLC45A3/PFKFB2/RBKS/HK2/IDH1/PER2/EPM2AIP1/PFKFB4/C1QTNF3/PPP1R3G/IGFBP3/PRKAG2/GK/PPP1R3B/CSGALNACT1/PTK2B/NCOA2/EXT1/IDNK/HKDC1/DDIT4/PPP1R3C/GOT1/P2RY6/PGM2L1/DGAT2/GNPTAB/B3GNT4/PCK2/RORA/NUPR1/B3GNT9/HAS3/CBFA2T3/GIT1/KAT2A/C1QTNF1/SOGA1/INSR/GYS1/PPARA |
| GO:2000147 | positive regulation of cell motility | 73  /1427 | 0.049395389 | EDN2/CCN1/F3/S100A14/DDR2/LAMC2/PTGS2/SLC8A1/SEMA4C/IL1R1/EPB41L5/CLASP1/MGAT5/BMPR2/FN1/ACKR3/FOXP1/GATA2/FGFBP1/IL8/GAB1/TERT/PLK2/F2RL1/HBEGF/PDGFRB/SPARC/GCNT2/CARMIL1/TNF/AGER/AKAP12/PDGFA/HSPB1/DOCK4/TCAF2/PTK2B/FGFR1/DOCK8/ARHGEF39/SEMA4D/HSPA5/NSMF/VSIR/ZNF268/PRKD1/FERMT2/THBS1/ZNF609/SMAD3/SEMA4B/IGF1R/CORO1A/CDH5/TRADD/RIPOR1/PLCG2/CLDN7/TMEM102/MAP2K3/GRB7/MAP3K3/PRKCA/SERPINB3/INSR/CALR/SYDE1/PRKD2/C5AR1/MYADM/ZNF304/GTSE1/ADAMTS1 |

**Table S6.** Biological processes with the most affected genes in MKN-28 after 6-hour infection with *H. pylori* Δ*htrA*, as determined by overrepresentation analysis.

| **ID** | **Biological process** | **Gene ratio** | **P_adj_** | **Affected genes** |
| --- | --- | --- | --- | --- |
| GO:0048646 | anatomical structure formation involved in morphogenesis | 141  /1509 | 0.007142716 | CASP9/HSPG2/PHACTR4/ADGRB2/HEYL/MPL/PIK3R3/GADD45A/PRKACB/CCN1/F3/SARS1/EFNA3/GLUL/PTGS2/CRB1/IGFN1/CYP1B1/EPAS1/HK2/EIF2AK3/DUSP2/SEMA4C/FHL2/PAX8/EPB41L5/GLI2/CLASP1/COL5A2/FZD7/BMPR2/ACKR3/COL7A1/TCTA/GATA2/SKIL/BCL6/FGFBP1/IL8/NDNF/SPRY1/GAB1/EDNRA/TPPP/PLK2/ERAP1/FBN2/SPINK5/PDGFRB/TCOF1/SPARC/POU5F1/TNF/FKBPL/SRF/HEY2/RSPO3/CCN2/TNFAIP3/CITED2/THBS2/C1GALT1/COBL/EPHB4/TSPAN12/HIPK2/PTK2B/SULF1/HEY1/STK3/EXT1/SLC1A1/AGTPBP1/PTCH1/KLF4/ARHGAP22/DKK1/LDB3/KIF20B/ANKRD1/NFKB2/SUFU/SH3PXD2A/DUSP5/KNDC1/E2F8/FJX1/IL18/NECTIN1/CDON/ST14/WNT10B/FAIM2/KRT8/CSRP2/E2F7/ELK3/APAF1/STARD13/RGCC/PRKD1/WARS1/FMN1/THBS1/RORA/SMAD3/CSPG4/MINAR1/BMERB1/ADGRG1/MMP15/CDH5/KDM6B/HES7/EPN2/MAPK7/KAT2A/HOXB3/MAP3K3/PRKCA/SOX9/ITGB4/UNC13D/ID1/MAFB/NFATC2/JUNB/KLF2/GDF15/HSPB6/BCL3/HIF3A/PRKD2/C5AR1/MBOAT7/PLA2G3/CCDC134/TTLL1/PPARA/ADM2/ADAMTS1 |
| GO:0035295 | tube development | 137  /1509 | 0.007142716 | HSPG2/PHACTR4/ADGRB2/HEYL/EDN2/PIK3R3/GADD45A/PRKACB/CCN1/F3/SARS1/EFNA3/GPR161/GLUL/PTGS2/CYP1B1/SOS1/PKDCC/EPAS1/LOXL3/HK2/EIF2AK3/SEMA4C/PAX8/EPB41L5/GLI2/BMPR2/CREB1/ABCA12/ACKR3/OXTR/FOXP1/GATA2/TIPARP/FGFBP1/IL8/NDNF/SPRY1/INTU/SLC7A11/GAB1/EDNRA/ADAMTS16/PLK2/ERAP1/MAN2A1/SPINK5/PDGFRB/SPARC/TNF/FKBPL/SPDEF/SRF/EPHA7/HEY2/RSPO3/CCN2/TNFAIP3/HECA/CITED2/ESR1/THBS2/C1GALT1/AGR2/COBL/EPHB4/TSPAN12/HIPK2/SLC4A2/PTK2B/EIF4EBP1/SULF1/HEY1/STK3/EXT1/SLC1A1/PTCH1/KLF4/ARHGAP22/KIF20B/LZTS2/BTRC/SUFU/E2F8/KIF18A/LRP5/IL18/ST14/ARID2/E2F7/ELK3/APAF1/STARD13/RGCC/MIR17HG/PRKD1/ARG2/WARS1/FMN1/THBS1/RORA/SMAD3/CYP1A1/CSPG4/MINAR1/ABCA3/RPGRIP1L/ADGRG1/CDH5/CRISPLD2/FOXL1/EPN2/MAPK7/FOXN1/KAT2A/GJC1/HOXB3/BRIP1/MAP3K3/PRKCA/SOX9/LAMA1/ID1/CBFA2T2/SRSF6/FSTL3/JUNB/KLF2/RAB3A/HSPB6/HIF3A/PRKD2/C5AR1/LIF/CCDC134/ADM2/ADAMTS1 |
| GO:2000026 | regulation of multicellular organismal development | 160  /1509 | 0.020828601 | PRKCZ/HSPG2/ADGRB2/AGO3/HEYL/MPL/PTCH2/GADD45A/ADGRL2/CCN1/F3/SARS1/ZBTB7B/EFNA3/NTRK1/DDR2/GLUL/RGS2/KIF14/DUSP10/ITPKB/WNT9A/CCSAP/MBOAT2/GRHL1/CYP1B1/SOS1/SLC8A1/PKDCC/LOXL3/HK2/EIF2AK3/SEMA4C/PAX8/GLI2/CLASP1/LNPK/BMPR2/CREB1/ERBB4/PER2/OXTR/TCTA/MITF/FOXP1/ZBED2/PLXNA1/GATA2/SKIL/BCL6/RUFY3/IL8/SLC9B2/SPRY1/GAB1/HMGB2/TPPP/PLK2/MAP1B/MAN2A1/FBN2/SPINK5/SPARC/BMP6/H4C8/TNF/HSPA1B/FKBPL/AGER/SRF/EPHA7/HEY2/LAMA2/CCN2/TNFAIP3/TIAM2/THBS2/TSPAN12/HIPK2/KMT2C/CDKL5/TFE3/GPRASP2/PTK2B/ASH2L/TCIM/HOOK3/PLAG1/SULF1/HEY1/TRPS1/TRIB1/LRRC24/RFX3/ZDHHC21/AQP3/PTCH1/KLF4/CDK1/VSIR/PAX6/CCND1/IL18/KMT2A/SORL1/BHLHE41/WNT10B/KMT2D/RACGAP1/RBM19/TESC/RGCC/MIR17HG/PCID2/PRKD1/PPP2R3C/NFKBIA/NIN/WARS1/MEIS2/THBS1/SMAD3/SIN3A/MINAR1/SEMA4B/NUPR1/CDH5/NLGN2/HES7/TRIM16/EPN2/MAPK7/FOXN1/KAT2A/HOXB3/PRKCA/SOX9/MAFG/LAMA1/MALT1/RASSF2/ID1/MAFB/SRSF6/NFATC2/ZBTB46/FSTL3/INSR/KLF2/HSPB6/NFKBID/PRKD2/C5AR1/LIF/PLA2G3/TNRC6B/PPARA/ADM2/SHANK3/ADAMTS1 |
| GO:0050793 | regulation of developmental process | 271  /1509 | 0.022081758 | PRKCZ/CASZ1/NBL1/HSPG2/RPS6KA1/ADGRB2/ZMYM4/AGO3/HEYL/MPL/PTCH2/GADD45A/CTH/ADGRL2/CCN1/F3/PALMD/SARS1/CELSR2/RORC/ZBTB7B/EFNA3/TMEM79/NTRK1/DDR2/GLUL/PTGS2/BRINP3/RGS2/KIF14/SLC45A3/CENPF/MARK1/DUSP10/ITPKB/WNT9A/RHOU/CCSAP/MBOAT2/GRHL1/CYP1B1/SOS1/SLC8A1/PKDCC/LOXL3/HK2/KDM3A/EIF2AK3/SEMA4C/PAX8/GLI2/CLASP1/LIMS2/RND3/FMNL2/LNPK/COL5A2/HECW2/FZD7/BMPR2/CREB1/ERBB4/TMBIM1/PER2/OXTR/TCTA/MITF/FOXP1/ZBED2/PLXNA1/GATA2/PLS1/SKIL/BCL6/RUFY3/ANKRD17/IL8/SLC9B2/SPRY1/GAB1/HMGB2/TPPP/PLK2/MAP1B/MAN2A1/NREP/FBN2/SPINK5/SPARC/BMP6/GCNT2/H4C8/TNF/HSPA1B/FKBPL/AGER/SPDEF/SRF/RUNX2/PHIP/EPHA7/HEY2/RSPO3/LAMA2/CCN2/TNFAIP3/CITED2/ESR1/TIAM2/SOD2/THBS2/AGR2/IGFBP3/COBL/BHLHA15/TSPAN12/HIPK2/KMT2C/CDKL5/TFE3/FGD1/ZMYM3/BRWD3/GPRASP2/GNRH1/PTK2B/KIF13B/ASH2L/FGFR1/TCIM/HOOK3/PLAG1/SULF1/HEY1/STK3/CSMD3/TRPS1/TRIB1/LRRC24/VLDLR/RFX3/ZDHHC21/AQP3/TESK1/PTCH1/ABCA1/KLF4/SAPCD2/NSMF/DKK1/CDK1/PBLD/VSIR/ANKRD2/DNMBP/SUFU/KNDC1/PAX6/MEN1/LRP5/CCND1/IL18/KMT2A/SORL1/CDON/BHLHE41/FGD4/RND1/WNT10B/KMT2D/RACGAP1/ERBB3/NAB2/RBM19/TESC/RHOF/ZNF268/POSTN/RGCC/MIR17HG/PCID2/PRKD1/PPP2R3C/NFKBIA/NIN/FERMT2/ARG2/WARS1/DIO3/MEIS2/THBS1/TP53BP1/RORA/SMAD3/SIN3A/MINAR1/SEMA4B/NUPR1/CORO1A/CDH5/PSMB10/EXOSC6/CDH15/CYB5D2/DLG4/KCTD11/NLGN2/HES7/TRIM16/PLD6/EPN2/MAPK7/SARM1/FOXN1/KRT10/KAT2A/HOXB3/HOXB4/TOB1/MAP3K3/PRKCA/ABCA5/SOX9/UNC13D/SOCS3/MAFG/RAC3/LAMA1/IMPACT/ALPK2/MALT1/SERPINB3/TRIB3/RASSF2/ID1/MAFB/SRSF6/JPH2/NFATC2/AURKA/ZBTB46/FSTL3/AMH/FZR1/INSR/JUNB/CALR/SYDE1/KLF2/UNC13A/GDF15/LRP3/WTIP/HSPB6/NFKBID/PSMC4/PRKD2/C5AR1/MAMSTR/MYADM/PRAME/LIF/PLA2G3/TRIOBP/TNRC6B/TTLL1/PPARA/ADM2/SHANK3/ADAMTS1/ABCG1 |
| GO:0010941 | regulation of cell death | 194  /1509 | 0.025792027 | PRKCZ/TNFRSF25/CASP9/ATP13A2/RPS6KA1/PRKAA2/GADD45A/CTH/CCN1/VTCN1/NTRK1/KIF14/BTG2/NUAK2/RAB29/FCMR/ATF3/ITPKB/CYP1B1/SOS1/GPR75/REL/PELI1/TIA1/EIF2AK3/FHL2/NCK2/PAX8/STEAP3/GLI2/LIMS2/CSRNP3/SLC39A10/HSPD1/CREB1/TMBIM1/ACKR3/CAPN10/PRKCD/MITF/FOXP1/GATA2/SKIL/BCL6/TLR6/SNCA/NDNF/SLC7A11/HMGB2/CARD6/PLK2/JMY/TNFAIP8/PDGFRB/PRR7/SQSTM1/PPP1R10/IER3/TNF/HSPA1B/SPDEF/PHIP/EPHA7/GRIK2/HEY2/CCN2/SGK1/TNFAIP3/CITED2/AKAP12/ESR1/TIAM2/SOD2/RPS6KA2/AGR2/IGFBP3/ASNS/ING3/GPR37/HIPK2/FGD1/ARHGEF9/GPRASP2/ARHGEF6/GNRH1/PTK2B/RNF122/FGFR1/TCIM/STK3/NDRG1/DOCK8/DAPK1/KLF4/GSN/SLC27A4/NACC2/TRAF2/NSMF/FBH1/SGMS1/DKK1/CDK1/RTKN2/AIFM2/DDIT4/IFIT2/ANKRD1/CALHM2/ITPRIP/BAG3/LRP5/ACER3/BIRC3/HYOU1/CBL/SORL1/FGD4/WNT10B/FAIM2/MAP3K12/ERBB3/PHLDA1/APAF1/HSP90B1/UBC/ZNF268/RGCC/MIR17HG/ING1/MCF2L/PCID2/PRKD1/EGLN3/NFKBIA/FERMT2/ARG2/BDKRB2/HSP90AA1/THBS1/TYRO3/DAPK2/PARP16/SMAD3/SIN3A/IGF1R/PLK1/NUPR1/CORO1A/HERPUD1/CDH5/PLCG2/MEAK7/CLDN7/TMEM102/MAPK7/SARM1/TOP2A/PRKCA/MAP2K6/SOX9/SOCS3/CBX4/IMPACT/ALPK2/MALT1/RBCK1/RASSF2/ID1/BCL2L1/SRSF6/AURKA/GADD45B/ZBTB7A/TNFAIP8L1/CDKN2D/CALR/HSPB6/ZNF420/MAP3K10/EGLN2/DEDD2/BCL3/MARK4/BBC3/C5AR1/CARD8/BID/PRODH/PRAME/PLA2G3/IL2RB/BIK/PPARA |
| GO:0051239 | regulation of multicellular organismal process | 281  /1509 | 0.027175074 | PRKCZ/HSPG2/GPR3/ADGRB2/DLGAP3/AGO3/HEYL/EDN2/SVBP/ERMAP/MPL/PTCH2/MAST2/CPT2/GADD45A/ADGRL2/CCN1/F3/SARS1/KCND3/VTCN1/ZBTB7B/EFNA3/NTRK1/DDR2/GLUL/DHX9/PTGS2/RGS2/KIF14/DUSP10/ITPKB/WNT9A/CCSAP/MBOAT2/GRHL1/CYP1B1/SOS1/SLC8A1/PKDCC/EPAS1/REL/PELI1/TIA1/LOXL3/HK2/KDM3A/EIF2AK3/SEMA4C/IL1R2/IL1RL1/PAX8/EPB41L5/GLI2/CLASP1/LNPK/HSPD1/FZD7/BMPR2/CREB1/ERBB4/TMBIM1/PER2/OXTR/PLCL2/TCTA/UBA7/PRKCD/MITF/FOXP1/ZBED2/PLXNA1/GATA2/IL20RB/PLS1/PFN2/SKIL/BCL6/FGFBP1/TLR6/RUFY3/IL8/SLC9B2/ANK2/SPRY1/INTU/GAB1/HMGB2/TPPP/C1QTNF3/PLK2/MAP1B/ARSB/POLR3G/LNPEP/MAN2A1/FBN2/HBEGF/SPINK5/ANXA6/SPARC/HAVCR2/SERPINB1/BMP6/GCNT2/H4C8/BTN3A1/TNF/HSPA1B/FKBPL/AGER/GPSM3/SRF/EPHA7/HEY2/LAMA2/CCN2/SGK1/TNFAIP3/CITED2/ESR1/TIAM2/THBS2/SRI/AKAP9/DOCK4/TSPAN12/HIPK2/KMT2C/CDKL5/TFE3/GPRASP2/PTK2B/ASH2L/FGFR1/TCIM/PLAT/HOOK3/PLAG1/SULF1/HEY1/STK3/TRPS1/FBXO32/TRIB1/LRRC24/RFX3/SLC1A1/ZDHHC21/DDX58/AQP3/PTCH1/ABCA1/KLF4/TRAF2/CACNB2/DKK1/CDK1/PBLD/VSIR/NFKB2/KCNQ1/KCNJ11/PAX6/DTX4/FRMD8/CCND1/LRRC32/IL18/KMT2A/SORL1/CDON/ARHGDIB/SPX/BHLHE41/ITPR2/WNT10B/KMT2D/RACGAP1/ERBB3/RBM19/TESC/STARD13/POSTN/RGCC/MIR17HG/PCID2/PRKD1/PPP2R3C/NFKBIA/NIN/FERMT2/ARG2/DIO2/BDKRB2/WARS1/DIO3/DYNC1H1/HSP90AA1/MEIS2/THBS1/TP53BP1/RORA/SMAD3/CSK/SIN3A/DNAJA4/MINAR1/SEMA4B/IGF1R/KREMEN2/NUPR1/STX1B/ADCY7/CDH5/EXOSC6/PLCG2/TRPV3/ATP2A3/DLG4/NLGN2/KDM6B/HES7/PER1/TRIM16/LGALS9C/EPN2/MAPK7/LGALS9B/MAP2K3/FOXN1/GIT1/KAT2A/GJC1/HOXB3/PCTP/MTMR4/MAP3K3/SCN4A/PRKCA/MAP2K6/SOX9/C1QTNF1/NPTX1/MAFG/LAMA1/RIOK3/ALPK2/MALT1/SERPINB3/MAVS/RASSF2/THBD/ID1/DLGAP4/MAFB/SRSF6/NFATC2/ZBTB46/FSTL3/INSR/CALR/SYDE1/KLF2/SLC27A1/GDF15/HSPB6/NFKBID/BCL3/PRKD2/C5AR1/CARD8/KCNJ14/LIF/PLA2G3/TNRC6B/PPARA/ADM2/SHANK3/ADAMTS1/KCNE1 |
| GO:0030155 | regulation of cell adhesion | 96  /1509 | 0.027175074 | PRKCZ/CCN1/CELSR2/VTCN1/ZBTB7B/KIF14/DUSP10/ITPKB/CYP1B1/PELI1/LOXL3/NCK2/EPB41L5/GLI2/CLASP1/LIMS2/PKP4/HSPD1/FZD7/FBLN2/PRKCD/PLXNA1/IL20RB/BCL6/IL8/NDNF/TENM3/EGFLAM/EDIL3/SPINK5/HAVCR2/BMP6/GCNT2/TNF/AGER/SRF/EPHA7/LAMA2/CITED2/AFDN/MAD1L1/AGR2/ADAM22/ARHGAP6/TFE3/GNRH1/PTK2B/DOCK8/ZDHHC21/TESK1/ECM2/KLF4/GSN/VSIR/LRRC32/IL18/ARHGDIB/RND1/ERBB3/TESC/POSTN/RGCC/FERMT2/ARG2/FMN1/THBS1/SMAD3/CSK/PEAK1/CORO1A/ADGRG1/NFAT5/CLDN7/TMEM102/LGALS9C/MAPK7/LGALS9B/PRKCA/SOX9/UNC13D/C1QTNF1/RAC3/LAMA1/ONECUT2/MALT1/FSTL3/CAMSAP3/CALR/NFKBID/LRFN3/PRKD2/MYADM/LIF/TRIOBP/TNFRSF13C/PPARA |
| GO:0003018 | vascular process in circulatory system | 40  /1509 | 0.028933443 | EDN2/SLC2A1/SLC6A9/RGS2/SLC8A1/BMPR2/PER2/OXTR/ABCC5/UTS2B/ABCG2/EDNRA/BMP6/AGER/SLC29A1/AKAP12/SOD2/SLC22A3/DOCK4/EXT1/SLC1A1/ZDHHC21/SLC27A4/ACTA2/SLC2A3/SLC2A13/ADCY6/SLC7A8/FERMT2/BDKRB2/ABCC1/CDH5/ATP2A3/SLC2A4/TJP3/INSR/KLF2/SLC27A1/LRP3/SLC19A1 |
| GO:0008283 | cell population proliferation | 210  /1509 | 0.029266093 | PRKCZ/FBXO2/CAMK2N1/IFNLR1/RPS6KA1/FABP3/AGO3/EDN2/MPL/CCN1/F3/VTCN1/BNIPL/ZBTB7B/NTRK1/DDR2/GLUL/DHX9/LAMC2/PLA2G4A/KIF14/BTG2/ATF3/DUSP10/ITPKB/WNT9A/CYP1B1/SOS1/PELI1/NCK2/BUB1/GLI2/LIMS2/SLC39A10/HSPD1/FZD7/BMPR2/CREB1/ERBB4/ACKR3/PER2/PLCL2/VIPR1/PRKCD/MITF/FOXP1/GATA2/IL20RB/RARRES1/BCHE/BCL6/TACC3/FGFBP1/IL8/SPRY1/INTU/SLC7A11/EDNRA/HMGB2/IRF2/FBXO4/POLR3G/HBEGF/PDGFRB/SPARC/CNOT8/HAVCR2/SERPINB1/BMP6/GCNT2/H2AC6/BTN3A1/TNF/HSPA1B/AGER/TEAD3/SRF/FAM83B/PHIP/TTK/ASCC3/FRK/HEY2/CCN2/SGK1/TNFAIP3/CITED2/ESR1/SOD2/RPS6KA2/CEP43/MAD1L1/DAGLB/IGFBP3/MLXIPL/HIPK2/DLG3/GNRH1/PTK2B/ASH2L/FGFR1/HOOK3/PLAG1/SULF1/STK3/EXT1/TRIB1/NDRG1/DOCK8/KLF9/WNK2/PTCH1/KLF4/NACC2/SAPCD2/RTKN2/PBLD/VSIR/KIF20B/LZTS2/BTRC/E2F8/EHF/ALKBH3/MEN1/LRP5/CCND1/LRRC32/ACER3/IL18/USP28/CDON/ARID2/WNT10B/KMT2D/LMBR1L/RACGAP1/TNS2/ERBB3/E2F7/UTP20/TESC/ZNF268/RGCC/MIR17HG/GPR183/ING1/TGM1/PRKD1/EGLN3/NFKBIA/FERMT2/ARG2/WARS1/MEIS2/THBS1/RORA/ZNF609/SMAD3/CYP1A1/CSK/CSPG4/RASGRF1/MINAR1/IGF1R/BRICD5/NUPR1/CORO1A/ADGRG1/CDH5/PSMB10/PDF/MEAK7/CBFA2T3/CLDN7/GLP2R/LGALS9C/LGALS9B/KSR1/CDC6/KAT2A/DBF4B/HOXB4/TOB1/STXBP4/BRIP1/MAP3K3/PRKCA/SOX9/CEP131/MAFG/MALT1/SERPINB3/ID1/SRSF6/NFATC2/FZR1/INSR/CDKN2D/JUNB/CALR/NACC1/CD22/PRKD2/C5AR1/BID/PRAME/LIF/TNFRSF13C/ADAMTS1 |
| GO:0030334 | regulation of cell migration | 115  /1509 | 0.029266093 | MIIP/NBL1/EDN2/SVBP/PIK3R3/GADD45A/CCN1/F3/S100A14/DDR2/GLUL/LAMC2/PTGS2/KIF14/DUSP10/CYP1B1/SLC8A1/SEMA4C/EPB41L5/CLASP1/MGAT5/BMPR2/ERBB4/DOCK10/ACKR3/MITF/FOXP1/PLXNA1/GATA2/PFN2/FGFBP1/RUFY3/IL8/GAB1/PLK2/ARSB/HBEGF/PDGFRB/SPARC/GCNT2/TNF/AGER/GPSM3/SRF/LAMA2/SGK1/CITED2/AKAP12/SOD2/CEP43/AFDN/IGFBP3/DOCK4/TCAF2/MPP1/GNRH1/PTK2B/FGFR1/SULF1/CPNE3/TRIB1/DOCK8/DDX58/ARHGEF39/KLF4/SCAI/NSMF/PBLD/VSIR/KIF20B/SORL1/ARHGDIB/ARID2/ZNF268/STARD13/POSTN/RGCC/GPR183/PRKD1/FERMT2/THBS1/TTBK2/DAPK2/ZNF609/SMAD3/DNAJA4/SEMA4B/IGF1R/BMERB1/CORO1A/ADGRG1/CDH5/RIPOR1/PLCG2/MEAK7/TMEM102/MAP2K3/GRB7/KRT16/MAP3K3/PRKCA/SOX9/LAMA1/ONECUT2/SERPINB3/INSR/CAMSAP3/CALR/SYDE1/PRKD2/C5AR1/MYADM/GTSE1/ADAMTS1/AIRE |
| GO:0009888 | tissue development | 215  /1509 | 0.029266093 | PLOD1/HSPG2/ZBTB40/PHACTR4/HEYL/HIVEP3/PTCH2/PRKACB/CCN1/CELSR2/TUFT1/RORC/ZBTB7B/ASH1L/TMEM79/NTRK1/DDR2/GPR161/LAMC2/RGS2/ELF3/BTG2/ATF3/CENPF/DUSP10/WNT9A/MBOAT2/GRHL1/CYP1B1/SOS1/SLC8A1/PKDCC/LOXL3/VAMP5/EIF2AK3/DUSP2/SEMA4C/FHL2/SLC20A1/PAX8/EPB41L5/GLI2/CLASP1/PKP4/LNPK/COL5A2/FZD7/BMPR2/CREB1/ERBB4/ABCA12/CRELD1/SLC4A7/COL7A1/FOXP1/ZBED2/PLXNA1/PLS1/TIPARP/SKIL/SPRY1/INTU/SLC7A11/EDNRA/SORBS2/ADAMTS16/EGFLAM/PTCD2/FBN2/HBEGF/SPINK5/PDGFRB/TCOF1/ANXA6/STC2/BMP6/GCNT2/POU5F1/TNF/FKBPL/SPDEF/SRF/RUNX2/EYS/EPHA7/HEY2/RSPO3/LAMA2/CCN2/CITED2/ESR1/AFDN/SNX10/COBL/CSGALNACT1/PTK2B/FGFR1/SULF1/HEY1/STK3/TRPS1/EXT1/RFX3/ZDHHC21/AQP3/MYORG/GCNT1/PTCH1/KLF4/GSN/SAPCD2/TPRN/DKK1/CDK1/PBLD/ACTA2/KIF20B/ANKRD1/LZTS2/PDZD7/BTRC/SUFU/DUSP5/DMBT1/USH1C/E2F8/PAX6/EHF/LRP5/CCND1/DGAT2/EXPH5/NECTIN1/SORL1/CDON/ST14/ARID2/WNT10B/KRT80/KRT86/KRT83/KRT8/ERBB3/NAB2/CSRP2/E2F7/APAF1/STARD13/POSTN/RGCC/SCEL/MIR17HG/PCK2/TGM1/FERMT2/ARID4A/ARG2/FMN1/SMAD3/CYP1A1/SEMA4B/CCDC78/DNASE1L2/NTN3/PPL/RBBP6/NUPR1/RPGRIP1L/MMP15/CDH5/PSMB10/ATP2C2/FOXL1/KDM6B/HES7/TRIM16/EVPLL/FOXN1/KRT10/KRT34/KRT16/KAT2A/GJC1/HOXB3/HOXB4/HOXB5/HLF/SOX9/ITGB4/SOCS3/MAFG/LAMA1/ONECUT2/ALPK2/SERPINB3/GPCPD1/ID1/CBFA2T2/SRSF6/JPH2/AMH/FZR1/TJP3/CAMSAP3/JUNB/CALR/KLF2/SIPA1L3/PSMC4/PRKD2/MYADM/LIF/TRIOBP/PPARA/AIRE |
| GO:0008219 | cell death | 246  /1509 | 0.029266093 | PRKCZ/TNFRSF25/CASP9/ATP13A2/RPS6KA1/PRKAA2/GADD45A/CTH/CCN1/MAGI3/VTCN1/BNIPL/S100A14/TMEM79/NTRK1/DHX9/KIF14/BTG2/NUAK2/RAB29/FCMR/ATF3/ITPKB/CYP1B1/SOS1/GPR75/REL/PELI1/TIA1/HK2/EIF2AK3/FHL2/NCK2/BUB1/PAX8/STEAP3/GLI2/LIMS2/PKP4/CSRNP3/GULP1/SLC39A10/HSPD1/BMPR2/CREB1/ERBB4/TMBIM1/ACKR3/CAPN10/PRKCD/MITF/FOXP1/GATA2/SKIL/BCL6/TLR6/SNCA/NDNF/SLC7A11/HMGB2/CARD6/PLK2/JMY/TNFAIP8/SPINK5/PDGFRB/ANXA6/PRR7/SQSTM1/PPP1R10/IER3/TNF/HSPA1B/SPDEF/PHIP/EPHA7/GRIK2/TRAF3IP2/HEY2/CCN2/SGK1/TNFAIP3/CITED2/PLAGL1/AKAP12/ESR1/TIAM2/SOD2/RPS6KA2/C6orf120/AGR2/IGFBP3/ASNS/ING3/GPR37/HIPK2/FGD1/ARHGEF9/GPRASP2/ARHGEF6/GNRH1/PTK2B/RNF122/FGFR1/TCIM/SULF1/STK3/NDRG1/DOCK8/SLC1A1/DAPK1/KLF4/GSN/SLC27A4/NACC2/TRAF2/NSMF/FBH1/SGMS1/DKK1/IPMK/CDK1/RTKN2/AIFM2/DDIT4/IFIT2/ANKRD1/CALHM2/ITPRIP/BAG3/RASSF7/LRP5/ACER3/BIRC3/USP28/KMT2A/HYOU1/CBL/SORL1/ST14/ATN1/FGD4/WNT10B/LMBR1L/FAIM2/KRT80/KRT86/KRT83/KRT8/ESPL1/MAP3K12/ERBB3/PHLDA1/APAF1/DRAM1/HSP90B1/UBC/ZNF268/RGCC/MIR17HG/ING1/MCF2L/PCID2/TGM1/PRKD1/G2E3/EGLN3/NFKBIA/FERMT2/ARG2/BDKRB2/DIO3/HSP90AA1/THBS1/BUB1B/TYRO3/DAPK2/PLEKHO2/PARP16/SMAD3/SIN3A/IGF1R/PPL/PLK1/NUPR1/CORO1A/HERPUD1/CDH5/PLCG2/MEAK7/ATP2A3/CLDN7/TMEM102/MAPK7/SARM1/TOP2A/TNS4/KRT10/KRT34/KRT16/PRKCA/MAP2K6/SOX9/SOCS3/CBX4/IMPACT/ALPK2/MALT1/TRIB3/RBCK1/RASSF2/ID1/BCL2L1/TPX2/SRSF6/AURKA/GADD45B/ZBTB7A/TNFAIP8L1/CDKN2D/DNASE2/CALR/HSPB6/ZNF420/DPF1/MAP3K10/EGLN2/DEDD2/BCL3/MARK4/HIF3A/PRKD2/BBC3/C5AR1/CARD8/PTPRH/BID/PRODH/PRAME/MTFP1/PLA2G3/IL2RB/H1-0/BIK/PPARA/MX1 |
| GO:0043067 | regulation of programmed cell death | 177  /1509 | 0.036495375 | PRKCZ/TNFRSF25/CASP9/RPS6KA1/PRKAA2/GADD45A/CTH/CCN1/VTCN1/NTRK1/KIF14/BTG2/NUAK2/FCMR/ATF3/ITPKB/CYP1B1/SOS1/PELI1/TIA1/EIF2AK3/FHL2/NCK2/PAX8/STEAP3/GLI2/LIMS2/CSRNP3/SLC39A10/HSPD1/CREB1/TMBIM1/ACKR3/CAPN10/PRKCD/MITF/FOXP1/GATA2/SKIL/BCL6/SNCA/NDNF/SLC7A11/HMGB2/CARD6/PLK2/JMY/TNFAIP8/PDGFRB/PRR7/SQSTM1/PPP1R10/IER3/TNF/HSPA1B/SPDEF/PHIP/EPHA7/GRIK2/HEY2/SGK1/TNFAIP3/CITED2/AKAP12/ESR1/TIAM2/SOD2/RPS6KA2/IGFBP3/ASNS/ING3/HIPK2/FGD1/ARHGEF9/GPRASP2/ARHGEF6/GNRH1/PTK2B/RNF122/FGFR1/TCIM/STK3/NDRG1/DOCK8/DAPK1/KLF4/GSN/SLC27A4/NACC2/TRAF2/NSMF/FBH1/SGMS1/DKK1/CDK1/RTKN2/AIFM2/IFIT2/ANKRD1/CALHM2/ITPRIP/BAG3/LRP5/ACER3/BIRC3/HYOU1/CBL/FGD4/WNT10B/FAIM2/MAP3K12/ERBB3/PHLDA1/APAF1/HSP90B1/UBC/ZNF268/RGCC/MIR17HG/MCF2L/PCID2/EGLN3/NFKBIA/ARG2/BDKRB2/HSP90AA1/THBS1/TYRO3/DAPK2/SMAD3/SIN3A/IGF1R/PLK1/NUPR1/CORO1A/HERPUD1/CDH5/PLCG2/CLDN7/TMEM102/MAPK7/SARM1/TOP2A/PRKCA/MAP2K6/SOX9/SOCS3/CBX4/ALPK2/MALT1/RBCK1/RASSF2/ID1/BCL2L1/SRSF6/AURKA/GADD45B/ZBTB7A/TNFAIP8L1/CDKN2D/CALR/HSPB6/ZNF420/MAP3K10/EGLN2/DEDD2/BCL3/MARK4/BBC3/C5AR1/CARD8/BID/PRAME/PLA2G3/IL2RB/BIK/PPARA |
| GO:0012501 | programmed cell death | 231  /1509 | 0.036673728 | PRKCZ/TNFRSF25/CASP9/RPS6KA1/PRKAA2/GADD45A/CTH/CCN1/MAGI3/VTCN1/BNIPL/S100A14/TMEM79/NTRK1/DHX9/KIF14/BTG2/NUAK2/FCMR/ATF3/ITPKB/CYP1B1/SOS1/PELI1/TIA1/HK2/EIF2AK3/FHL2/NCK2/BUB1/PAX8/STEAP3/GLI2/LIMS2/PKP4/CSRNP3/GULP1/SLC39A10/HSPD1/BMPR2/CREB1/ERBB4/TMBIM1/ACKR3/CAPN10/PRKCD/MITF/FOXP1/GATA2/SKIL/BCL6/SNCA/NDNF/SLC7A11/HMGB2/CARD6/PLK2/JMY/TNFAIP8/SPINK5/PDGFRB/ANXA6/PRR7/SQSTM1/PPP1R10/IER3/TNF/HSPA1B/SPDEF/PHIP/EPHA7/GRIK2/TRAF3IP2/HEY2/SGK1/TNFAIP3/CITED2/PLAGL1/AKAP12/ESR1/TIAM2/SOD2/RPS6KA2/C6orf120/IGFBP3/ASNS/ING3/HIPK2/FGD1/ARHGEF9/GPRASP2/ARHGEF6/GNRH1/PTK2B/RNF122/FGFR1/TCIM/SULF1/STK3/NDRG1/DOCK8/SLC1A1/DAPK1/KLF4/GSN/SLC27A4/NACC2/TRAF2/NSMF/FBH1/SGMS1/DKK1/IPMK/CDK1/RTKN2/AIFM2/DDIT4/IFIT2/ANKRD1/CALHM2/ITPRIP/BAG3/RASSF7/LRP5/ACER3/BIRC3/USP28/KMT2A/HYOU1/CBL/ST14/ATN1/FGD4/WNT10B/LMBR1L/FAIM2/KRT80/KRT86/KRT83/KRT8/ESPL1/MAP3K12/ERBB3/PHLDA1/APAF1/DRAM1/HSP90B1/UBC/ZNF268/RGCC/MIR17HG/MCF2L/PCID2/TGM1/PRKD1/G2E3/EGLN3/NFKBIA/ARG2/BDKRB2/DIO3/HSP90AA1/THBS1/BUB1B/TYRO3/DAPK2/PLEKHO2/SMAD3/SIN3A/IGF1R/PPL/PLK1/NUPR1/CORO1A/HERPUD1/CDH5/PLCG2/ATP2A3/CLDN7/TMEM102/MAPK7/SARM1/TOP2A/TNS4/KRT10/KRT34/KRT16/PRKCA/MAP2K6/SOX9/SOCS3/CBX4/ALPK2/MALT1/TRIB3/RBCK1/RASSF2/ID1/BCL2L1/TPX2/SRSF6/AURKA/GADD45B/ZBTB7A/TNFAIP8L1/CDKN2D/DNASE2/CALR/HSPB6/ZNF420/DPF1/MAP3K10/EGLN2/DEDD2/BCL3/MARK4/HIF3A/BBC3/C5AR1/CARD8/PTPRH/BID/PRODH/PRAME/MTFP1/PLA2G3/IL2RB/H1-0/BIK/PPARA/MX1 |
| GO:0031109 | microtubule polymerization or depolymerization | 25  /1509 | 0.041076259 | PSRC1/CCSAP/MAPRE3/CLASP1/SNCA/TPPP/MAP1B/HSPA1B/AKAP9/KIF18A/CKAP5/FKBP4/NCKAP5L/SKA3/MZT1/NIN/TTBK2/TUBGCP4/BMERB1/CDH5/TUBG1/KIF18B/SKA2/TPX2/CAMSAP3 |
| GO:0016477 | cell migration | 171  /1509 | 0.041076259 | PRKCZ/MIIP/NBL1/ASAP3/PHACTR4/EDN2/SVBP/PIK3R3/GADD45A/CCN1/F3/CELSR2/S100A14/DDR2/GLUL/LAMC2/PTGS2/KIF14/PIK3C2B/MARK1/DUSP10/RHOU/LYST/CYP1B1/SOS1/SLC8A1/SEMA4C/NCK2/EPB41L5/CLASP1/MGAT5/RND3/FMNL2/BMPR2/ERBB4/DOCK10/ACKR3/PRKCD/MITF/FOXP1/PLXNA1/GATA2/PFN2/S100P/FGFBP1/RUFY3/IL8/CXCL3/NDNF/SLC7A11/GAB1/HMGB2/PLK2/DEPDC1B/MAP1B/ARSB/HBEGF/PDGFRB/ANXA6/SPARC/GCNT2/TNF/AGER/GPSM3/ANKS1A/SRF/LAMA2/CCN2/SGK1/PEX7/TNFAIP3/CITED2/AKAP12/SOD2/CEP43/AFDN/ANLN/IGFBP3/GPC2/EPHB4/DOCK4/TCAF2/CDKL5/MPP1/GNRH1/PTK2B/FGFR1/PLAT/SULF1/CPNE3/EXT1/TRIB1/PTP4A3/DOCK8/DDX58/TESK1/ARHGEF39/GCNT1/KLF4/LHX6/SCAI/NSMF/RASGEF1A/CDK1/PBLD/VSIR/DDIT4/KIF20B/LRP5/SORL1/ARHGDIB/ARID2/RND1/LIMA1/RHOF/ZNF268/STARD13/DCLK1/POSTN/RGCC/GPR183/SLC7A8/PRKD1/FERMT2/THBS1/TYRO3/TTBK2/DAPK2/ZNF609/SMAD3/CSPG4/PEAK1/DNAJA4/SEMA4B/IGF1R/BMERB1/ABCC1/CORO1A/ADGRG1/CDH5/RIPOR1/PLCG2/MEAK7/SPNS2/TMEM102/MAP2K3/FOXN1/GRB7/KRT16/MAP3K3/PRKCA/SOX9/ITGB4/FSCN2/LAMA1/ONECUT2/SERPINB3/THBD/ID1/NFATC2/INSR/CAMSAP3/CALR/SYDE1/PRKD2/C5AR1/MYADM/MBOAT7/GTSE1/ADAMTS1/AIRE |
| GO:0040011 | locomotion | 205  /1509 | 0.041076259 | PRKCZ/MIIP/NBL1/ASAP3/MACO1/PHACTR4/EDN2/SVBP/PIK3R3/GADD45A/CCN1/F3/CELSR2/S100A14/EFNA4/EFNA3/ASH1L/NTRK1/DDR2/GLUL/LAMC2/PTGS2/KIF14/PIK3C2B/MARK1/DUSP10/RHOU/CCSAP/LYST/CYP1B1/SOS1/SLC8A1/DOK1/SEMA4C/NCK2/EPB41L5/GLI2/CLASP1/MGAT5/RND3/FMNL2/BMPR2/CREB1/ERBB4/DOCK10/ACKR3/PRKCD/MITF/FOXP1/CFAP44/PLXNA1/GATA2/PFN2/S100P/FGFBP1/RUFY3/IL8/CXCL3/SLC9B2/NDNF/SLC7A11/GAB1/HMGB2/PLK2/DEPDC1B/MAP1B/ARSB/JMY/HBEGF/PDGFRB/ANXA6/SPARC/GCNT2/TNF/AGER/GPSM3/ANKS1A/SRF/EPHA7/LAMA2/CCN2/SGK1/PEX7/TNFAIP3/CITED2/AKAP12/SOD2/CEP43/AFDN/ANLN/IGFBP3/GPC2/EPHB4/DOCK4/GPR37/TCAF2/CDKL5/MPP1/GNRH1/PTK2B/FGFR1/PLAT/SULF1/CPNE3/EXT1/TRIB1/PTP4A3/DOCK8/VLDLR/RFX3/DDX58/TESK1/ARHGEF39/GCNT1/VPS13A/PTCH1/KLF4/LHX6/SCAI/NSMF/RASGEF1A/CDK1/PBLD/VSIR/DDIT4/ZSWIM8/KIF20B/SPTBN2/LRP5/NECTIN1/SORL1/ARHGDIB/INTS13/ARID2/RND1/LIMA1/ERBB3/CFAP54/RHOF/ZNF268/STARD13/DCLK1/POSTN/RGCC/MYCBP2/GPR183/SLC7A8/PRKD1/FERMT2/THBS1/TYRO3/TTBK2/DAPK2/ZNF609/SMAD3/CSPG4/PEAK1/DNAJA4/SEMA4B/IGF1R/NTN3/BMERB1/ABCC1/CORO1A/ADGRG1/CDH5/RIPOR1/PLCG2/MEAK7/SPNS2/CLDN7/TMEM102/MAPK7/MAP2K3/FOXN1/GRB7/KRT16/MAP3K3/PRKCA/SOX9/ITGB4/CEP131/FSCN2/LAMA1/ONECUT2/SERPINB3/THBD/ID1/PLTP/NFATC2/INSR/CAMSAP3/CALR/ZSWIM4/SYDE1/PRKD2/C5AR1/MYADM/MBOAT7/PLA2G3/TTLL1/GTSE1/SHANK3/ADAMTS1/AIRE |
| GO:0042981 | regulation of apoptotic process | 173  /1509 | 0.043176501 | PRKCZ/TNFRSF25/CASP9/RPS6KA1/PRKAA2/GADD45A/CTH/CCN1/VTCN1/NTRK1/KIF14/BTG2/NUAK2/FCMR/ATF3/ITPKB/CYP1B1/SOS1/TIA1/EIF2AK3/FHL2/NCK2/PAX8/STEAP3/GLI2/LIMS2/CSRNP3/SLC39A10/HSPD1/CREB1/TMBIM1/ACKR3/CAPN10/PRKCD/MITF/FOXP1/GATA2/SKIL/BCL6/SNCA/NDNF/SLC7A11/HMGB2/CARD6/PLK2/JMY/TNFAIP8/PDGFRB/PRR7/SQSTM1/PPP1R10/IER3/TNF/HSPA1B/SPDEF/PHIP/EPHA7/GRIK2/HEY2/SGK1/TNFAIP3/CITED2/AKAP12/ESR1/TIAM2/SOD2/RPS6KA2/IGFBP3/ASNS/ING3/HIPK2/FGD1/ARHGEF9/GPRASP2/ARHGEF6/GNRH1/PTK2B/RNF122/FGFR1/TCIM/STK3/NDRG1/DOCK8/DAPK1/KLF4/GSN/SLC27A4/NACC2/TRAF2/NSMF/FBH1/SGMS1/DKK1/CDK1/RTKN2/AIFM2/IFIT2/ANKRD1/CALHM2/ITPRIP/BAG3/LRP5/BIRC3/HYOU1/CBL/FGD4/WNT10B/FAIM2/MAP3K12/ERBB3/PHLDA1/APAF1/HSP90B1/UBC/ZNF268/RGCC/MIR17HG/MCF2L/PCID2/EGLN3/NFKBIA/ARG2/BDKRB2/HSP90AA1/THBS1/TYRO3/DAPK2/SMAD3/SIN3A/IGF1R/PLK1/NUPR1/CORO1A/HERPUD1/CDH5/CLDN7/TMEM102/MAPK7/SARM1/TOP2A/PRKCA/MAP2K6/SOX9/SOCS3/CBX4/ALPK2/MALT1/RBCK1/RASSF2/ID1/BCL2L1/SRSF6/AURKA/GADD45B/ZBTB7A/TNFAIP8L1/CDKN2D/CALR/HSPB6/ZNF420/MAP3K10/EGLN2/DEDD2/BCL3/BBC3/C5AR1/CARD8/BID/PRAME/PLA2G3/IL2RB/BIK/PPARA |
| GO:0030855 | epithelial cell differentiation | 84  /1509 | 0.043781047 | PTCH2/TMEM79/NTRK1/ELF3/GRHL1/PAX8/EPB41L5/PKP4/FZD7/BMPR2/CREB1/ERBB4/ABCA12/SLC4A7/ZBED2/PLS1/SKIL/SPRY1/INTU/SLC7A11/SPINK5/BMP6/TNF/SPDEF/HEY2/ESR1/AFDN/HEY1/EXT1/RFX3/ZDHHC21/AQP3/PTCH1/KLF4/TPRN/CDK1/ACTA2/PDZD7/DMBT1/USH1C/E2F8/PAX6/EHF/CCND1/EXPH5/ST14/WNT10B/KRT80/KRT86/KRT83/KRT8/E2F7/SCEL/PCK2/TGM1/ARID4A/SMAD3/CYP1A1/CCDC78/DNASE1L2/PPL/CDH5/KDM6B/TRIM16/FOXN1/KRT10/KRT34/KRT16/HOXB5/SOX9/MAFG/LAMA1/ONECUT2/ID1/CBFA2T2/SRSF6/FZR1/TJP3/CAMSAP3/KLF2/SIPA1L3/MYADM/LIF/TRIOBP |
| GO:0006357 | regulation of transcription by RNA polymerase II | 275  /1509 | 0.04736673 | CASZ1/ZBTB40/RPS6KA1/ARID1A/ZBTB8A/HEYL/CITED4/HIVEP3/KTI12/EFCAB7/GADD45A/CCN1/SARS1/RORC/ZBTB7B/ASH1L/DHX9/ELF3/BTG2/ATF3/ZNF692/GRHL1/EPAS1/REL/MXD1/KDM3A/ARID5A/FHL2/NCK2/PAX8/GLI2/CSRNP3/HSPD1/BMPR2/CARF/CREB1/PER2/ZNF852/ZNF35/ZNF501/ABHD14B/MITF/FOXP1/ZNF654/BBX/ZBED2/GATA2/SKIL/TBL1XR1/YEATS2/BCL6/PCGF3/SNCA/TET2/HMGB2/IRF2/JMY/BRD8/TAF7/TNIP1/CREBRF/SQSTM1/BMP6/ATXN1/POU5F1/TNF/PBX2/SPDEF/TEAD3/FOXP4/MDFI/SRF/RUNX2/PHIP/ZNF292/FRK/HEY2/NCOA7/CITED2/PLAGL1/ESR1/ARID1B/SOD2/SP4/NFE2L3/HOXA6/CREB5/MLXIPL/BHLHA15/TSC22D4/HIPK2/KMT2C/MNX1/ZBED1/TBL1X/ZNF630/TFE3/PHF8/BRWD3/PHF6/MAMLD1/PLAG1/MYBL1/NCOA2/HEY1/ZBTB10/TRPS1/ZNF572/ZNF250/FOXD4/RFX3/DDX58/KLF9/NFIL3/PTCH1/CAVIN4/KLF4/LHX6/PBX3/BRD3/RXRA/NACC2/ZNF33B/ZNF485/DKK1/JMJD1C/ATOH7/ANKRD1/ANKRD2/NFKB2/SUFU/MXI1/BAG3/ZNF214/E2F8/PAX6/EHF/CRY2/PHF21A/RCOR2/MEN1/PPP2R5B/RBM14/LRP5/CCND1/IL18/KMT2A/CDON/PRDM10/CHD4/ATN1/BHLHE41/WNT10B/KMT2D/ATF7/CALCOCO1/HOXC6/HOXC9/SMARCC2/E2F7/ELK3/UBC/ZNF140/ZNF10/ZNF268/RGCC/PRKD1/EGLN3/NFKBIA/ARID4A/ZBTB42/MEIS2/TP53BP1/RORA/ZNF609/SMAD3/ARID3B/SIN3A/ZNF75A/PLK1/FBXL19/ZNF668/PSMB10/NFAT5/ZNF19/ZNF821/FOXL1/ZBTB4/KDM6B/HES7/PER1/ZNF18/MAPK7/FOXN1/TOP2A/KAT2A/HOXB3/HOXB4/HOXB5/HOXB6/HLF/BRIP1/SOX9/CBX4/MAFG/TGIF1/IMPACT/ONECUT2/TRIB3/MAVS/ID1/CBFA2T2/PHF20/MAFB/LPIN3/NFATC2/TSHZ2/ZNF217/HELZ2/ZBTB46/TCEA2/FSTL3/ARID3A/ZBTB7A/ZNF414/ZNF121/ZNF653/ZNF823/ZNF441/JUNB/CALR/NACC1/RFX1/DNAJB1/KLF2/ZNF14/ZNF420/ZNF569/DPF1/PSMC4/ZNF780B/EGLN2/ERF/CIC/ZNF575/BCL3/MYPOP/HIF3A/PRKD2/NPAS1/MAMSTR/ZNF615/ZNF841/ZNF616/ZNF528/ZNF816/ZNF331/ZNF548/ZNF17/ZNF772/ZNF416/ZNF530/ZNF134/ZNF587B/ZNF417/ZNF274/ZNF837/LIF/H1-0/CBX6/TEF/PPARA/NRIP1/ERG/AIRE |

**Table S7**. The most significantly affected upstream regulators and their target genes after 2 hours infection with *H. pylori* wt.

| **Upstream Regulator** | **Expr FDR (q-value)** | **Activation z-score** | **p-value of overlap** | **Target Molecules in Dataset** |
| --- | --- | --- | --- | --- |
| **Activated** | | | | |
| EGFR | 2.83E-12 | 2.295 | 5.82E-08 | CEBPB, IL8, EGR1, ICAM1, JUN, MUC5AC, NFKBIA, PTGS2, SOX9 |
| EGR1 | 5.55E-05 | 2.400 | 5.80E-10 | ATF3, CDKN1A, IL8, EREG, FOSL1, GDF15, THBS1, TNF |
| FOXL2 | 2.81E-02 | 3.308 | 6.99E-14 | ATF3, CXCL2, CXCL3, ICAM1, IER3, LIF, MAFF, PPP1R15A, PTGS2, SOD2, TNFAIP3 |
| FOXO3 | 1.18E-03 | 2.167 | 4.46E-10 | CCNG2, CDKN1A, IL8, EGR1, FBXO32, IER3, NFKBIA, SOD2, TNF |
| IL1A | 3.51E-66 | 3.216 | 5.77E-13 | ALDH1A3, CDKN1A, CXCL2, CXCL3, IL8, IL1A, NFKBIA, PTGS2, SOD2, TNF, TNFAIP3 |
| IL1B | 1.50E-06 | 3.262 | 6.66E-12 | CEBPB, CXCL2, IL8, EGR1, ICAM1, IL1B, IL1RN, MUC5AC, NFKBIA, PTGS2, REL, RXRA, SOD2, TNF |
| RELA | 2.19E-03 | 3.652 | 1.91E-20 | BIRC3, BTG2, CDKN1A, CEBPB, CXCL2, CXCL3, IL8, EGR1, GLI2, ICAM1, IL1A, IL1B, IRF1, MUC5AC, NFKBIA, NR4A2, PTGS2, SOD2, SOX9, TNF, TNFAIP3 |
| TNF | 7.74E-05 | 5.558 | 1.08E-31 | ALDH1A3, BCL3, BIRC3, BTG2, CDKN1A, CXCL2, CXCL3, IL8, EFNA1, EGR1, ETS1, ICAM1, IER3, IL1A, IL1B, IL1RN, IRF1, JUN, MAFF, MUC5AC, NFKB1, NFKBIA, NFKBIE, PMAIP1, PPP1R15A, PRDM1, PTGS2, RXRA, SDC4, SOD2, SOX9, TGFA, TNF, TNFAIP2, TNFAIP3, TRAF1, VIPR1, WNT5A |
| **Inhibited** | | | | |
| EFNA5 | 4.71E-17 | -2.000 | 2.14E-04 | DUSP4, FOSL1, IRF1, SOX9 |
| FAS | 4.71E-02 | -2.944 | 1.02E-23 | BIRC3, CEBPD, IL8, EFNA1, ICAM1, IER3, IRF1, NFKBIA, NFKBIE, NUAK2, PPP1R15A, RND1, SOD2, TNFAIP3, ZFP36 |
| WWTR1 | 1.27E-02 | -2.000 | 1.41E-05 | CDKN1A, IL8, IL1A, IL1B |

**Table S8**. The most significantly affected upstream regulators and their target genes after 2 hours infection with *H. pylori* Δ*htrA*.

| **Upstream Regulator** | **Expr FDR (q-value)** | **Activation z-score** | **p-value of overlap** | **Target Molecules in Dataset** |
| --- | --- | --- | --- | --- |
| **Activated** | | | | |
| EGFR | 3.25E-14 | 2.085 | 2.65E-06 | CEBPB, IL8, EGR1, ICAM1, JUN, MUC5AC, NFKBIA, PTGS2 |
| EGR1 | 3.55E-03 | 2.400 | 2.04E-09 | ATF3, CDKN1A, IL8, EREG, FOSL1, GDF15, THBS1, TNF |
| FOXL2 | 1.65E-03 | 2.585 | 2.54E-16 | AMH, ATF3, CXCL2, CXCL3, ICAM1, IER3, LIF, MAFF, PPP1R15A, PTGS2, RGS2, SOD2, TNFAIP3 |
| FOXO1 | 3.68E-02 | 2.609 | 5.72E-12 | CCNG2, CDKN1A, IL8, EGR1, FBXO32, IER3, IGFBP1, PMAIP1, POU5F1, PRDM1, TNF |
| FOXO3 | 7.88E-05 | 2.167 | 1.81E-09 | CCNG2, CDKN1A, IL8, EGR1, FBXO32, IER3, NFKBIA, SOD2, TNF |
| GPER1 | 3.95E-02 | 3.464 | 7.95E-16 | ATF3, DUSP4, EGR1, JUN, NFKBIA, NR4A2, PPP1R15A, PTGER4, RND1, TNF, TNFAIP3, ZFP36 |
| IL1A | 3.88E-71 | 3.356 | 1.09E-13 | ALDH1A3, CDKN1A, CXCL2, CXCL3, IL8, IL1A, NFKBIA, PTGS2, SOD2, TNF, TNFAIP3, ZC3H12A |
| IL1B | 1.59E-05 | 3.262 | 5.69E-11 | CEBPB, CXCL2, IL8, EGR1, ICAM1, IL1B, IL1RN, MUC5AC, NFKBIA, PTGS2, REL, RXRA, SOD2, TNF |
| RELA | 1.11E-04 | 3.265 | 2.74E-20 | ANKRD1, BIRC3, BTG2, CDKN1A, CEBPB, CXCL2, CXCL3, IL8, EGR1, GLI2, ICAM1, IL1A, IL1B, IRF1, MUC5AC, NFKB2, NFKBIA, NR4A2, PTGS2, SOD2, TNF, TNFAIP3 |
| SYVN1 | 2.23E-03 | 2.236 | 5.47E-03 | CYP1B1, ERCC6, IRAK2, NFKB2, SLC6A6 |
| TNF | 5.50E-10 | 5.647 | 4.11E-30 | ALDH1A3, BCL3, BIRC3, BTG2, CDKN1A, CXCL2, CXCL3, IL8, EFNA1, EGR1, ETS1, ICAM1, IER3, IL1A, IL1B, IL1RN, IRF1, JUN, MAFF, MUC5AC, NFKB1, NFKB2, NFKBIA, NFKBIE, PMAIP1, PPP1R15A, PRDM1, PTGS2, RGS2, RXRA, SDC4, SOD2, TGFA, TNF, TNFAIP2, TNFAIP3, TRAF1, VIPR1, WNT5A |
| **Inhibited** | | | | |
| IL1RN | 6.85E-20 | -2.138 | 1.66E-07 | ATF3, ICAM1, IL18BP, IL1A, IRF1, KCTD7, PMAIP1, TNF |
| S100A6 | 1.20E-02 | -2.000 | 7.42E-05 | ATF3, CXCL2, IL8, TNFAIP3 |
| WWTR1 | 9.67E-03 | -2.000 | 2.63E-05 | CDKN1A, IL8, IL1A, IL1B |

**Table S9.** The most significantly affected upstream regulators and their target genes after 6 hours infection with *H. pylori* wt.

| **Upstream Regulator** | **Expr FDR (q-value)** | **Activation z-score** | **p-value of overlap** | **Target Molecules in Dataset** |
| --- | --- | --- | --- | --- |
| **Activated** | | | | |
| ATF6 | 2.91E-03 | 2.263 | 1.20E-03 | AURKA, BUB1, DAPK1, DNAJB11, HSP90B1, HSPA5, MAP1LC3B, PDIA4, TROAP |
| CIP2A | 7.86E-31 | 2.000 | 7.17E-04 | CIP2A, CYSRT1, E2F2, GADD45A, GPR183, GPR37, HK2, KIAA1324, LAMA1, NPTX1, PDGFRB, PDLIM7, PXYLP1, RAB31, SLC12A8, SNCA |
| ECSIT | 1.70E-09 | 2.400 | 4.80E-02 | BCL3, IL8, JUNB, NFKB2, PTGS2, SOD2 |
| FOXM1 | 4.82E-24 | 2.146 | 5.95E-04 | AURKA, BUB1B, CCNA2, CCNB1, CCND1, CDK1, CENPA, CENPF, ESR1, GTSE1, KIF20A, NFKB2, PDGFA, PLK1, PTCH1, PTCH2 |
| MITF | 1.53E-15 | 2.637 | 2.15E-03 | BEST1, CCNB1, CCNF, CCNG2, CDCA3, CENPF, CENPH, ESPL1, FBXO32, KIF20A, KIF4A, KIFC1, NCAPD3, PIF1, PLK1, SPAG5, TACC3, TERT, TPX2 |
| MYBL2 | 8.44E-05 | 2.412 | 1.32E-04 | AURKA, BUB1, CCNA2, CCNB1, CDK1, CENPE, PLK1 |
| MYC | 9.18E-27 | 3.161 | 1.43E-04 | ABCC1, ADAMTS1, ATP13A2, BAX, BBC3, BCAT1, BCL2L1, BCL6, CAD, CBX4, CCNB1, CCNE1, CCNG2, DDB2, E2F2, FBXO32, GADD45A, GADD45B, HK2, HOXA5, HSP90AA1, HSPB1, HSPD1, HSPH1, ID1, IDH1, IFIT1, IFIT2, IFIT3, IFIT5, ISG20, LOC102724788/PRODH, MIR17HG, MITF, MX1, OASL, PDGFRB, PEG10, PHF20, PHF21A, PIAS2, RARRES1, SLC25A21, SLC2A1, TERT, TRIM5, ULBP1, USP54 |
| TRAF2 | 2.33E-22 | 2.429 | 2.82E-03 | AURKA, CCNB1, CDC25C, CDK1, IL8, PLK1 |
| **Inhibited** | | | | |
| CDKN1A | 4.11E-04 | -2.179 | 1.37E-04 | BAX, BCL2L1, CCNA2, CCNB1, CCND1, CDC25C, CDK1, DUSP1, FN1, LBR, LIMA1, MBNL2, PCGF3, PLK1, SOX9, TOP2A |
| DUSP1 | 6.57E-31 | -2.383 | 2.24E-02 | IL8, DKK1, DUSP1, GDF15, PLAT, PTGS2, THBD |
| GPER1 | 1.88E-05 | -2.254 | 3.95E-07 | ATF3, CCN1, CCN2, DDIT4, DUSP1, DUSP5, EDN1, EDN2, ESR1, ITPRIP, MT1X, MT2A, RASD1, RGS16, RND1, TNF, TUFT1 |
| KDM5B | 3.94E-19 | -2.053 | 1.27E-04 | AURKA, BUB1B, CCNB1, CCND1, CDCA3, CDK1, DLGAP5, EPB41L1, FHL1, FJX1, GADD45A, HSD17B8, KIF2C, MT1X, NCAPH, NDC80, NEDD9, PHF20, PSD3, SCNN1A, SMOX, SOX9, TOP2A, TTK |
| MAVS | 2.30E-21 | -2.220 | 1.53E-02 | IL8, HERPUD1, IFIT1, MAT2A, SOCS3 |
| MRTFB | 1.17E-37 | -2.891 | 1.76E-02 | COL25A1, EDN1, EDNRA, FJX1, GPR37, ID1, MTMR10, NREP, SELENOP, SLFN5, SULF1, THBS1 |
| NR3C1 | 7.97E-30 | -2.533 | 7.91E-04 | ALOX5AP, ATXN1, BAG3, BCL2L1, BCL6, C1QTNF1, CARD6, CASP9, CAVIN2, CBX4, CTNNBL1, IL8, DAPK2, DEDD2, EDN2, ELMOD3, FASTKD1, FGD4, GAB1, GADD45A, GADD45B, GLP2R, GLUL, HSPA6, IGF1R, IL18, ING3, MAGI3, MALT1, MAP3K14, MAPK7, NFKB2, NTRK1, PIK3R3, PLAGL1, PLEKHF1, PLK2, POU5F1, PPP1R13L, PRKACB, PRKAR1B, PRKCD, PTGS2, PTK2B, RELT, RGS2, RHOB, S100P, SCNN1A, SGK1, SNCA, THBD, TNF, TNFAIP8, TNFRSF25, TNS4, TRAF3IP2, TRIB3, TRIM45, WDR31, ZNF346 |
| NUPR1 | 3.78E-59 | -4.210 | 2.25E-24 | ABCC5, ABHD15, ADAM22, ADGRG1, AKAP12, ANGEL1, ASB9, ASIC1, ASPM, ATF3, AURKA, BRI3BP, BRINP3, BUB1, BUB1B, C1orf112, C3orf62, CAMK2N1, CARMIL1, CCDC134, CCDC77, CCN1, CCNA2, CCNF, CDC25C, CDCA2, CDCA3, CENPI, CERK, CHD4, CHMP1B, CITED2, CKAP2L, COA5, CTPS2, IL8, DUSP5, DUSP8, E2F8, ELL2, ENO2, EPHA7, ESPL1, FAM72C/FAM72D, FAM83G, FHL2, FUT11, GABBR1, GADD45A, GBP2, GCNT2, GDF15, GK, GPCPD1, GPR1, GSTA4, GTSE1, HBEGF, HIVEP3, HJURP, HK2, HOXB5, HSPA2, IFIT2, IGF1R, KANK2, KDM3A, KIF18A, KIF20A, KIF2C, KIFC1, KLF4, LARS2, LBR, LHFPL2, LIF, LMAN2L, MAFG, MAT2A, MKI67, MRTFB, MT1X, MXD1, NAA40, NDRG1, NFIL3, NRBF2, NRSN2, NT5DC3, OSBPL6, P4HA2, PCTP, PER3, PFKFB4, PHLDA1, PLK1, PLK3, PM20D2, POLH, POLQ, PXDC1, RAB17, RAB29, RASAL2, RBM14, RIMKLA, RNF122, SAMHD1, SKA2, SLC2A1, SPAG5, SRF, ST3GAL2, STIL, STK38, TCAF2, TCEANC2, TESK1, TICRR, TMEM158, TNFAIP8L1, TNIP1, TOB1, TP53BP1, TRIB1, TRIB3, TRIM16, UBIAD1, UCA1, UNC5B, ZMYM3 |
| TAZ | 1.48E-03 | -2.236 | 2.18E-04 | ANKRD1, CCN1, CCN2, EDN1, PPP1R3B |
| TEAD1 | 2.25E-11 | -2.259 | 2.15E-02 | CCND1, EDN1, EDNRA, FJX1, GPR37, MTMR10, SLFN5, SULF1, THBS1, TP63 |
| TEAD2 | 3.76E-02 | -2.828 | 3.99E-02 | EDN1, EDNRA, FJX1, GPR37, MTMR10, SLFN5, SULF1, THBS1 |
| TEAD3 | 2.08E-47 | -2.030 | 1.72E-02 | EDN1, EDNRA, FJX1, GPR37, MTMR10, SLFN5, SULF1, THBS1, TP63 |
| TEAD4 | 2.92E-02 | -2.259 | 1.86E-02 | CCND1, EDN1, EDNRA, FJX1, GPR37, MTMR10, SLFN5, SULF1, THBS1, TP63 |

**Table S10.** The most significantly affected upstream regulators and their target genes after 6 hours infection with *H. pylori* Δ*htrA*.

| **Upstream Regulator** | **Expr FDR (q-value)** | **Activation z-score** | **p-value of overlap** | **Target Molecules in Dataset** |
| --- | --- | --- | --- | --- |
| **Activated** | | | | |
| CBX5 | 2.02E-04 | 2.399 | 1.52E-02 | AGR2, CDC6, CPA4, CYP1B1, FGFBP1, HENMT1, HOXB6, IGFL2-AS1, KRT80, MAL2, PRSS23, RORA, RUNX2, S100P, SELENBP1, SLC2A3, SLC7A8, TIMP4, TM4SF1, ZSCAN16-AS1 |
| ECSIT | 1.21E-07 | 3.268 | 4.15E-05 | BCL3, IL8, IER3, JUNB, NFKB2, NFKBIA, NR4A2, PTGS2, SOD2, TNFAIP3, TRAF1 |
| FOXM1 | 1.44E-20 | 2.490 | 2.70E-03 | AURKA, BUB1B, CCNA2, CCNB1, CCND1, CDK1, CENPA, CENPF, ESR1, GTSE1, KIF20A, NFKB2, PLK1, PTCH1, PTCH2 |
| MITF | 1.55E-18 | 2.481 | 7.91E-03 | BEST1, CCNB1, CCNF, CCNG2, CDCA3, CENPF, CENPH, ESPL1, FBXO32, KIF20A, KIF4A, KIFC1, NCAPD3, PIF1, PLK1, SPAG5, TACC3, TPX2 |
| MYBL2 | 4.08E-06 | 2.412 | 1.73E-04 | AURKA, BUB1, CCNA2, CCNB1, CDK1, CENPE, PLK1 |
| MYC | 2.27E-30 | 2.656 | 1.29E-05 | ABCC1, ADAMTS1, ATP13A2, BAX, BBC3, BCAT1, BCL2L1, BCL6, BMI1, CAD, CBX4, CCNB1, CCNE1, CCNG2, DDB2, E2F2, FBXO32, GADD45A, GADD45B, HK2, HOXA5, HSP90AA1, HSPB1, HSPD1, ID1, IDH1, IFIT1, IFIT2, IFIT3, IGF2BP1, ISG20, LOC102724788/PRODH, MIR17HG, MITF, MX1, NFKBIA, OASL, PDGFRB, PEG10, PHF20, PHF21A, PIAS2, PMAIP1, RARRES1, SLC22A4, SLC25A21, SLC2A1, ST3GAL1, TRIM5, ULBP1, USP54, WNT7B, XPO1 |
| TNF | 3.90E-07 | 2.138 | 1.10E-04 | A4GALT, BBC3, BCL2L1, BCL3, BID, BIK, BIRC3, BTG2, CCND1, CDC25C, CDC42EP4, CHST2, CLASP1, CXCL3, IL8, DUSP1, DUSP10, DUSP5, EDN1, EHD1, EHF, ELF3, EXT1, F2RL1, GADD45A, HBEGF, HSPG2, IER3, IFIT3, IL1A, IL32, KRT34, LAMC2, MITF, NCOA2, NEDD9, NFKB2, NFKBIA, OASL, OSMR, P2RY6, PLA2G3, PMAIP1, PML, PPARA, PPP1R15A, PPP1R3C, PRSS23, PSMB10, PTGS2, RGS2, RND3, RXRA, SELENOP, SERPINB1, SLC16A5, SOD2, SOX9, SQSTM1, SYNPO, TAP1, TCIM, TM4SF1, TNF, TNFAIP3, TPST1, TRAF1, TRAF2, VIPR1 |
| TRAF2 | 1.02E-20 | 2.621 | 5.33E-04 | AURKA, BIRC3, CCNB1, CDC25C, CDK1, IL8, PLK1 |
| **Inhibited** | | | | |
| CDKN1A | 8.40E-03 | -2.431 | 2.26E-04 | BAX, BCL2L1, CCNA2, CCNB1, CCND1, CDC25C, CDK1, DUSP1, LBR, LIMA1, MBNL2, PCGF3, PLK1, RB1, SOX9, TOP2A |
| CLU | 2.27E-04 | -2.200 | 2.32E-03 | ATP7A, ATP7B, BAX, BCL2L1, CDK1, SMAD3 |
| DUSP1 | 1.06E-24 | -2.383 | 2.76E-02 | IL8, DKK1, DUSP1, GDF15, PLAT, PTGS2, THBD |
| KDM5B | 2.10E-23 | -2.211 | 9.14E-05 | AURKA, BUB1B, CCNB1, CCND1, CDCA3, CDK1, DLGAP5, EHD1, EPB41L1, FHL1, FJX1, GADD45A, HMMR, HSD17B8, MT1X, NCAPH, NDC80, NEDD9, PHF20, PSD3, SCNN1A, SMOX, SOX9, TOP2A, TTK |
| KLF5 | 2.75E-08 | -2.207 | 9.83E-02 | ALPI, BCL2L1, CCND1, DUSP1, FGFBP1 |
| MAVS | 1.90E-20 | -2.220 | 1.82E-02 | IL8, HERPUD1, IFIT1, MAT2A, SOCS3 |
| MRTFB | 1.28E-41 | -2.891 | 2.39E-02 | COL25A1, EDN1, EDNRA, FJX1, GPR37, ID1, MTMR10, NREP, SELENOP, SLFN5, SULF1, THBS1 |
| NR3C1 | 1.75E-32 | -2.421 | 2.32E-06 | ALOX5AP, ANXA1, ATXN1, BAG3, BCL2L1, BCL6, BIRC3, BMF, C1QTNF1, C1QTNF2, CARD6, CASP9, CAVIN2, CBX4, CKAP2, CTNNBL1, IL8, DAPK2, DEDD2, EDN2, ELMOD3, FASTKD1, FGD4, GAB1, GADD45A, GADD45B, GLP2R, GLUL, GULP1, HSPA6, IER3, IGF1R, IL18, ING1, ING3, MAGI3, MALT1, MAP3K14, MAPK7, NFKB2, NFKBIA, NTRK1, PHLDA2, PIK3R3, PLAGL1, PLK2, POU5F1, PPP1R15A, PRKACB, PRKAR1B, PRKCD, PTGS2, PTK2B, RELT, RGS2, RHOB, S100P, SCNN1A, SGK1, SNCA, THBD, TNF, TNFAIP3, TNFAIP8, TNFRSF25, TNS4, TP53BP2, TRAF1, TRAF3IP2, TRIB3, TRIM45, WDR31, ZNF346 |
| NUPR1 | 6.12E-57 | -3.890 | 3.65E-25 | ABCC5, ABHD15, ADAM22, ADGRG1, AKAP12, ANP32E, ARHGAP11A, ASB9, ASIC1, ASPM, ATF3, AURKA, BNIP3, BRI3BP, BRINP3, BUB1, BUB1B, C19orf33, C1orf112, C3orf62, CAMK2N1, CARD8, CARMIL1, CCDC134, CCDC77, CCN1, CCNA2, CCNF, CDC25C, CDCA2, CDCA3, CENPI, CEP97, CHD4, CITED2, CKAP2L, COA5, COQ10A, CREB5, CXCL3, IL8, DUSP5, DUSP8, E2F8, ENO2, EPHA7, ESPL1, FAM72C/FAM72D, FAM83G, FGF1, FHL2, FUT11, GADD45A, GBP2, GCNT2, GDF15, GK, GPCPD1, GPR1, GTSE1, HBEGF, HIVEP3, HJURP, HK2, HOXB5, HSPA2, IFIT2, IGF1R, KANK2, KDM3A, KIF11, KIF18A, KIF20A, KIFC1, KLF4, LARS2, LBR, LHFPL2, LIF, LMAN2L, MAFG, MAT2A, MKI67, MRTFB, MT1X, MXD1, NDRG1, NEIL3, NFIL3, NRBF2, NRSN2, NT5DC3, OSBPL6, P4HA2, PAX8-AS1, PCTP, PER3, PFKFB4, PHLDA1, PLK1, PM20D2, POLH, POLQ, PPP1R15A, PXDC1, RAB17, RAB29, RASAL2, RBM14, RIMKLA, RNF122, RTN4IP1, SAMHD1, SKA2, SLC2A1, SPAG5, SRF, ST3GAL2, STIL, STK38, TCAF2, TCEANC2, TESK1, TICRR, TMEM158, TNFAIP8L1, TNIP1, TOB1, TP53BP1, TP53BP2, TRIB1, TRIB3, TRIM16, UBIAD1, UCA1, ZMYM3 |
| SP1 | 1.97E-02 | -2.248 | 1.31E-03 | ADAMTS1, ASNS, ATF3, BAX, CBS/CBSL, CCND1, CDC42BPG, CDK1, CDKN2D, CTH, IL8, EIF2AK2, ESR1, GDF15, HBEGF, HDAC4, HK2, HSPA5, IGF1R, IGFBP3, IL2RB, INSR, KRT16, KRT81, MAT2A, MAT2B, NAB2, NTRK1, PADI1, PIGM, PMAIP1, PRKCA, PTGS2, RB1, SLC19A1, SLC22A4, SLC2A1, SLC4A7, SMAD3, SOD2, SOX9, TNF, TRIB1, WNT9A, ZFAS1 |
| TAZ | 6.80E-06 | -2.236 | 2.68E-04 | ANKRD1, CCN1, CCN2, EDN1, PPP1R3B |
| TCF7L2 | 8.88E-05 | -2.000 | 2.29E-02 | ADGRG1, ANXA1, AQP3, CAMK2N1, CCND1, CLDN2, EPAS1, KDM4B |
| TEAD1 | 4.08E-12 | -2.353 | 2.81E-02 | CCND1, EDN1, EDNRA, FJX1, GPR37, MTMR10, RB1, SLFN5, SULF1, THBS1 |
| TEAD2 | 4.85E-04 | -2.828 | 4.96E-02 | EDN1, EDNRA, FJX1, GPR37, MTMR10, SLFN5, SULF1, THBS1 |
| UBE2I | 2.38E-18 | -2.000 | 4.87E-02 | PML, SGK1, SLC51B, SOX9, TAP1 |

**Table S11**. Primers used for RT-qPCR.

| Primer | Sequence |
| --- | --- |
| ZFP36-for | CACCCTCACCCACTTCGC |
| ZFP36-rev | TCTGAGAAGGTCCGACATAG |
| TNF-for | GTCCTCTTCAAGGGCCAAG |
| TNF-rev | CTCTTGATGGCAGAGAGGAG |
| NFKBIA-for | GGTTTTCTAGTGTCAGCTGG |
| NFKBIA-rev | GTCACTCCTGTTGAAGTGTG |
| IL8-for | GCCAAGGAGTGCTAAAGAAC |
| IL8-rev | GTCCACTCTCAATCACTCTCA |
| DKK1-for | GTCTTTGTCGCGATGGTAGC |
| DKK1-rev | CAGGTTCTTGATAGCGTTGG |
| DOCK8-for | GCCAGTGGACTTTGAAGGAC |
| DOCK8-rev | GAACACCACGTCCAAGTCG |
| GAPDH-for | GTATCGTGGAAGGACTCATG |
| GAPDH-rev | GAAAGCCAGTCCCCAGAAC |
